# Supplementary material for: Qualitative analysis to identify determinants of use among different occupational settings and channels of communication to address smokeless tobacco use in Sri Lanka
Source: PLOS Glob Public Health. 2023 Jan 4;3(1):e0001349. doi: 10.1371/journal.pgph.0001349 (PMC10022322; doi:10.1371/journal.pgph.0001349)
Supplement: S1 File — (DOC) [file pgph.0001349.s001.doc]

**Focus Group Discussion Guidelines:** **Qualitative analysis to Identify determinants of use among different occupational settings and channels of communication to address smokeless tobacco use in Sri Lanka**

1 Well come and introduction

2 Background

3 Ground rules

4 Opening question

Obtain the implied consent from all participants

Assess the pattern of use of SLT before start the FGDs

**Guiding question for FGD with Bus drivers ( ask one or two questions from each category)**

**Determinants of SLT**

How do you use it? Individually or with friends?

Are these SLT related to your job or your local neighborhood?

Why do you use it? Any special reason?

**Perception and Attitude**

What are satisfaction you gain out of using theme?

How do you compare the SLT with other substances?

Do you think that you are addicted to these SLT?

Any other positive aspects of using these SLT?

**Control Methods**

What are best methods that can be controlled as you know?

Are there any actions taken by religious organizations to control it?

Any health or medical level campaign to control it ?

Are there any politically powerful groups that promote the business of ST in your areas?

**Communication methods**

Have you heard the danger of SLT? If yes from whom or which source

What is the best communication method to reach you

What are the tools: dimensions, type and size

Do you use smart phone or mobile phone

Do you see SMS messages daily

Do you see TV, Radio which channel and time, programme

Are you in face book or any social media

Do you like to quit the habits

If not why

Whom do you trust. Religious leader

Do you believe if victims address you

Any other views or ideas about?

All information given by the respondent will be recorded using an audio recorder as well as written down in a designed record sheet. It is expected to complete FGD within 1½hrs.
