# Supplementary material for: Qualitative analysis to identify determinants of use among different occupational settings and channels of communication to address smokeless tobacco use in Sri Lanka
Source: PLOS Glob Public Health. 2023 Jan 4;3(1):e0001349. doi: 10.1371/journal.pgph.0001349 (PMC10022322; doi:10.1371/journal.pgph.0001349)
Supplement: S2 File — (DOC) [file pgph.0001349.s002.doc]

**In-depth interview Guidelines:** **Qualitative analysis to Identify determinants of use among different occupational settings and channels of communication to address smokeless tobacco use in Sri Lanka**

General Information

Background (education, family, neighborhood)

Employment or livelihood

General life experience (problems, social life, religion)

**Tobacco Issue**

Have you ever smoked cigarette or any other things? (if yes)How long and why?

Have you ever taken any type of alcohol? (if yes)How long and why?

Have you used any other substance?

Have you ever used smokeless tobacco? Or do you use smokeless tobacco?

What types of such smokeless tobacco you use?

How are they named or called locally in your area?

How did you come to know about this at first?

**Pattern of Use**

How frequently you them?

How long you have been using these SLT?

Why do you use it? Any special reason?

Can you pls tell the amount you use per day or week?

From where do you get these SLT?

How do you use it? Individually or with friends?

Are these non-smoke tobacco related to your job or your local neighborhood?

**Perception and Attitude**

What satisfaction you gain out of using theme?

How do you compare the SLT with other substances?

Do you think that you are addicted to these SLT?

Any other positive aspects of using these SLT?

Do you know any effects of these SLT (on economy, individual health, family)?

**Control Methods**

Are there any actions taken by religious organizations to control it?

Any health or medical level campaign to control it ?

Have you been hated or condemned by any other social forces?

Are there any politically powerful groups that promote the business of ST in your areas?

What are best methods that can be controlled as you know?

**Communication methods**

Have you heard the danger of SLT? If yes from whom or which source

What is the best communication method to reach you

What are the tools- dimensions, type and size

Do you use smart phone or mobile phone

Do you see SMS messages daily

Do you see TV, Radio which channel and time, programme

Are you in face book or any social media

Do you like to quit the habits

If not why

Whom do you trust. Religious leader

Do you belive if victims address you

Any other views or ideas about?

All information given by the respondent will be recorded using an audio recorder as well as written down in a designed record sheet. It is expected to complete an interview within 2 to 2 ½hrs.
