# Supplementary material for: Qualitative analysis to identify determinants of use among different occupational settings and channels of communication to address smokeless tobacco use in Sri Lanka
Source: PLOS Glob Public Health. 2023 Jan 4;3(1):e0001349. doi: 10.1371/journal.pgph.0001349 (PMC10022322; doi:10.1371/journal.pgph.0001349)
Supplement: S4 File — (PPT) [file pgph.0001349.s004.ppt]

## Slide 1
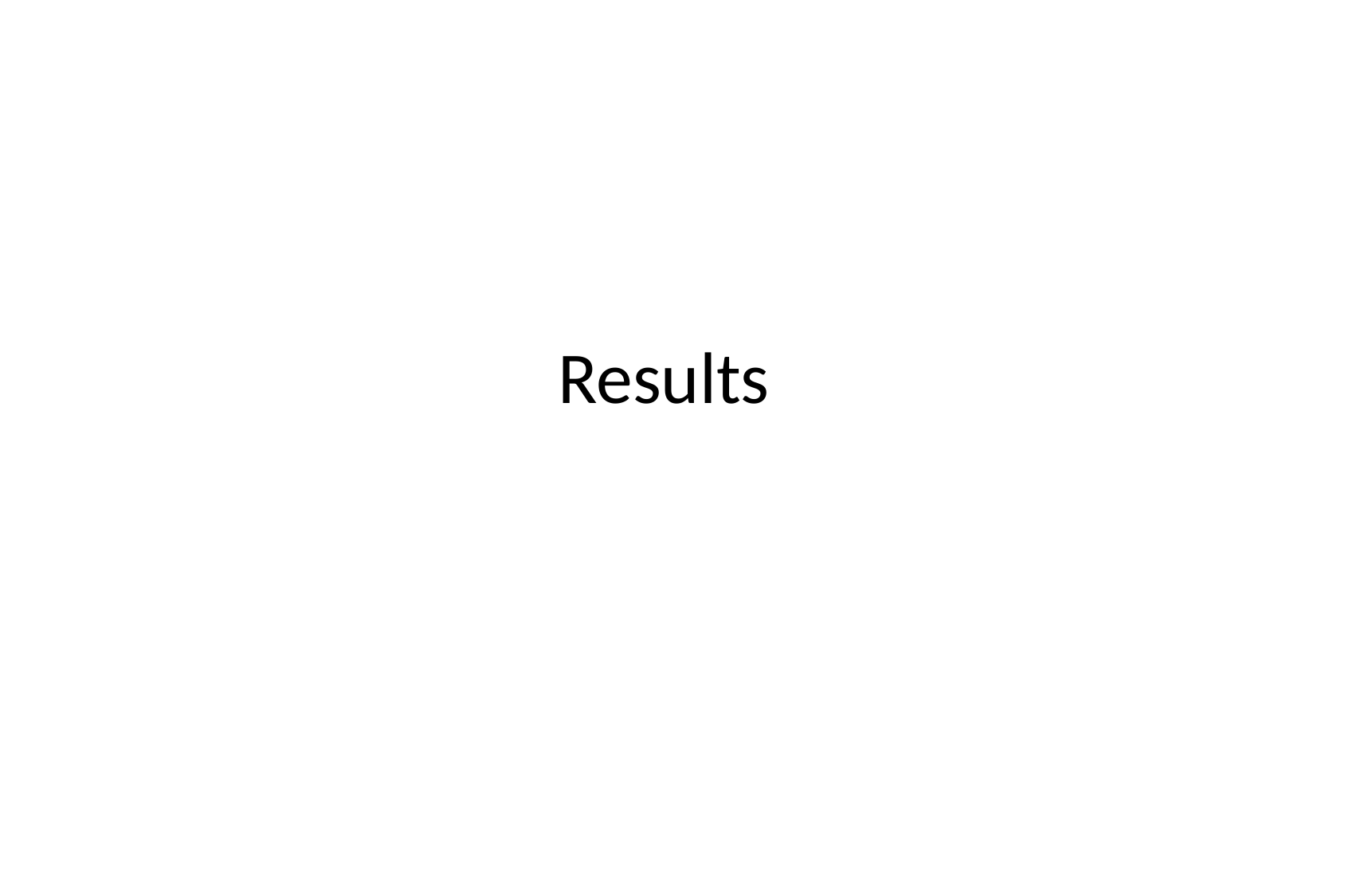

# Results

## Slide 2
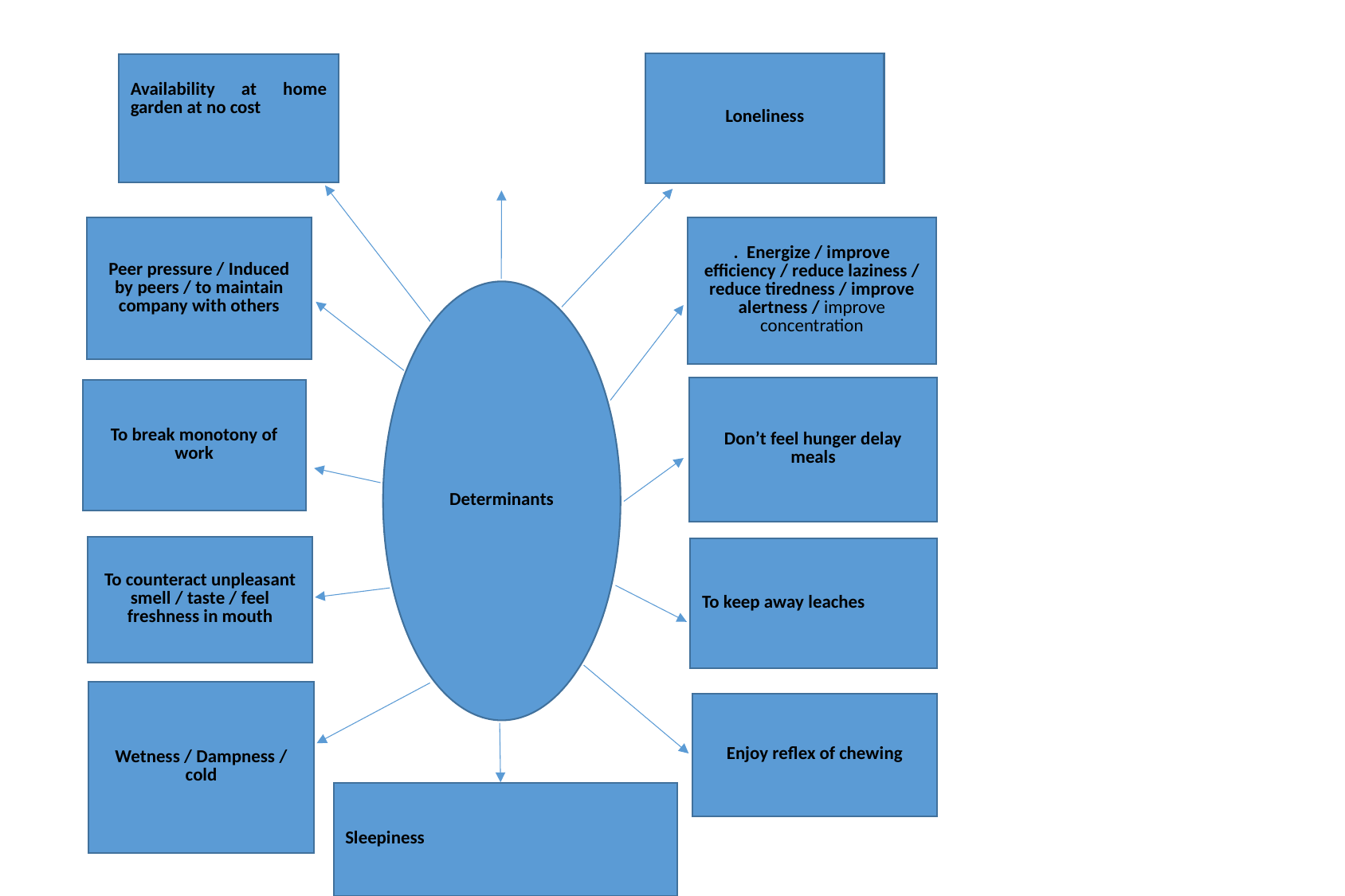

Loneliness
Availability at home garden at no cost
Peer pressure / Induced by peers / to maintain company with others
. Energize / improve efficiency / reduce laziness / reduce tiredness / improve alertness / improve concentration
Determinants
Don’t feel hunger delay meals
To break monotony of work
To counteract unpleasant smell / taste / feel freshness in mouth
To keep away leaches
Wetness / Dampness / cold
Enjoy reflex of chewing
Sleepiness

## Slide 3
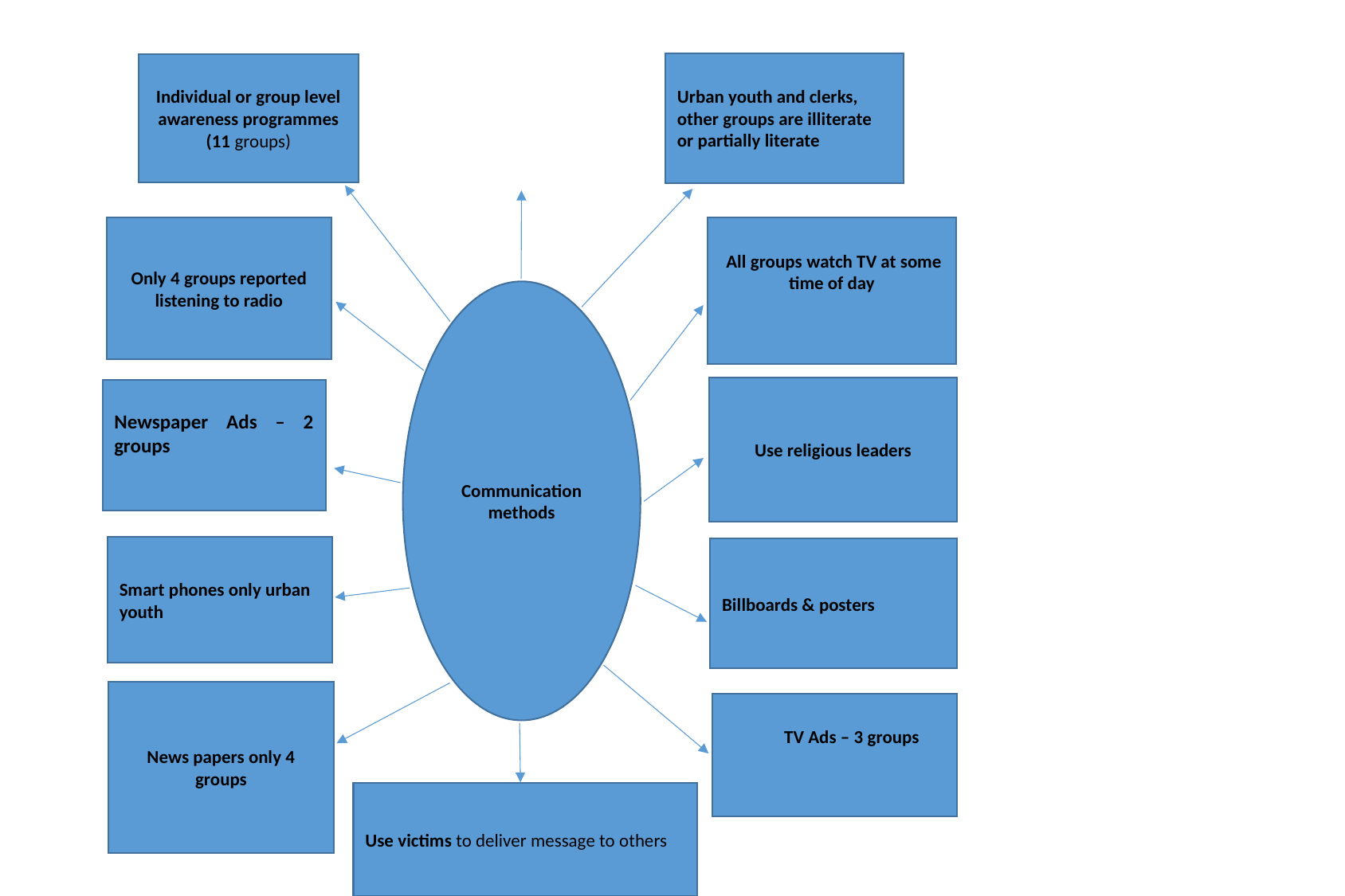

Urban youth and clerks, other groups are illiterate or partially literate
Individual or group level awareness programmes (11 groups)
Only 4 groups reported listening to radio
 All groups watch TV at some time of day
Communication methods
Use religious leaders
Newspaper Ads – 2 groups
Smart phones only urban youth
Billboards & posters
News papers only 4 groups
TV Ads – 3 groups
Use victims to deliver message to others

## Slide 4
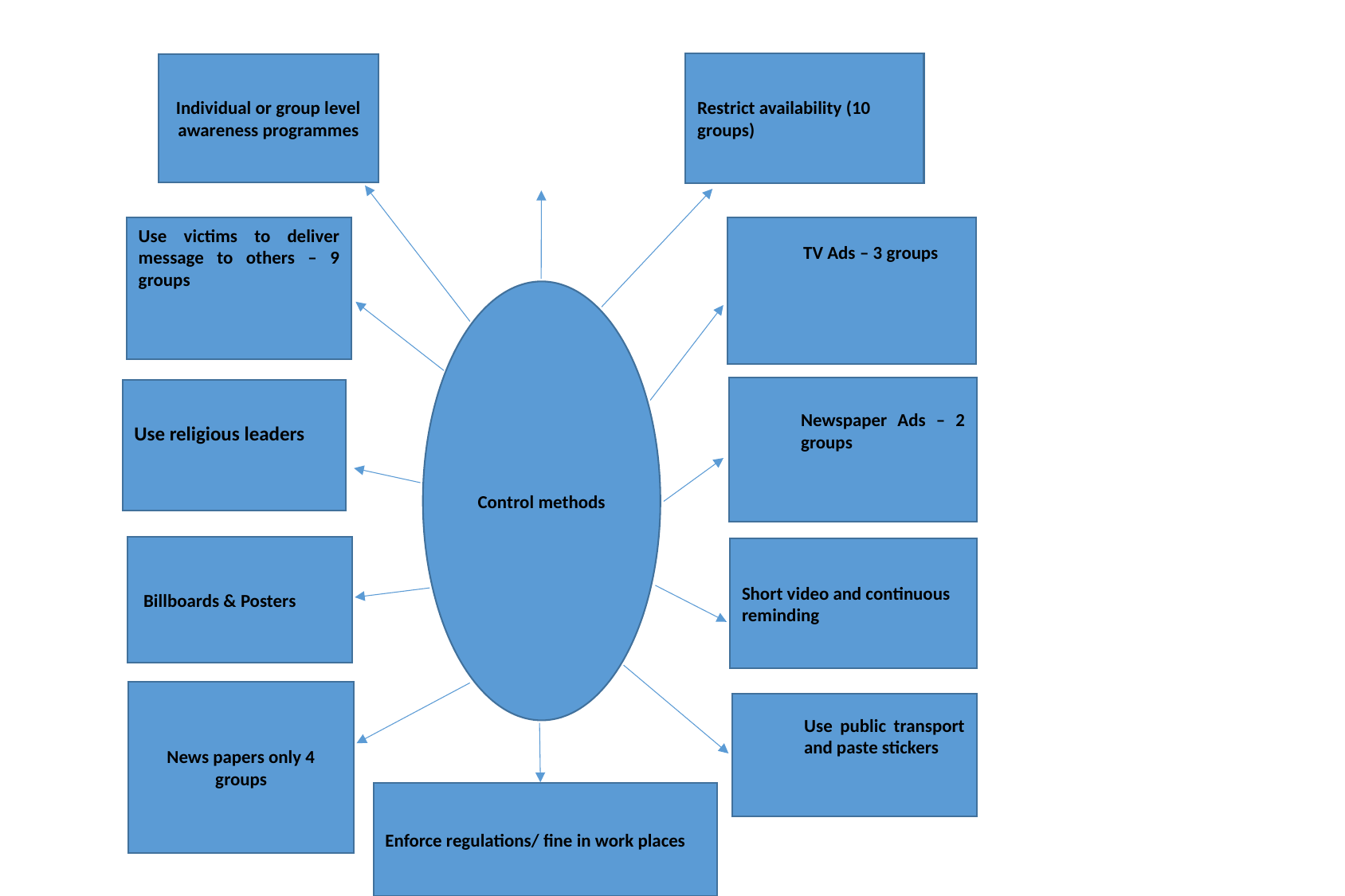

Restrict availability (10 groups)
Individual or group level awareness programmes
Use victims to deliver message to others – 9 groups
 TV Ads – 3 groups
Control methods
Newspaper Ads – 2 groups
Use religious leaders
 Billboards & Posters
Short video and continuous reminding
News papers only 4 groups
Use public transport and paste stickers
Enforce regulations/ fine in work places

## Slide 5
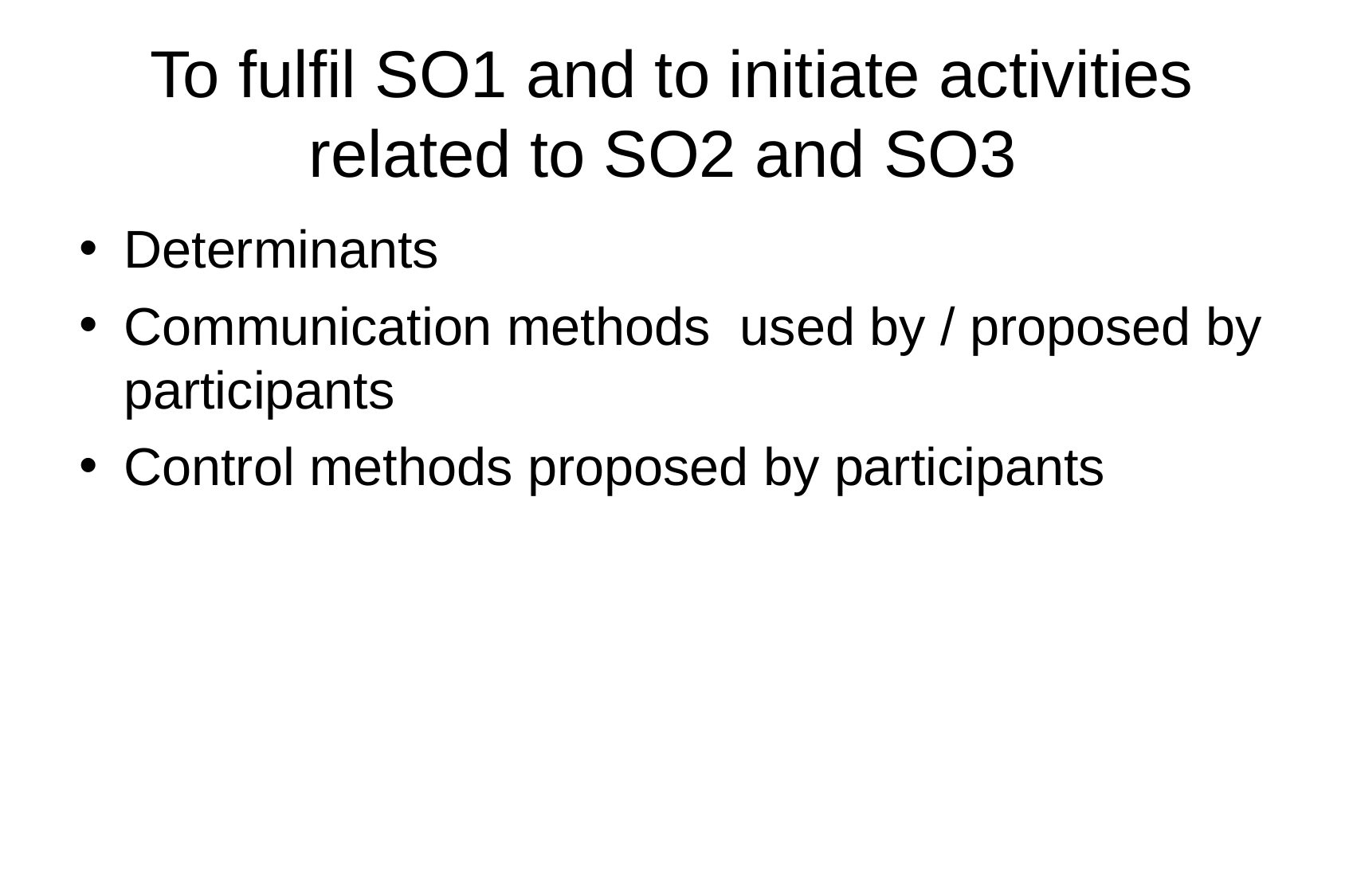

# To fulfil SO1 and to initiate activities related to SO2 and SO3
Determinants
Communication methods used by / proposed by participants
Control methods proposed by participants

## Slide 6
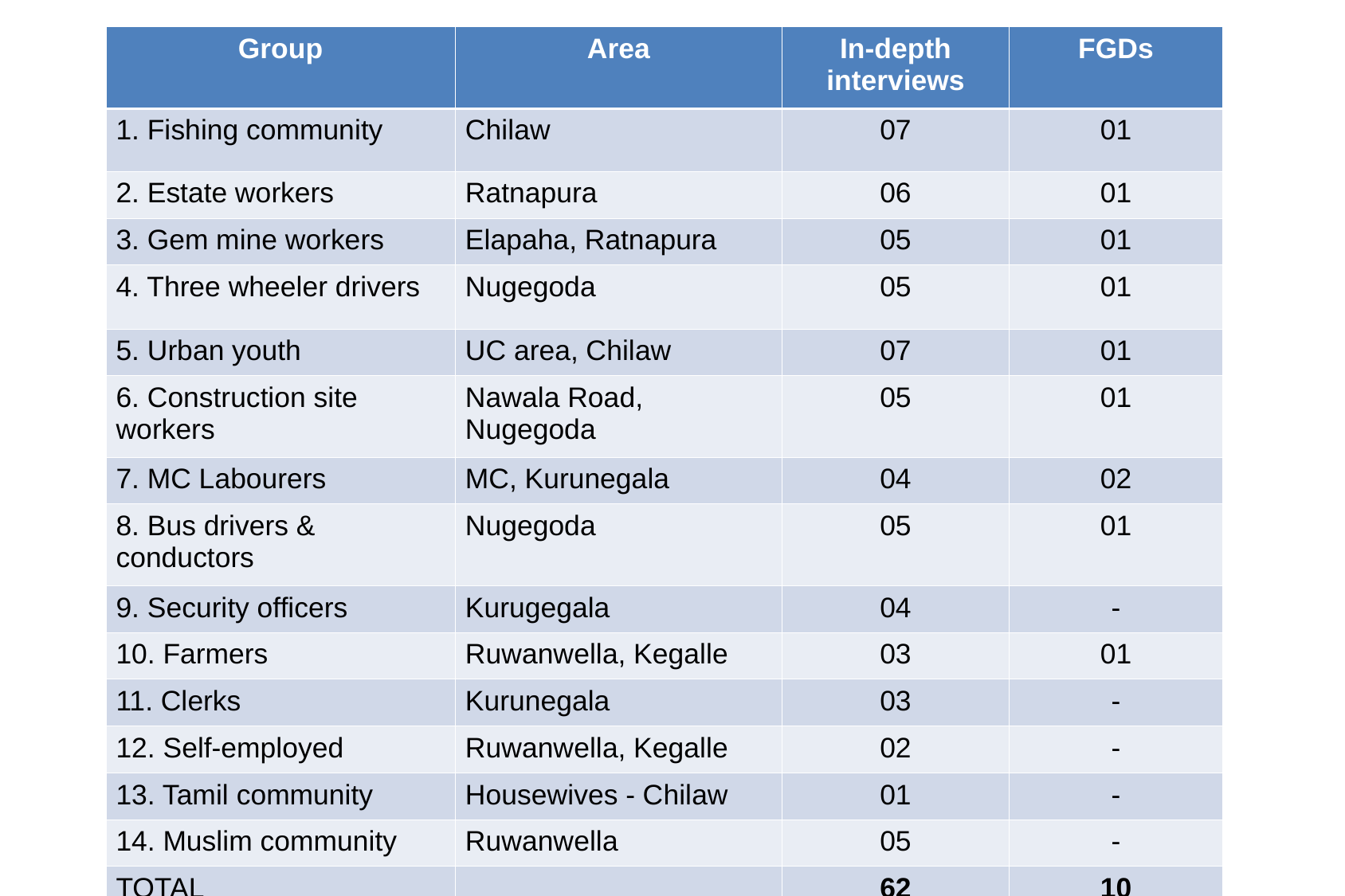

| Group | Area | In-depth interviews | FGDs |
| --- | --- | --- | --- |
| 1. Fishing community | Chilaw | 07 | 01 |
| 2. Estate workers | Ratnapura | 06 | 01 |
| 3. Gem mine workers | Elapaha, Ratnapura | 05 | 01 |
| 4. Three wheeler drivers | Nugegoda | 05 | 01 |
| 5. Urban youth | UC area, Chilaw | 07 | 01 |
| 6. Construction site workers | Nawala Road, Nugegoda | 05 | 01 |
| 7. MC Labourers | MC, Kurunegala | 04 | 02 |
| 8. Bus drivers & conductors | Nugegoda | 05 | 01 |
| 9. Security officers | Kurugegala | 04 | - |
| 10. Farmers | Ruwanwella, Kegalle | 03 | 01 |
| 11. Clerks | Kurunegala | 03 | - |
| 12. Self-employed | Ruwanwella, Kegalle | 02 | - |
| 13. Tamil community | Housewives - Chilaw | 01 | - |
| 14. Muslim community | Ruwanwella | 05 | - |
| TOTAL | | 62 | 10 |
#

## Slide 7
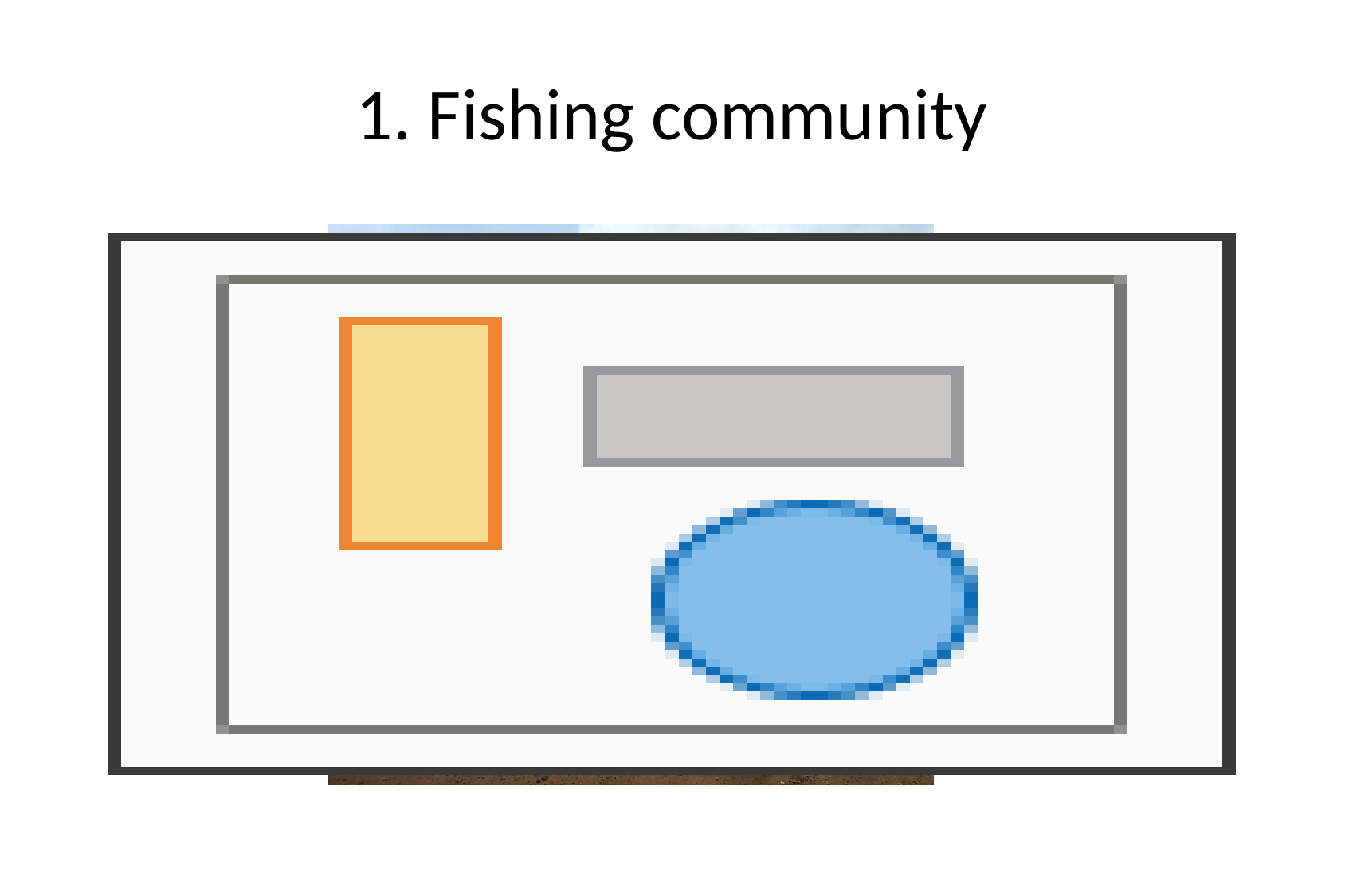

# 1. Fishing community

## Slide 8
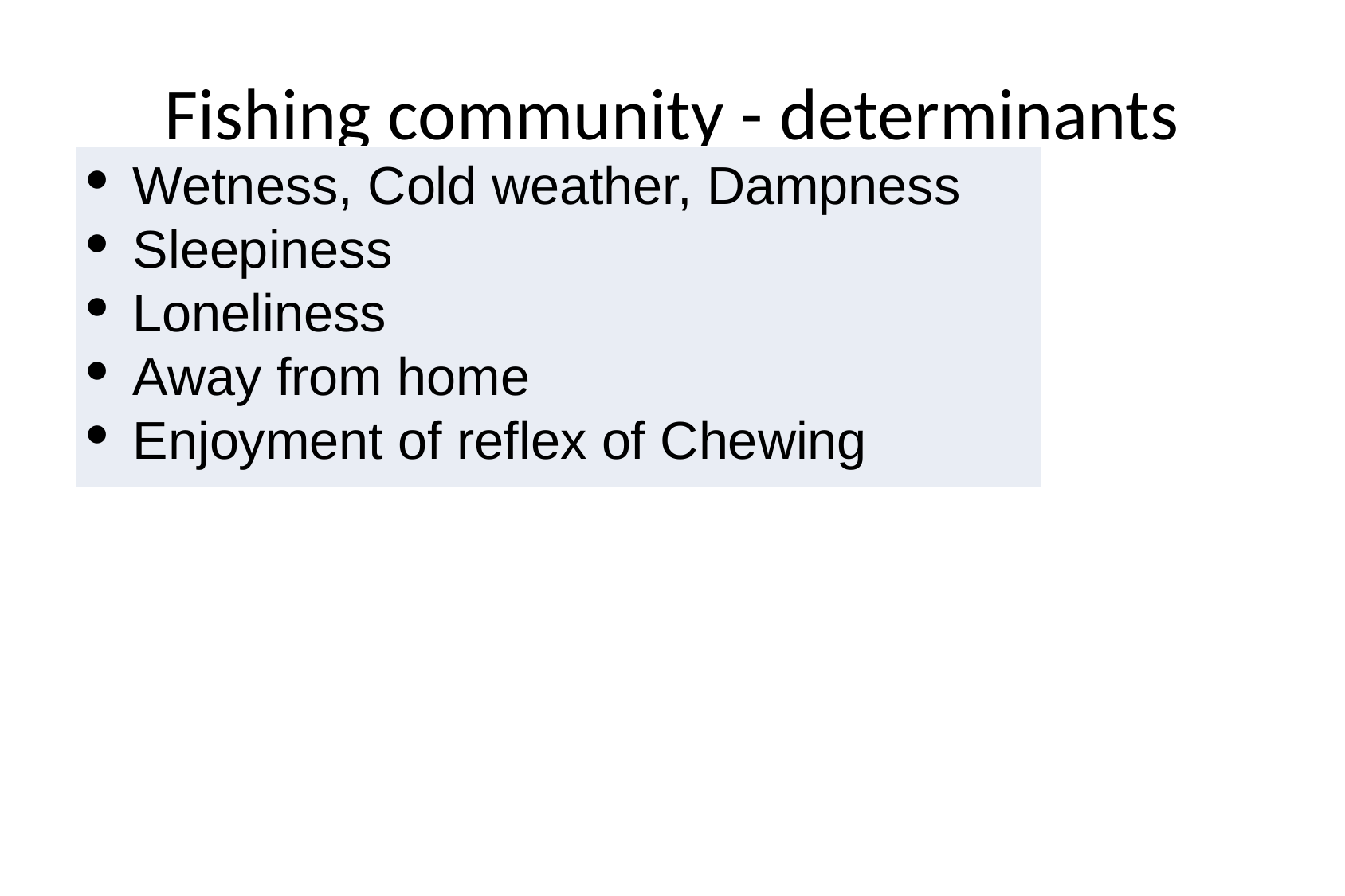

# Fishing community - determinants
| Wetness, Cold weather, Dampness Sleepiness Loneliness Away from home Enjoyment of reflex of Chewing |
| --- |

## Slide 9
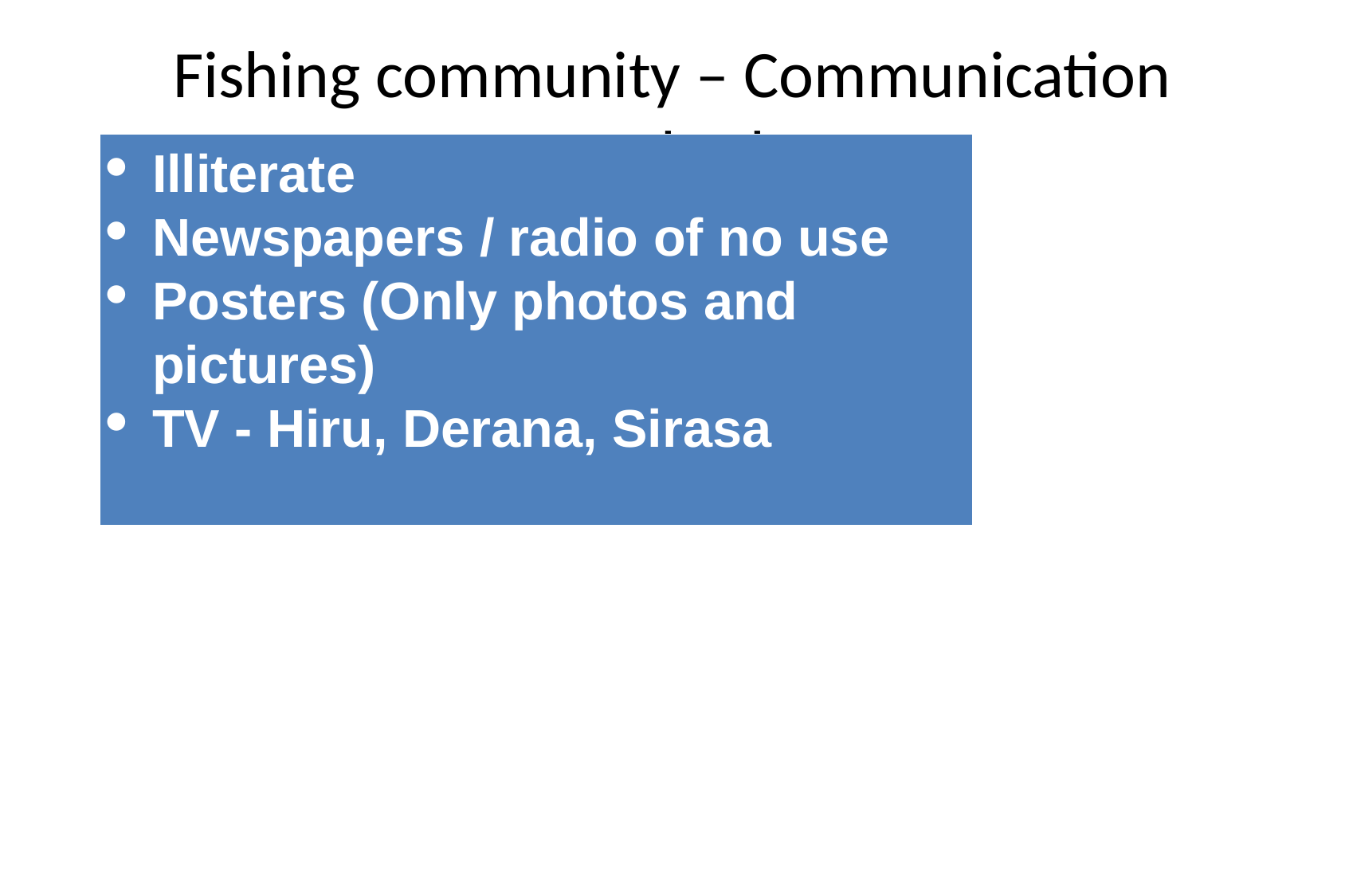

# Fishing community – Communication methods
| Illiterate Newspapers / radio of no use Posters (Only photos and pictures) TV - Hiru, Derana, Sirasa |
| --- |

## Slide 10
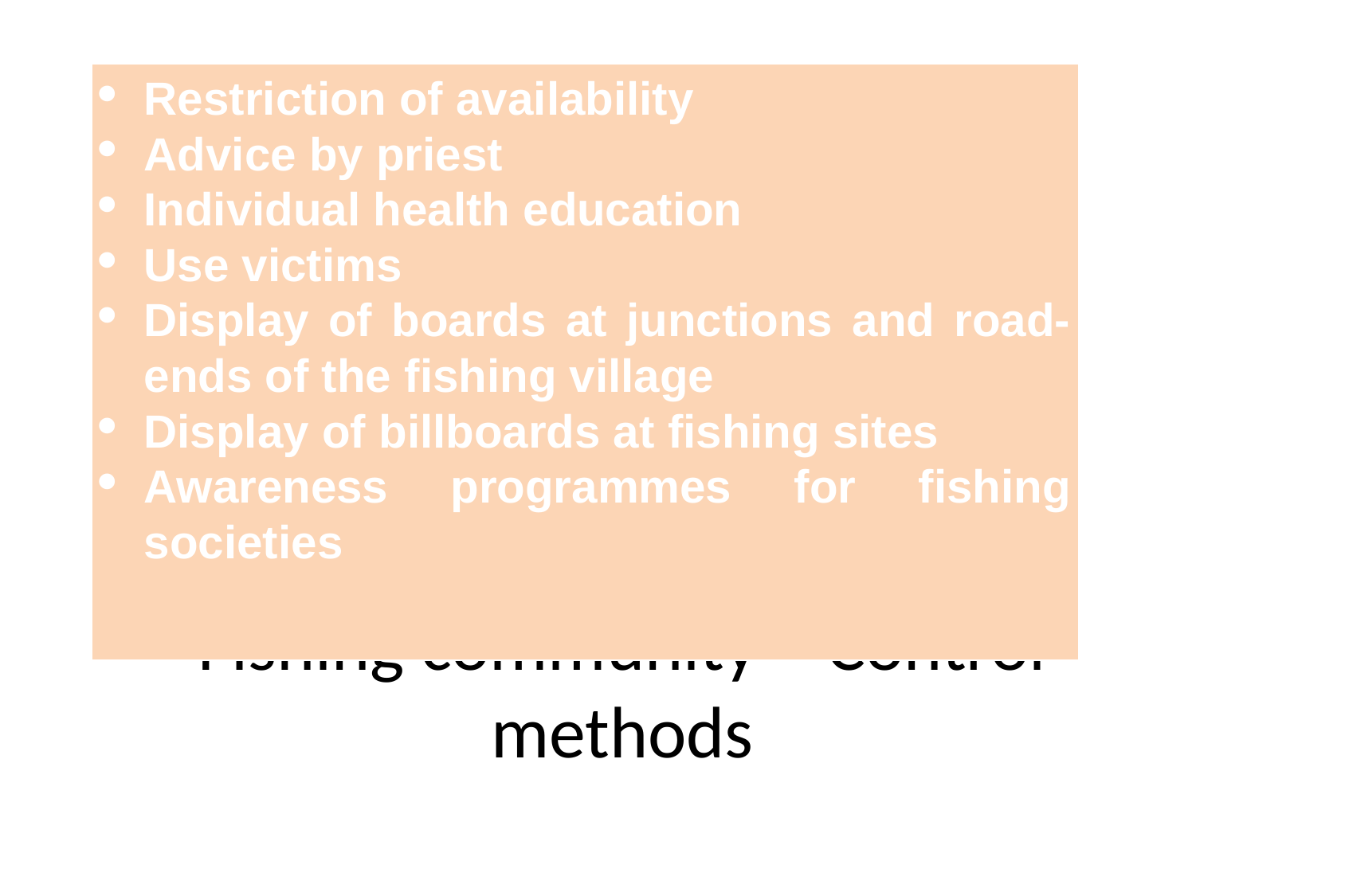

| Restriction of availability Advice by priest Individual health education Use victims Display of boards at junctions and road-ends of the fishing village Display of billboards at fishing sites Awareness programmes for fishing societies |
| --- |
# Fishing community – Control methods

## Slide 11
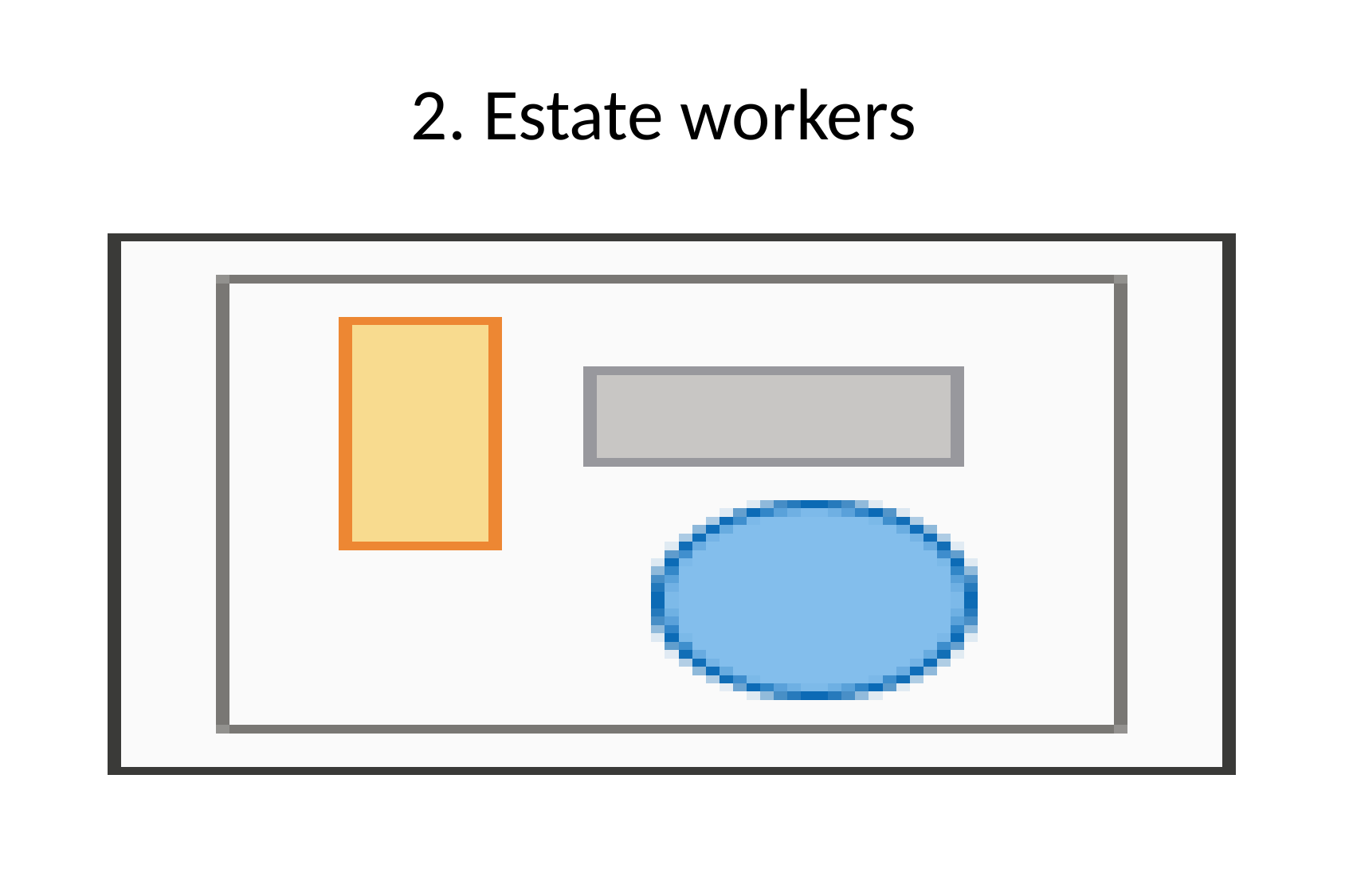

# 2. Estate workers

## Slide 12
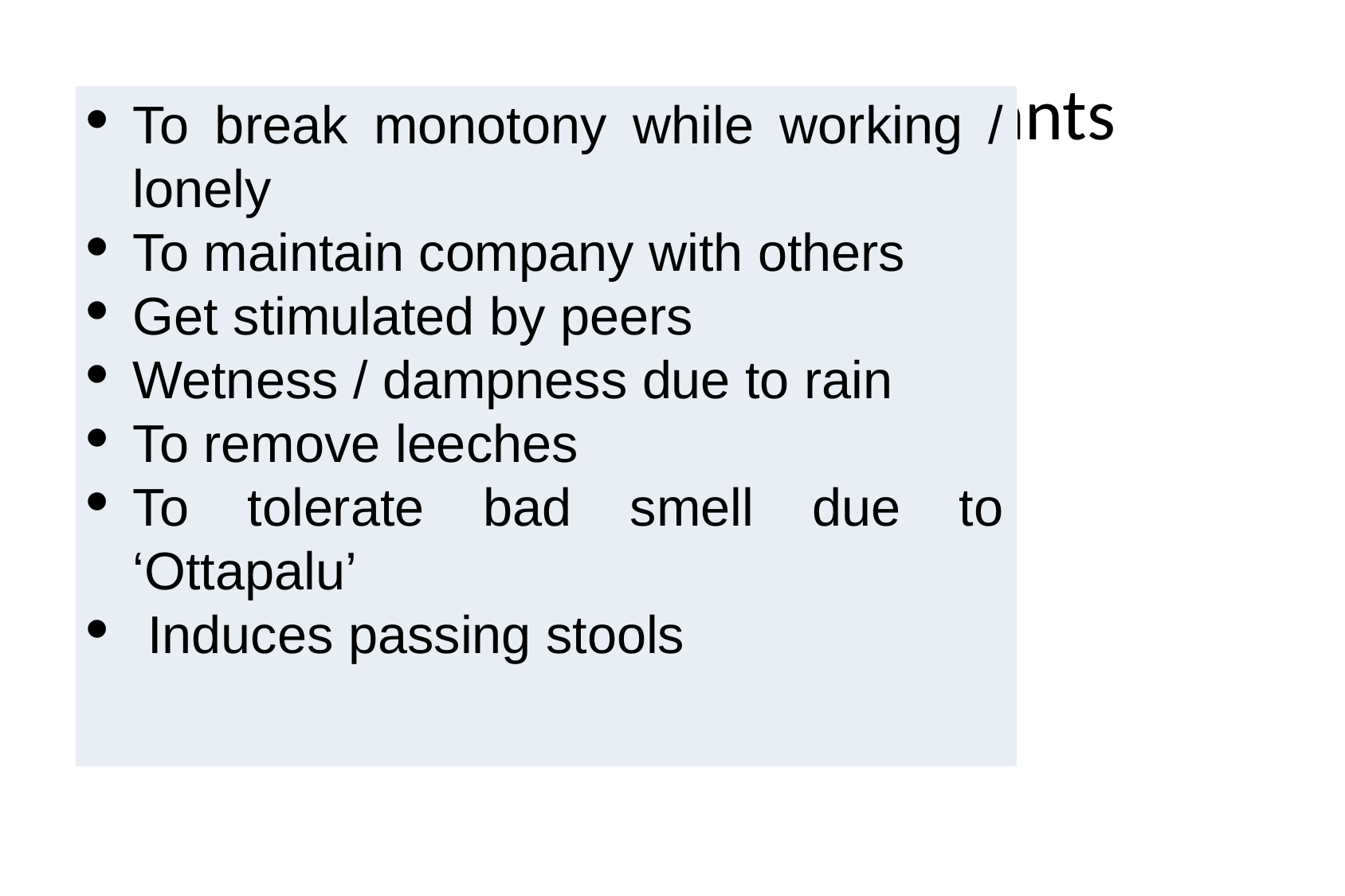

# Estate workers - determinants
| To break monotony while working / lonely To maintain company with others Get stimulated by peers Wetness / dampness due to rain To remove leeches To tolerate bad smell due to ‘Ottapalu’ Induces passing stools |
| --- |

## Slide 13
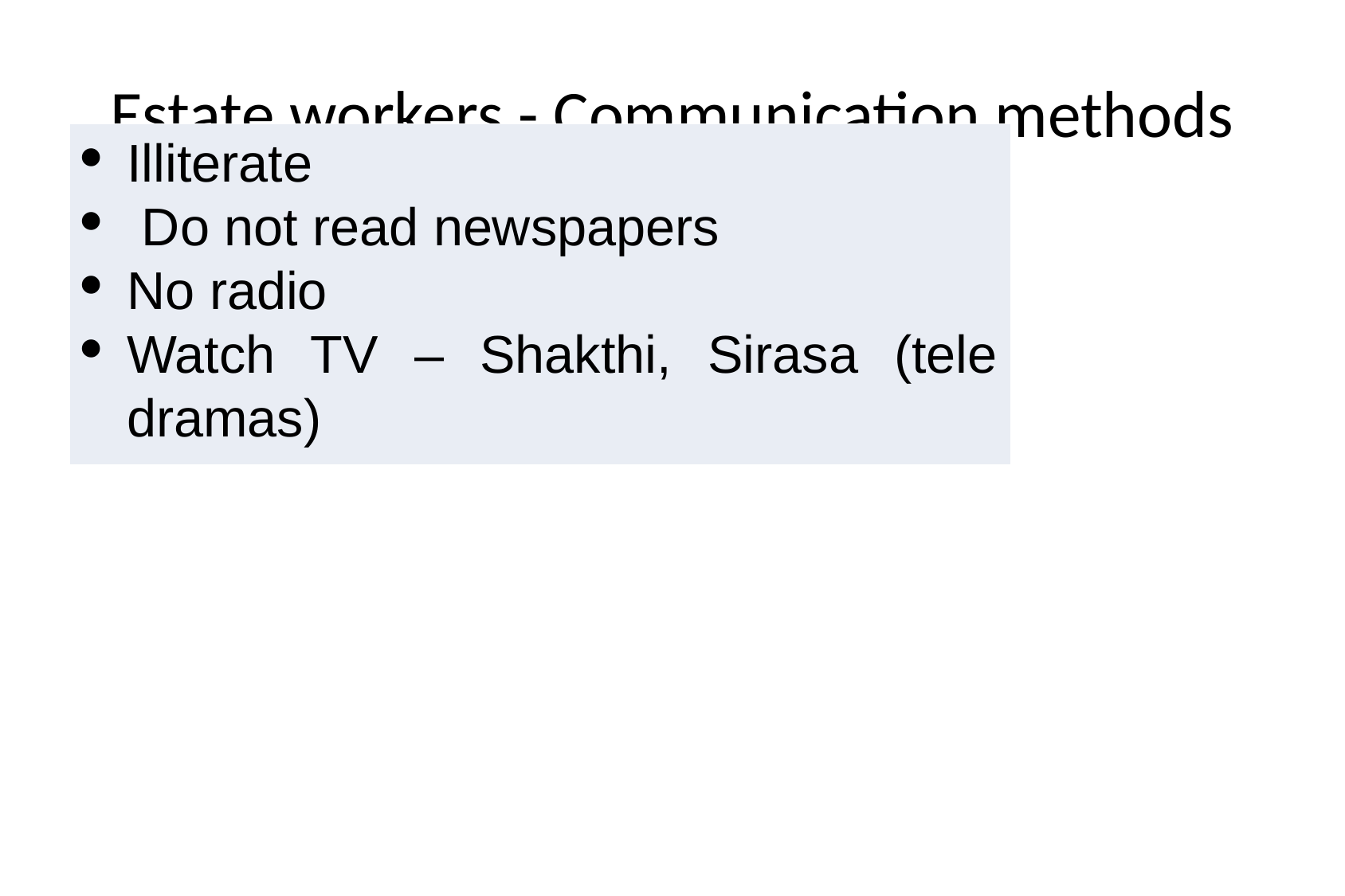

# Estate workers - Communication methods
| Illiterate Do not read newspapers No radio Watch TV – Shakthi, Sirasa (tele dramas) |
| --- |

## Slide 14
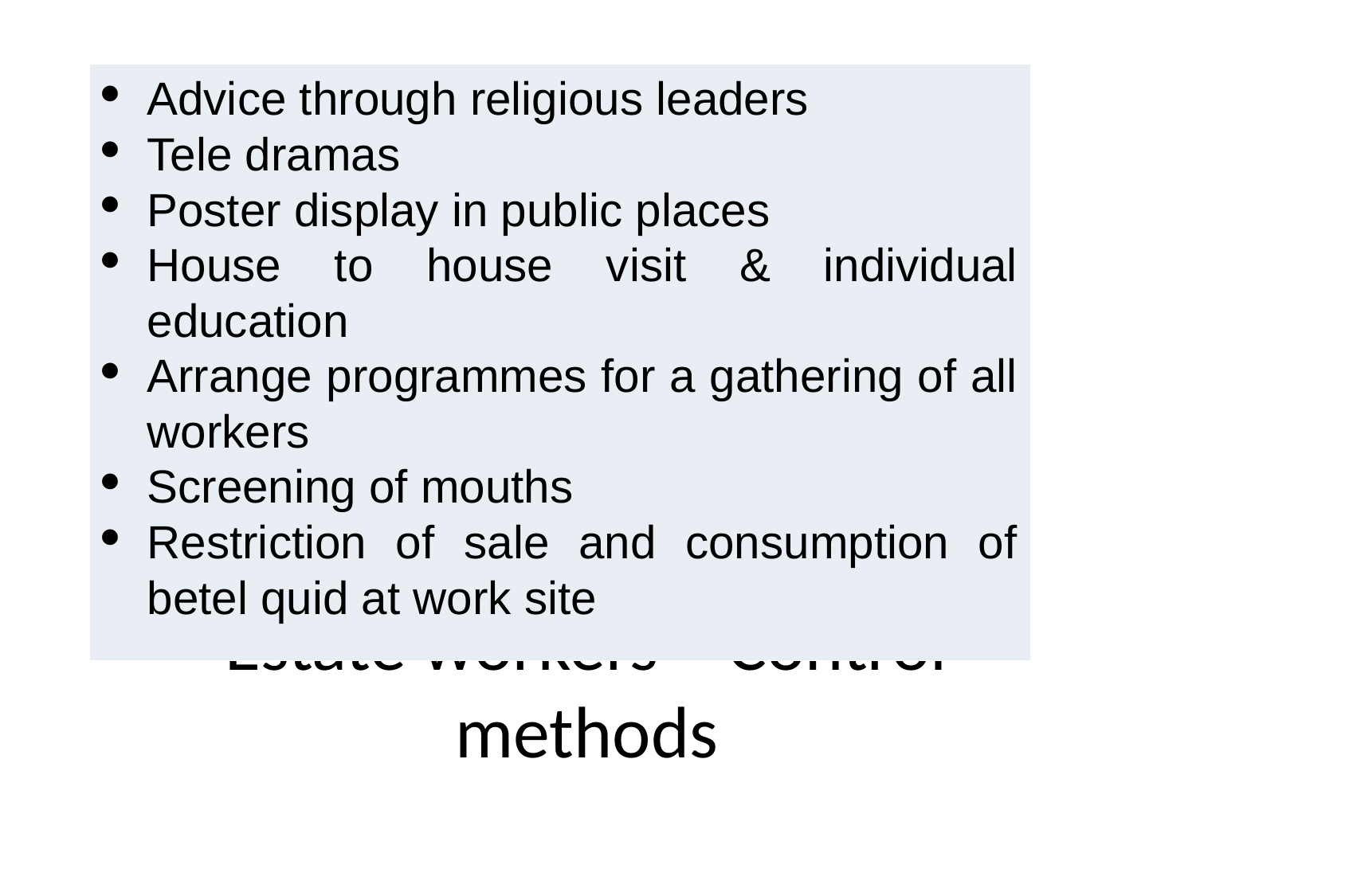

| Advice through religious leaders Tele dramas Poster display in public places House to house visit & individual education Arrange programmes for a gathering of all workers Screening of mouths Restriction of sale and consumption of betel quid at work site |
| --- |
# Estate workers – Control methods

## Slide 15
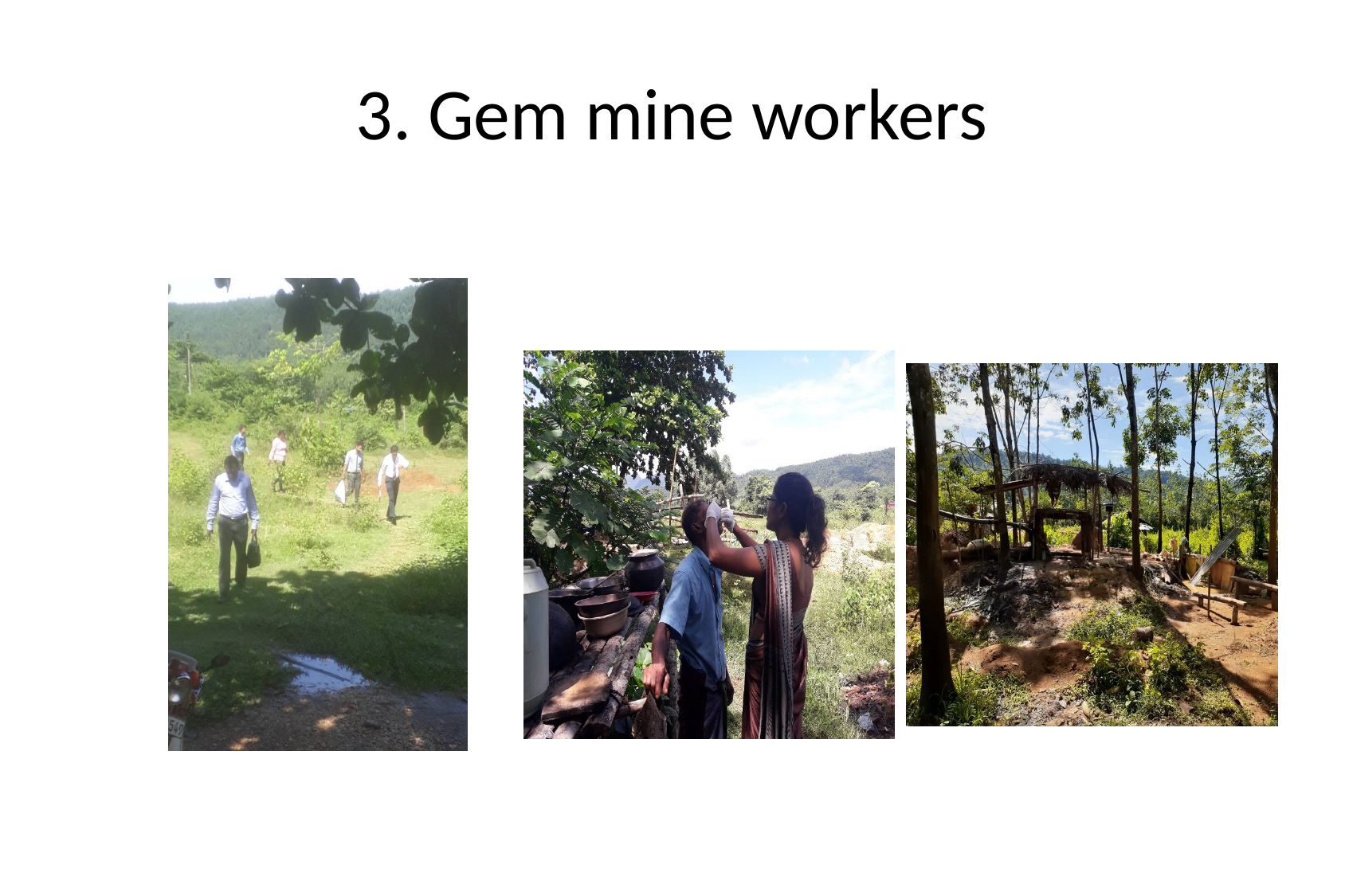

# 3. Gem mine workers

## Slide 16
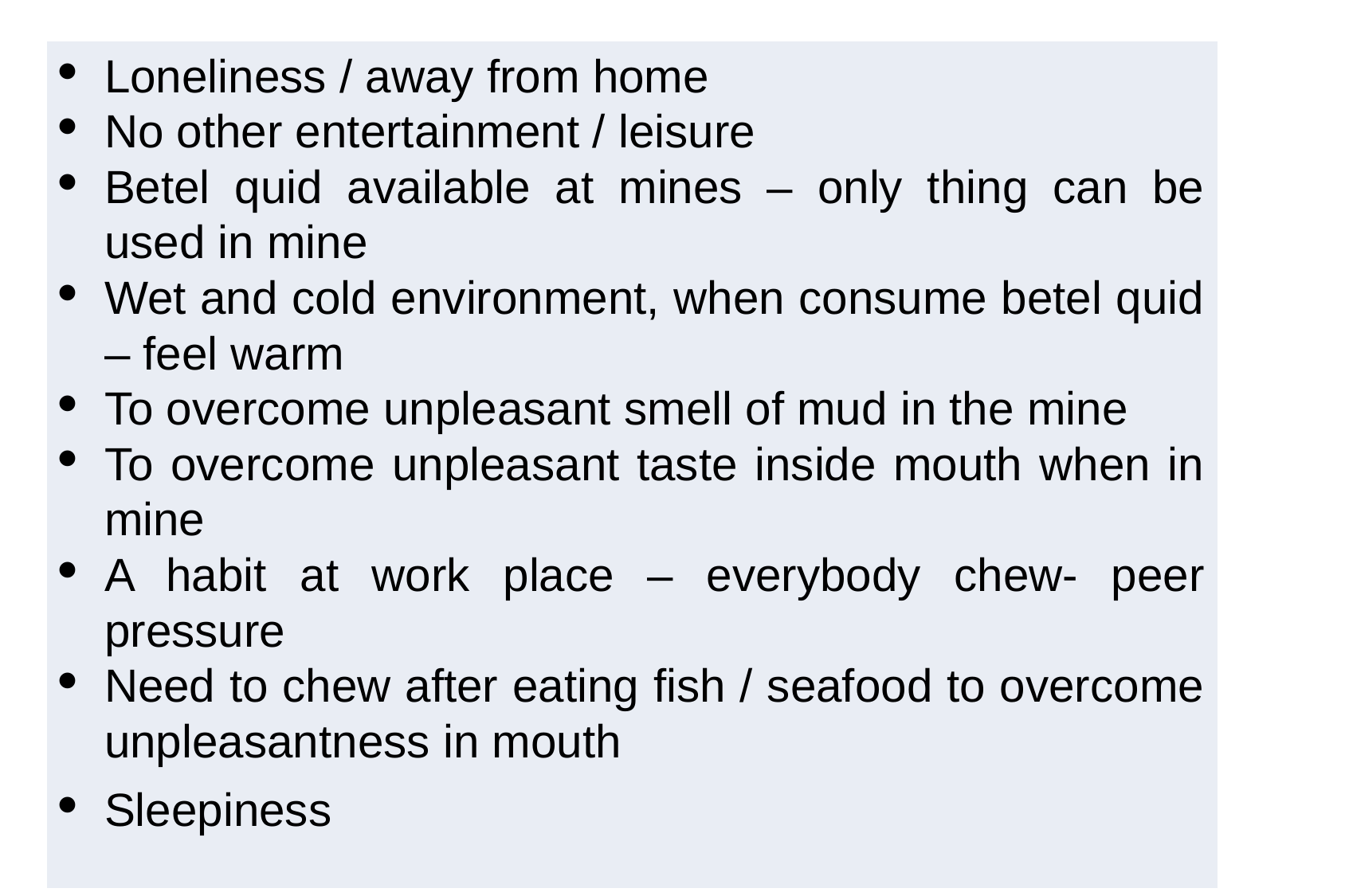

| Loneliness / away from home No other entertainment / leisure Betel quid available at mines – only thing can be used in mine Wet and cold environment, when consume betel quid – feel warm To overcome unpleasant smell of mud in the mine To overcome unpleasant taste inside mouth when in mine A habit at work place – everybody chew- peer pressure Need to chew after eating fish / seafood to overcome unpleasantness in mouth Sleepiness |
| --- |
# Gem mine workers - determinants

## Slide 17
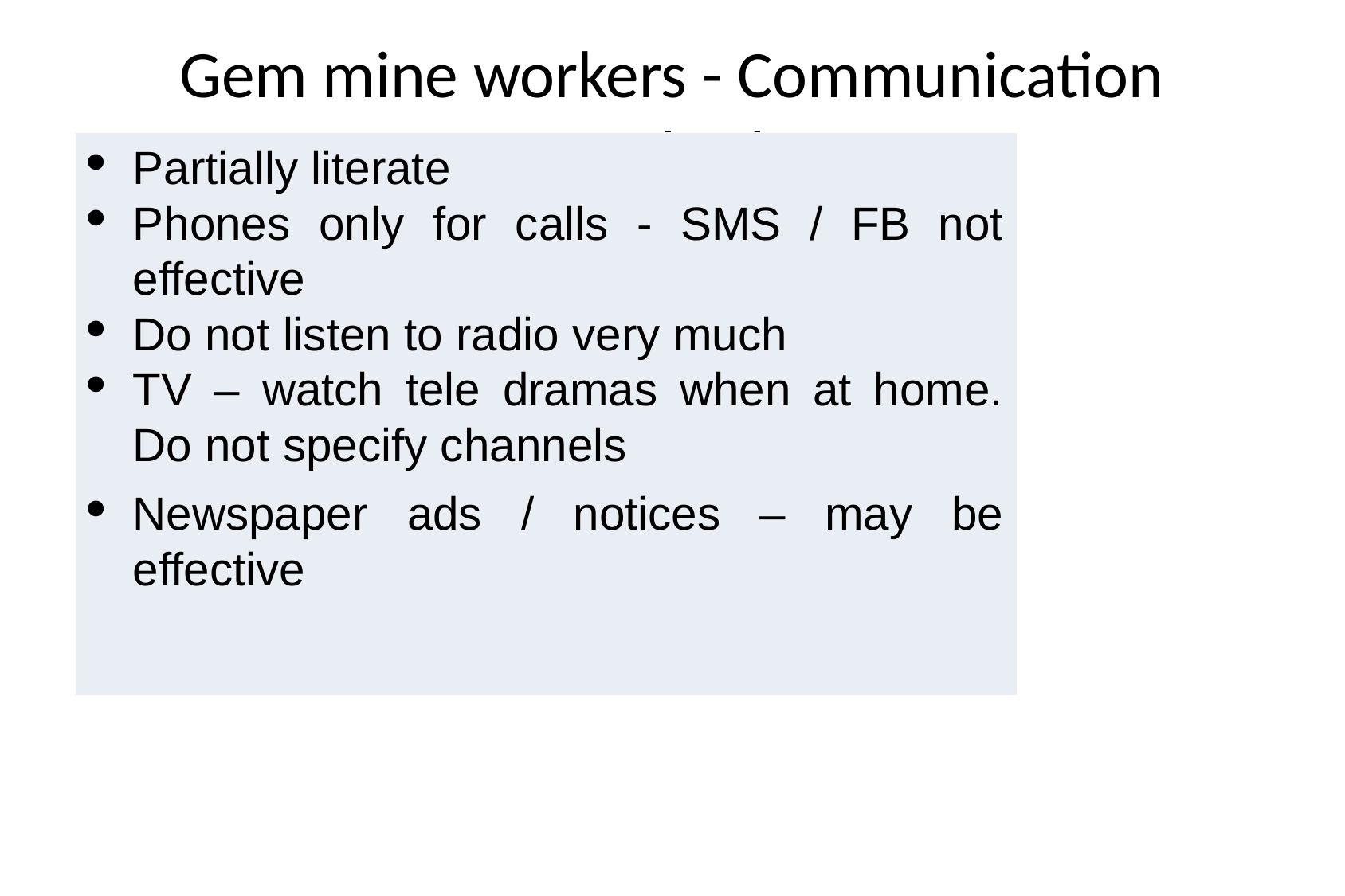

# Gem mine workers - Communication methods
| Partially literate Phones only for calls - SMS / FB not effective Do not listen to radio very much TV – watch tele dramas when at home. Do not specify channels Newspaper ads / notices – may be effective |
| --- |

## Slide 18
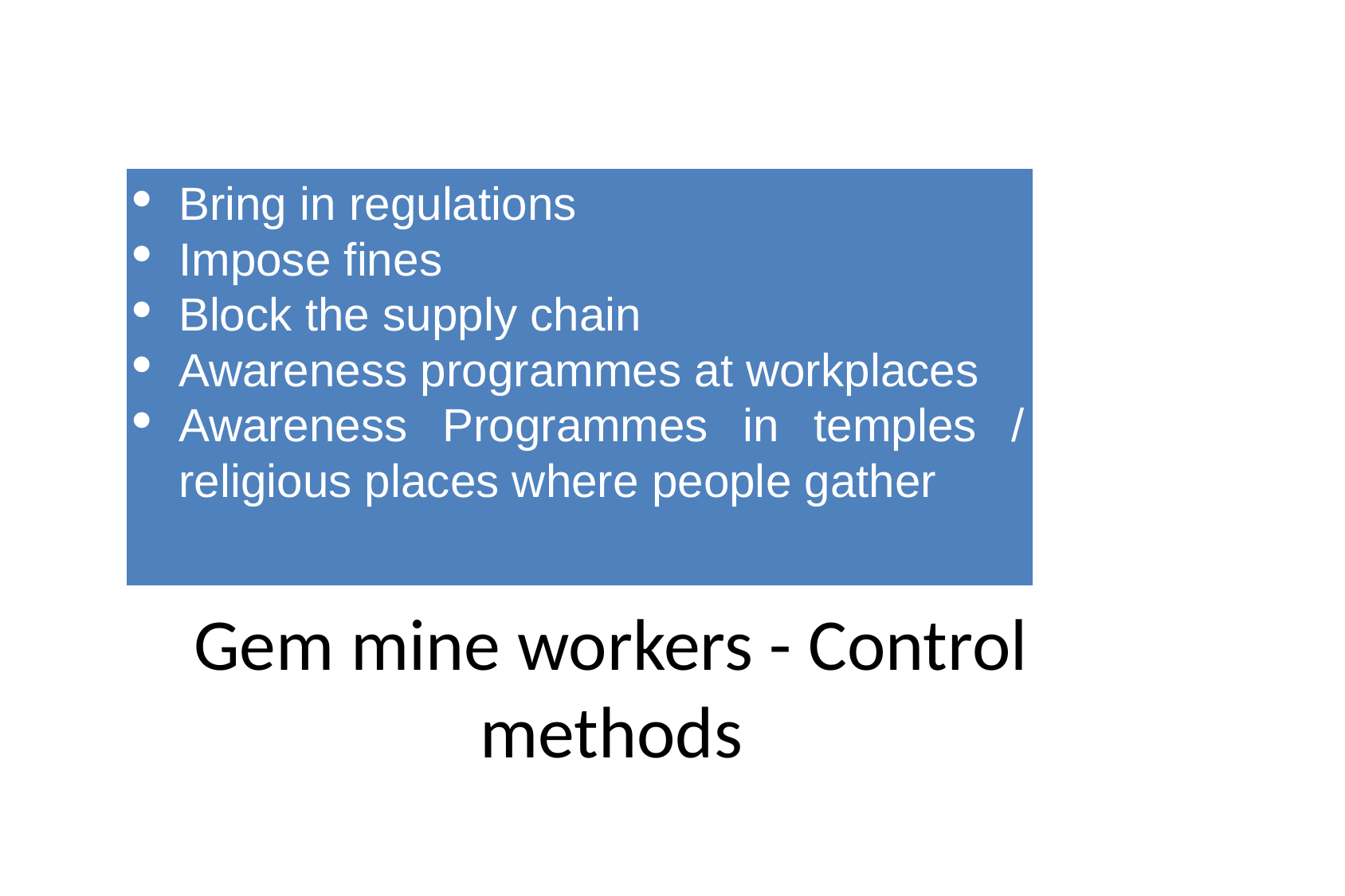

| Bring in regulations Impose fines Block the supply chain Awareness programmes at workplaces Awareness Programmes in temples / religious places where people gather |
| --- |
# Gem mine workers - Control methods

## Slide 19
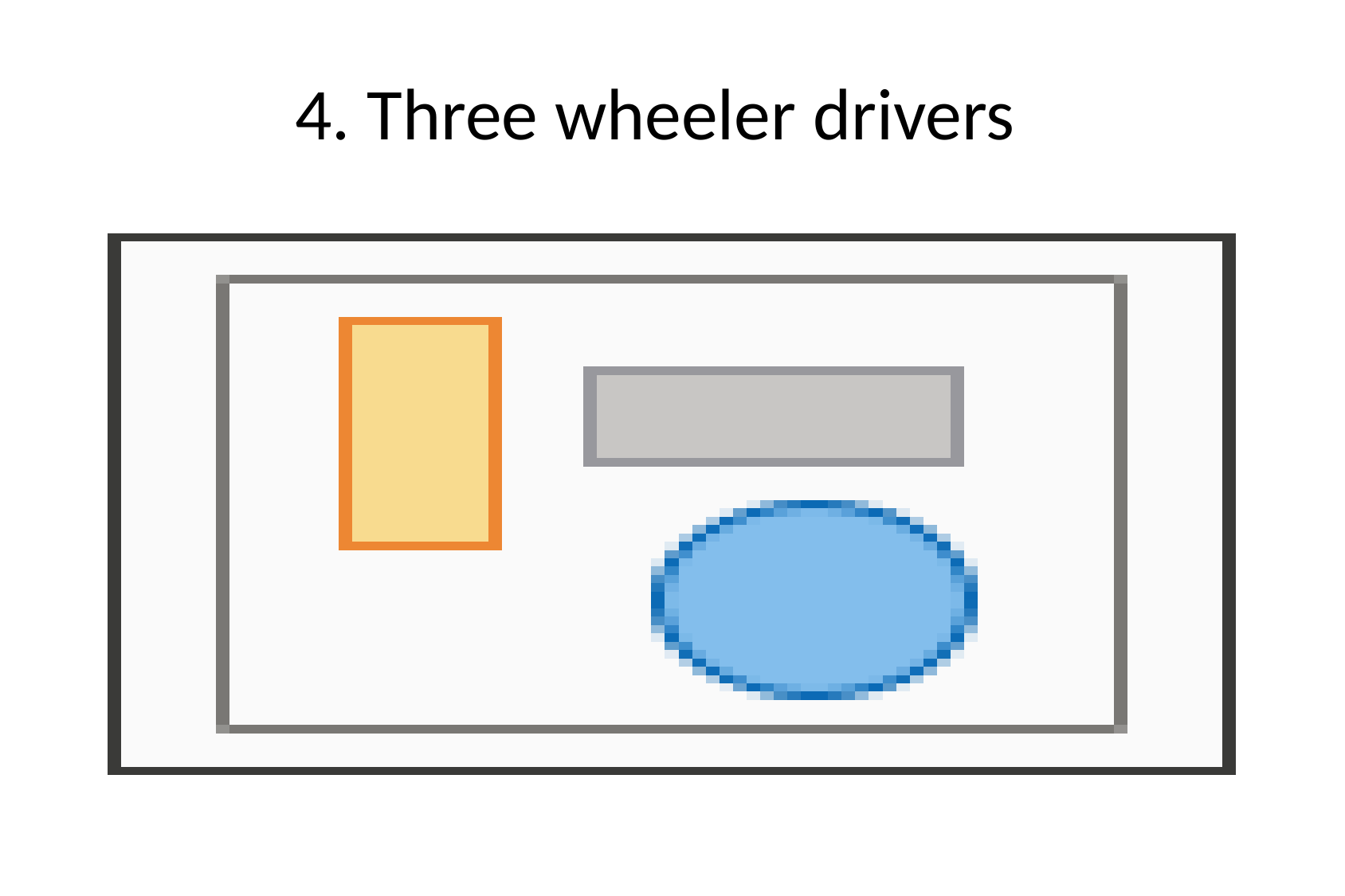

# 4. Three wheeler drivers

## Slide 20
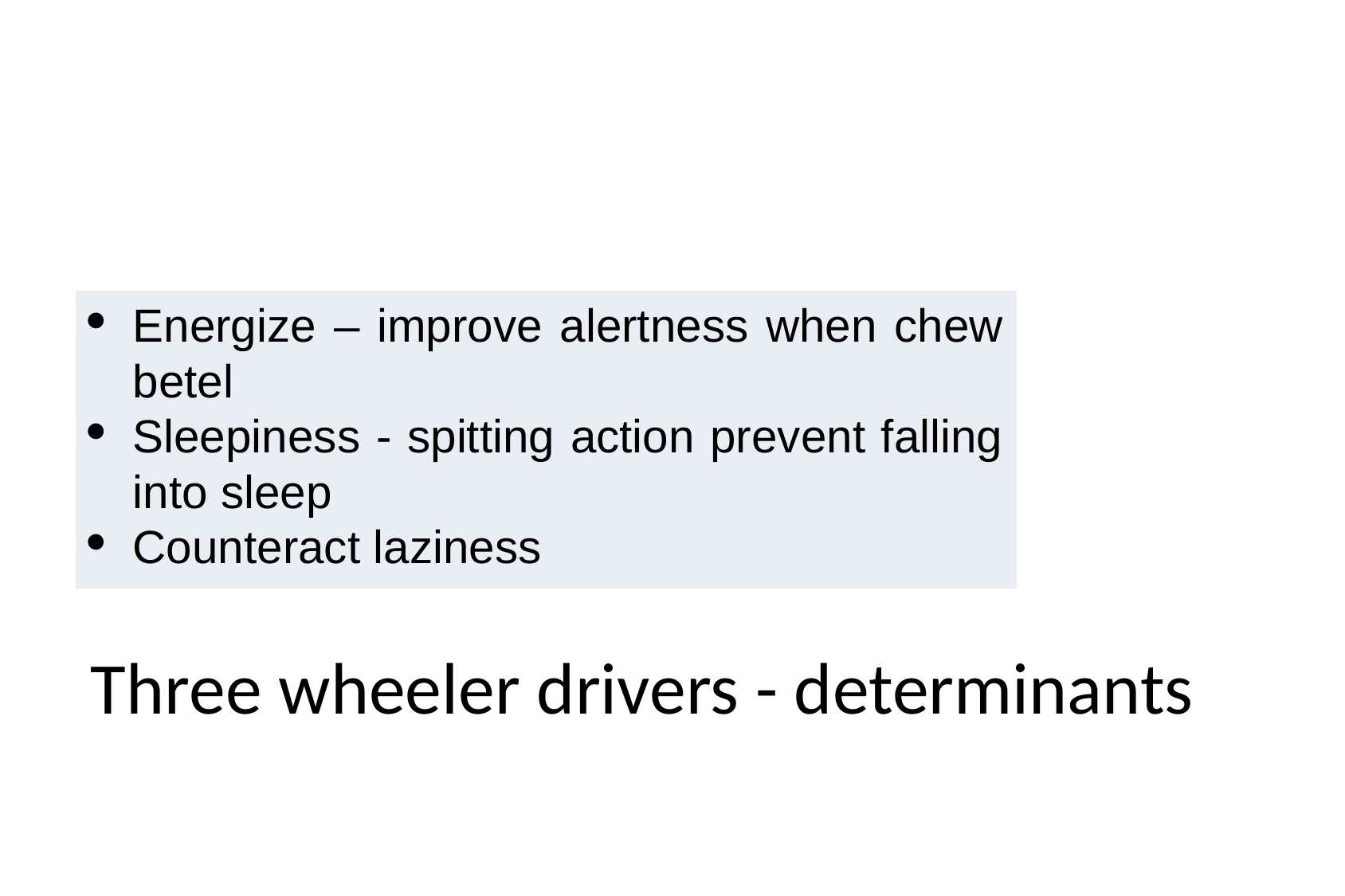

| Energize – improve alertness when chew betel Sleepiness - spitting action prevent falling into sleep Counteract laziness |
| --- |
# Three wheeler drivers - determinants

## Slide 21
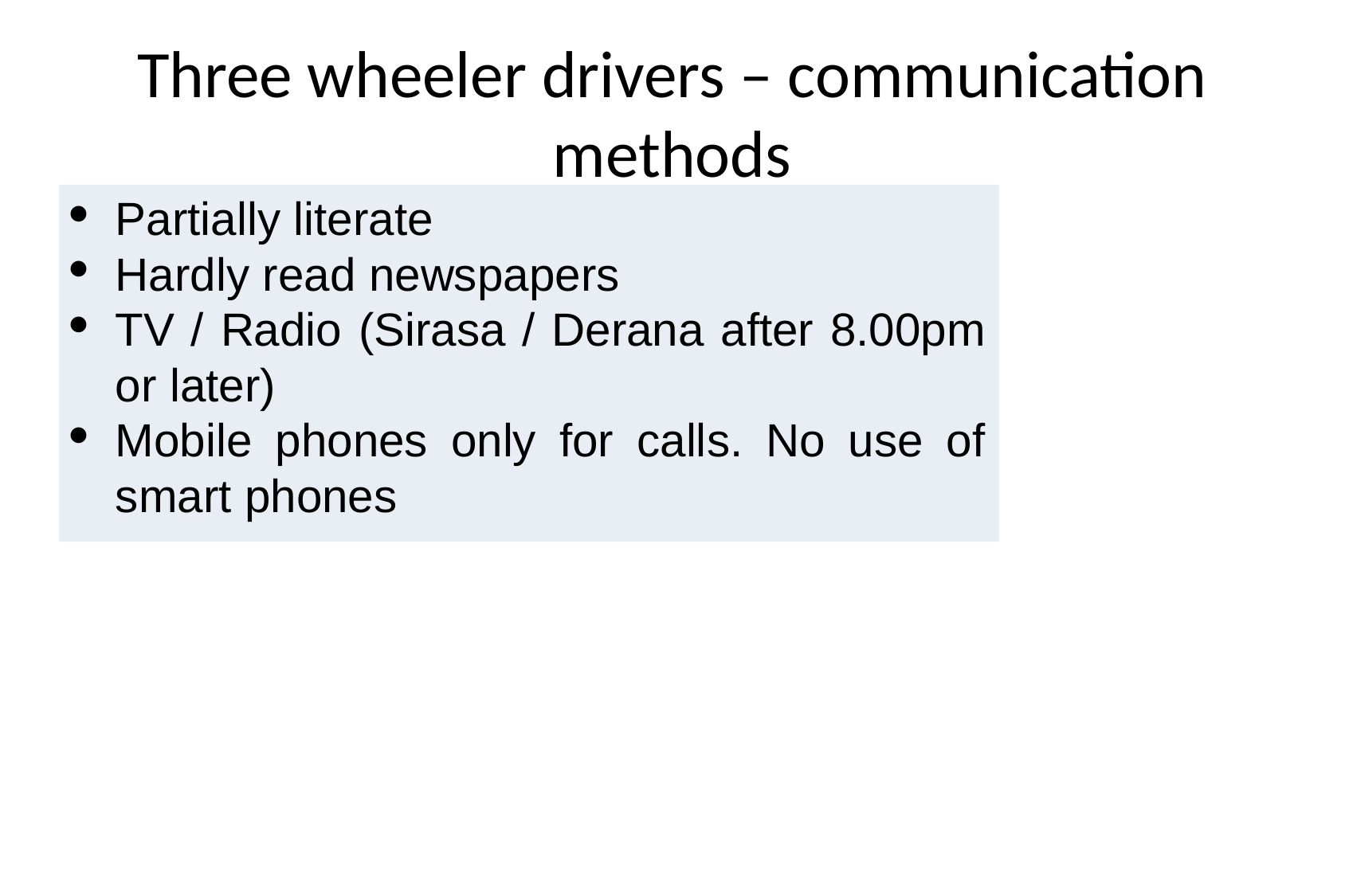

# Three wheeler drivers – communication methods
| Partially literate Hardly read newspapers TV / Radio (Sirasa / Derana after 8.00pm or later) Mobile phones only for calls. No use of smart phones |
| --- |

## Slide 22
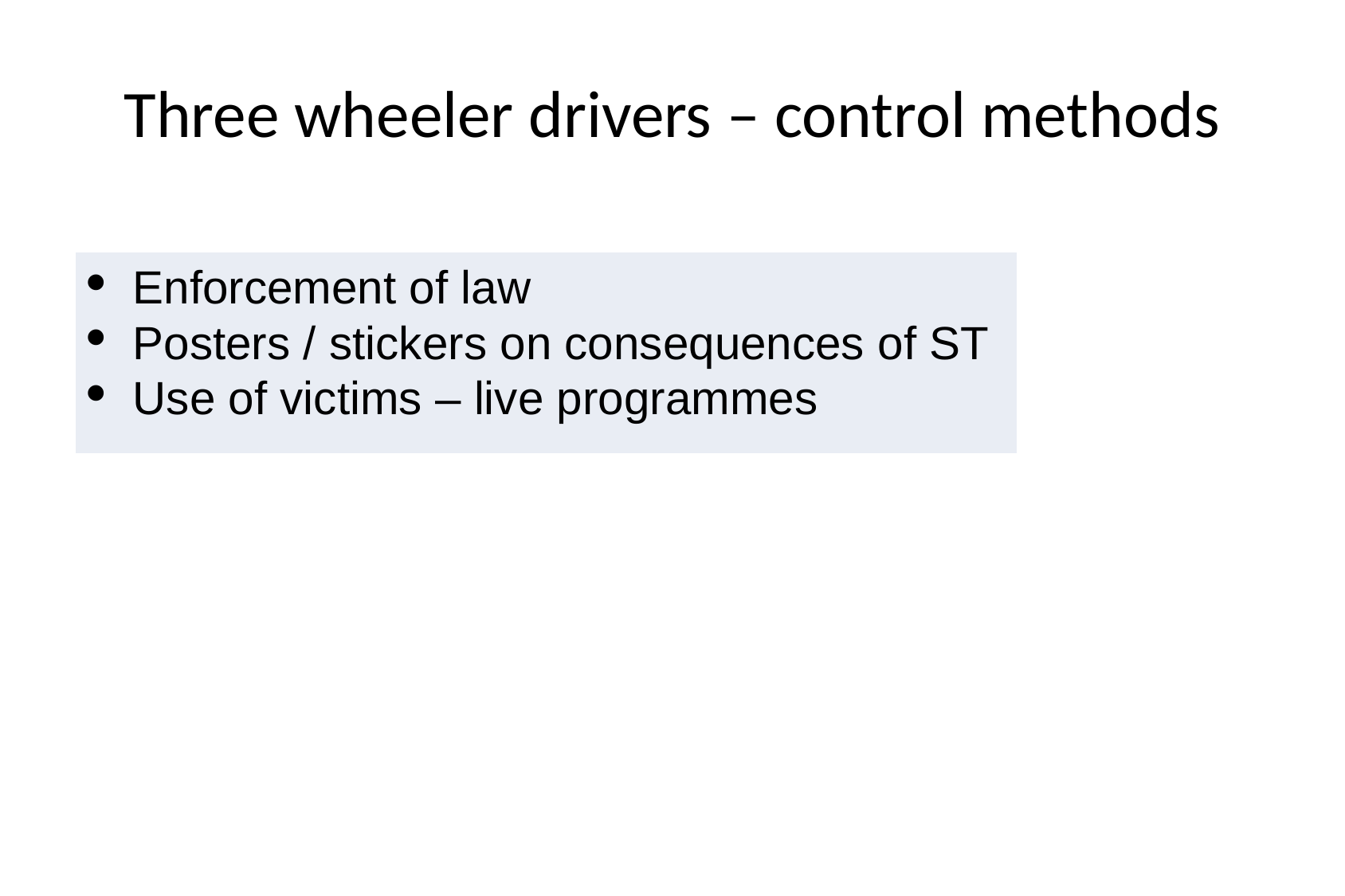

# Three wheeler drivers – control methods
| Enforcement of law Posters / stickers on consequences of ST Use of victims – live programmes |
| --- |

## Slide 23
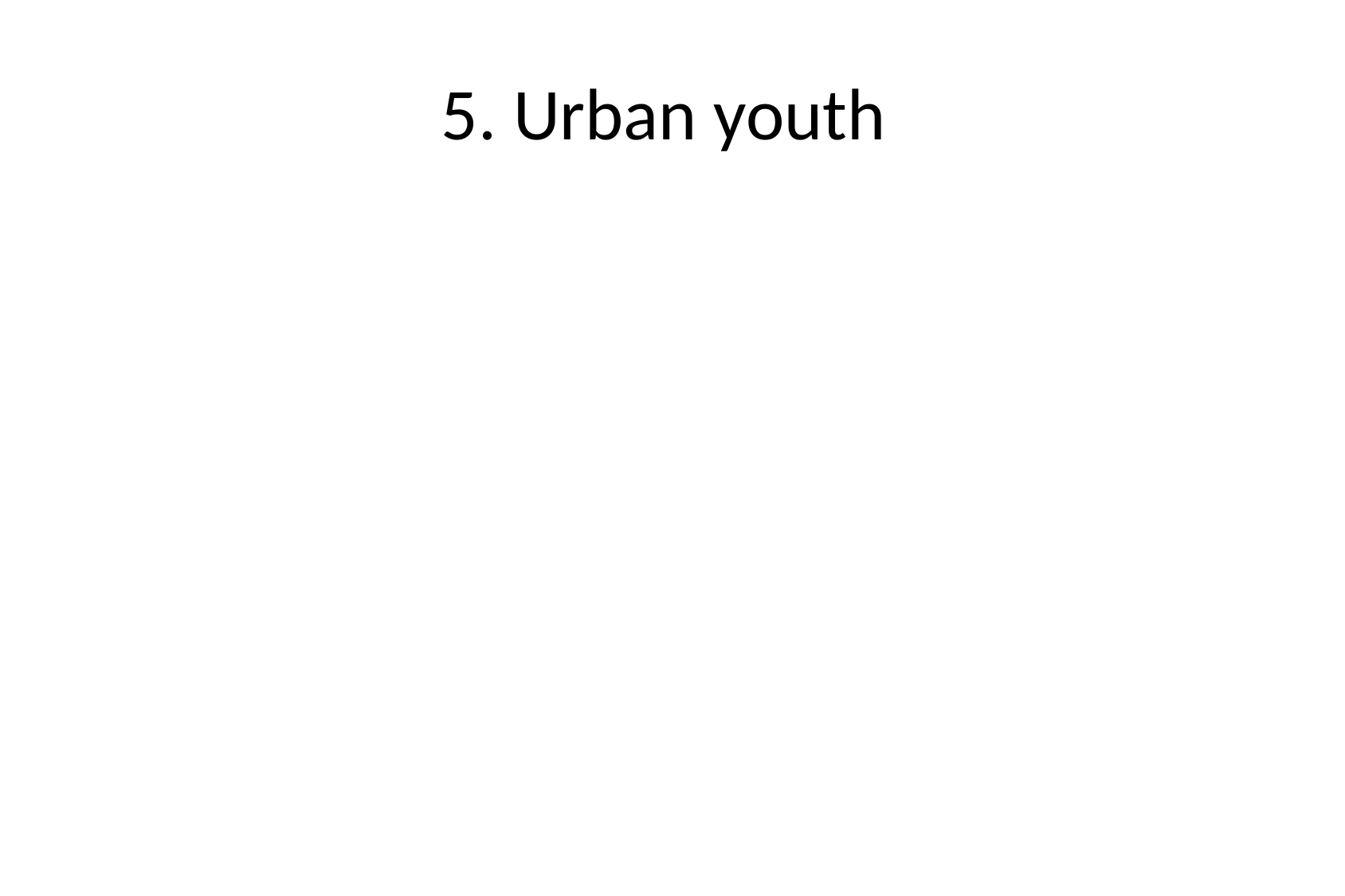

# 5. Urban youth

## Slide 24
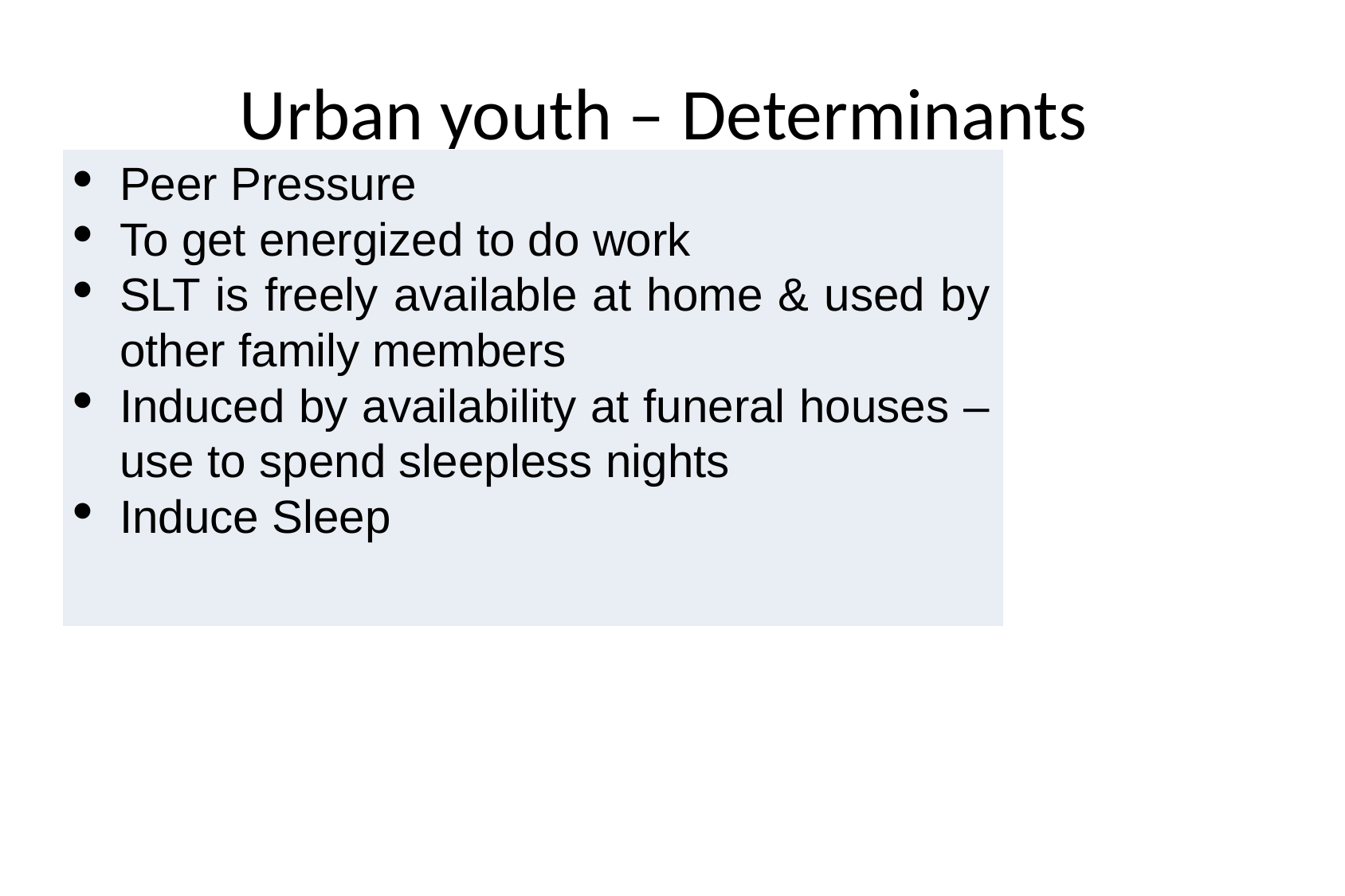

# Urban youth – Determinants
| Peer Pressure To get energized to do work SLT is freely available at home & used by other family members Induced by availability at funeral houses – use to spend sleepless nights Induce Sleep |
| --- |

## Slide 25
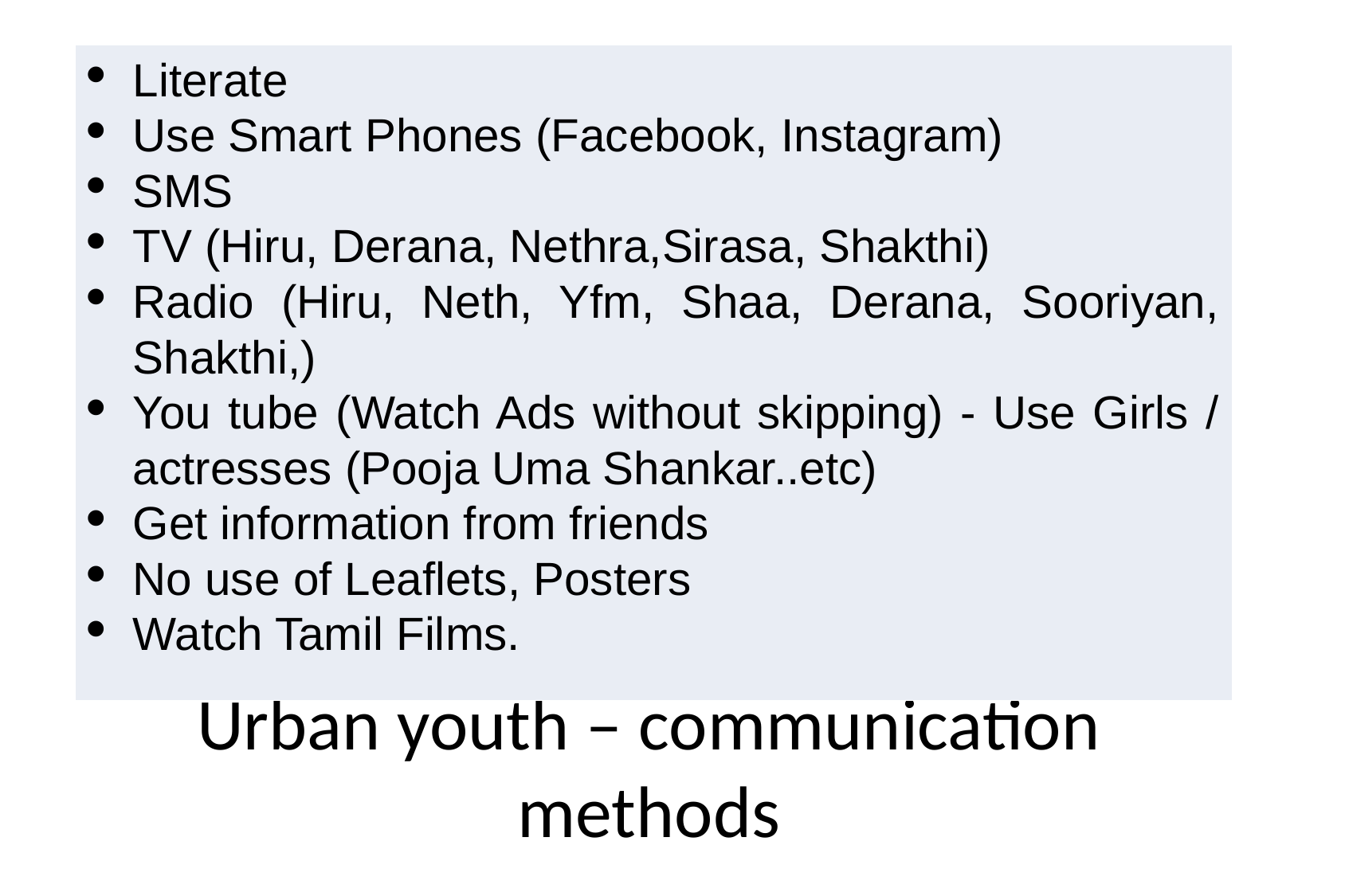

| Literate Use Smart Phones (Facebook, Instagram) SMS TV (Hiru, Derana, Nethra,Sirasa, Shakthi) Radio (Hiru, Neth, Yfm, Shaa, Derana, Sooriyan, Shakthi,) You tube (Watch Ads without skipping) - Use Girls / actresses (Pooja Uma Shankar..etc) Get information from friends No use of Leaflets, Posters Watch Tamil Films. |
| --- |
# Urban youth – communication methods

## Slide 26
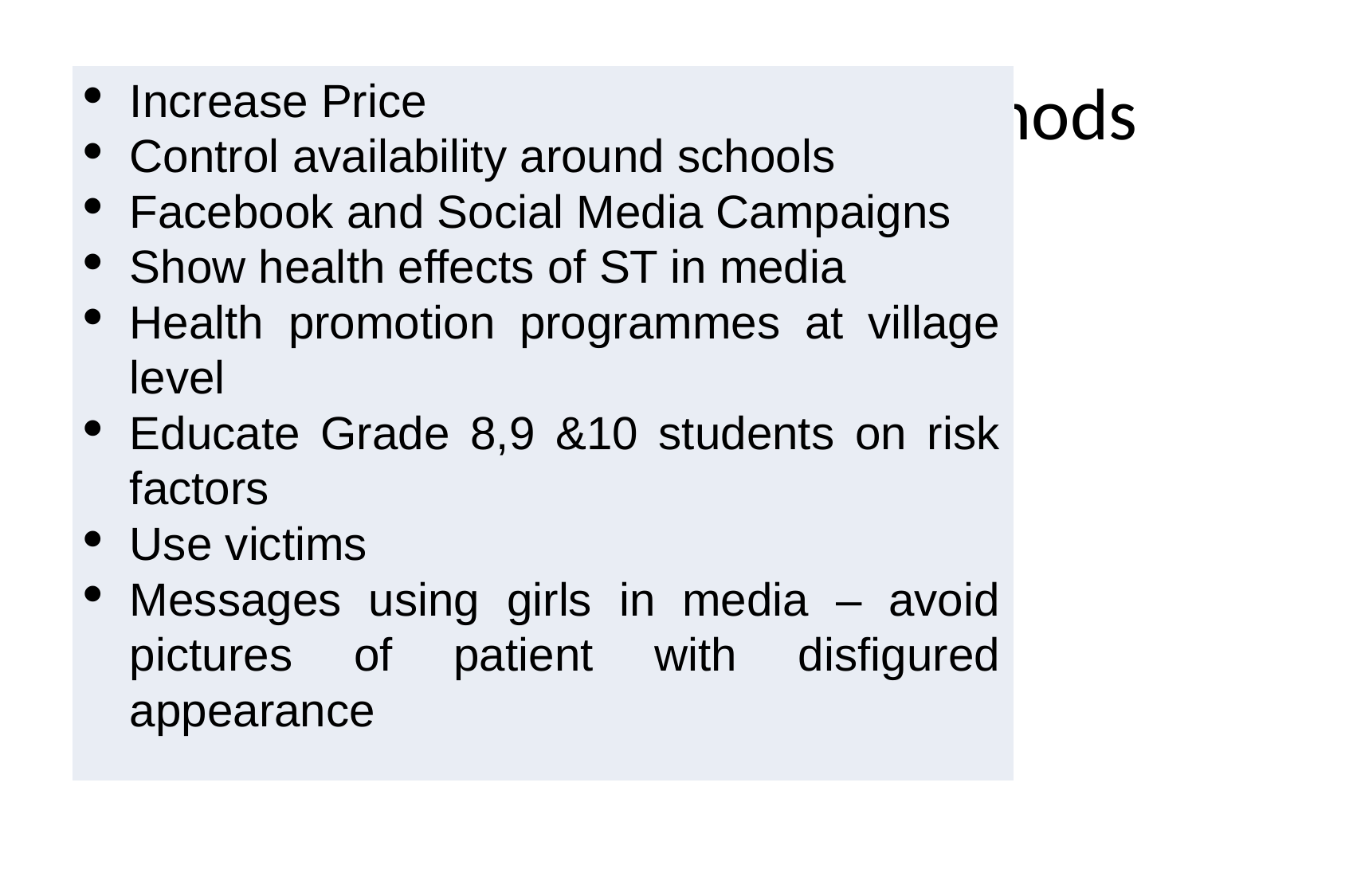

# Urban youth – control methods
| Increase Price Control availability around schools Facebook and Social Media Campaigns Show health effects of ST in media Health promotion programmes at village level Educate Grade 8,9 &10 students on risk factors Use victims Messages using girls in media – avoid pictures of patient with disfigured appearance |
| --- |

## Slide 27
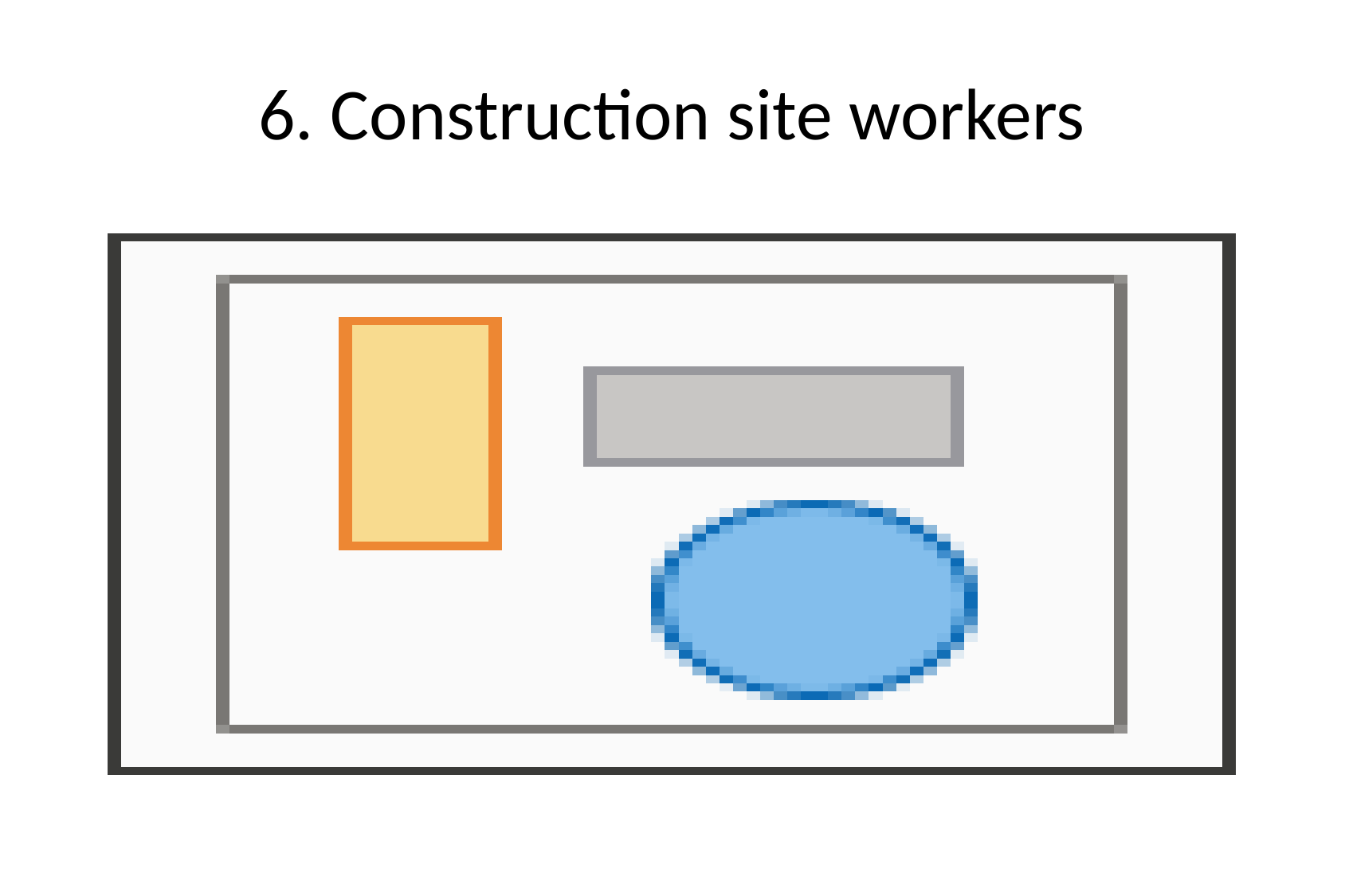

# 6. Construction site workers

## Slide 28
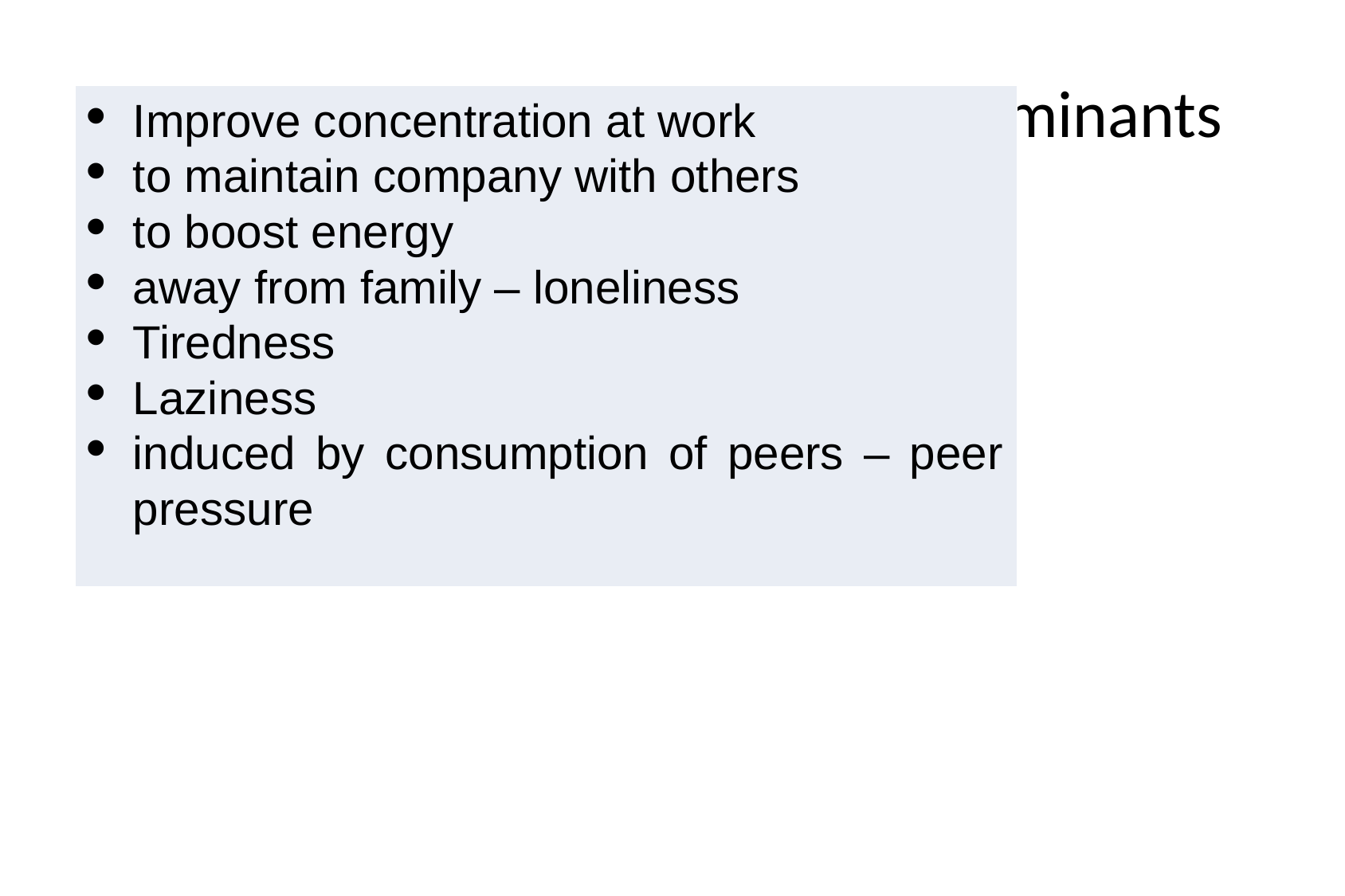

# Construction site workers – Determinants
| Improve concentration at work to maintain company with others to boost energy away from family – loneliness Tiredness Laziness induced by consumption of peers – peer pressure |
| --- |

## Slide 29
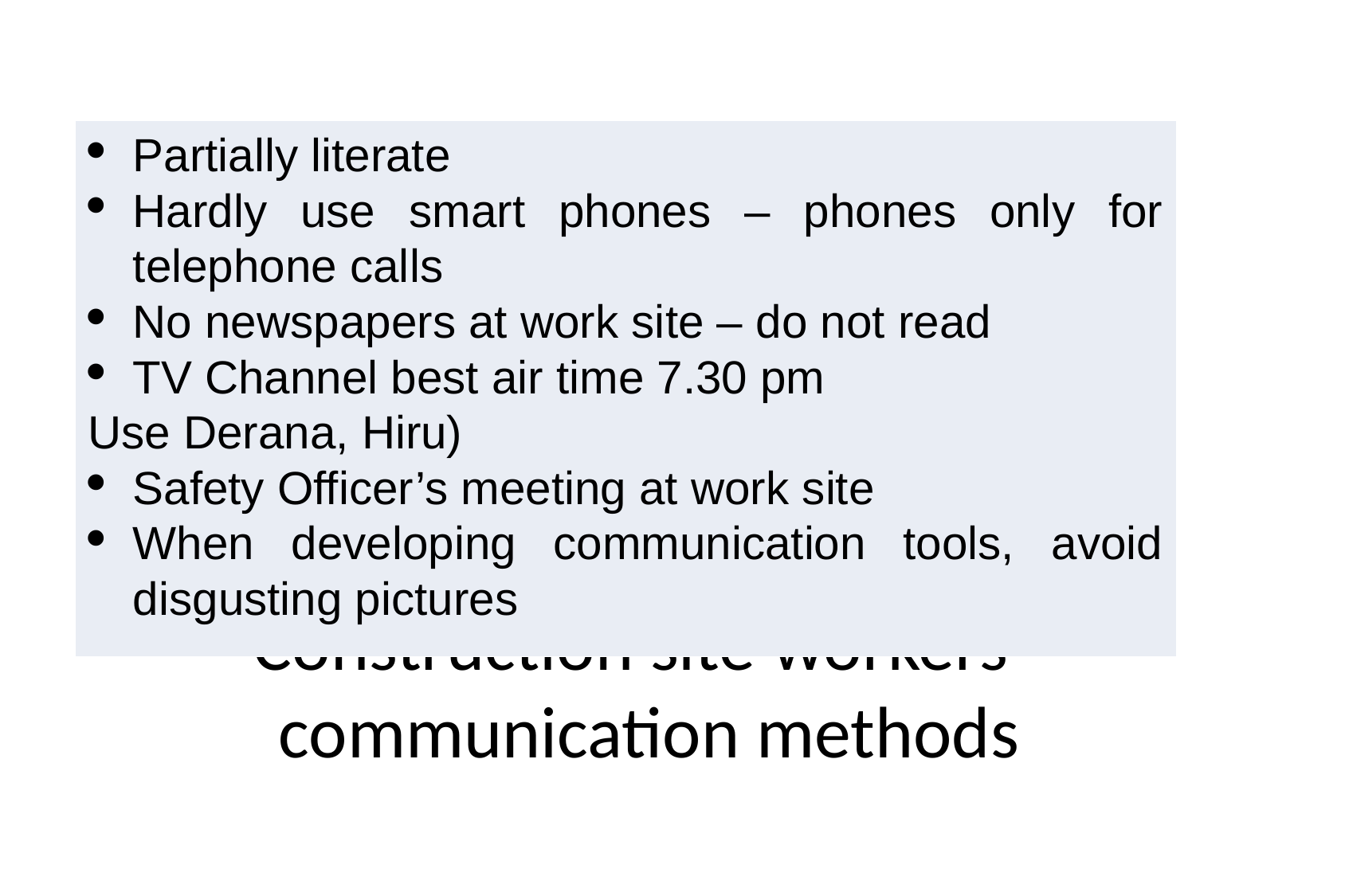

| Partially literate Hardly use smart phones – phones only for telephone calls No newspapers at work site – do not read TV Channel best air time 7.30 pm Use Derana, Hiru) Safety Officer’s meeting at work site When developing communication tools, avoid disgusting pictures |
| --- |
# Construction site workers – communication methods

## Slide 30
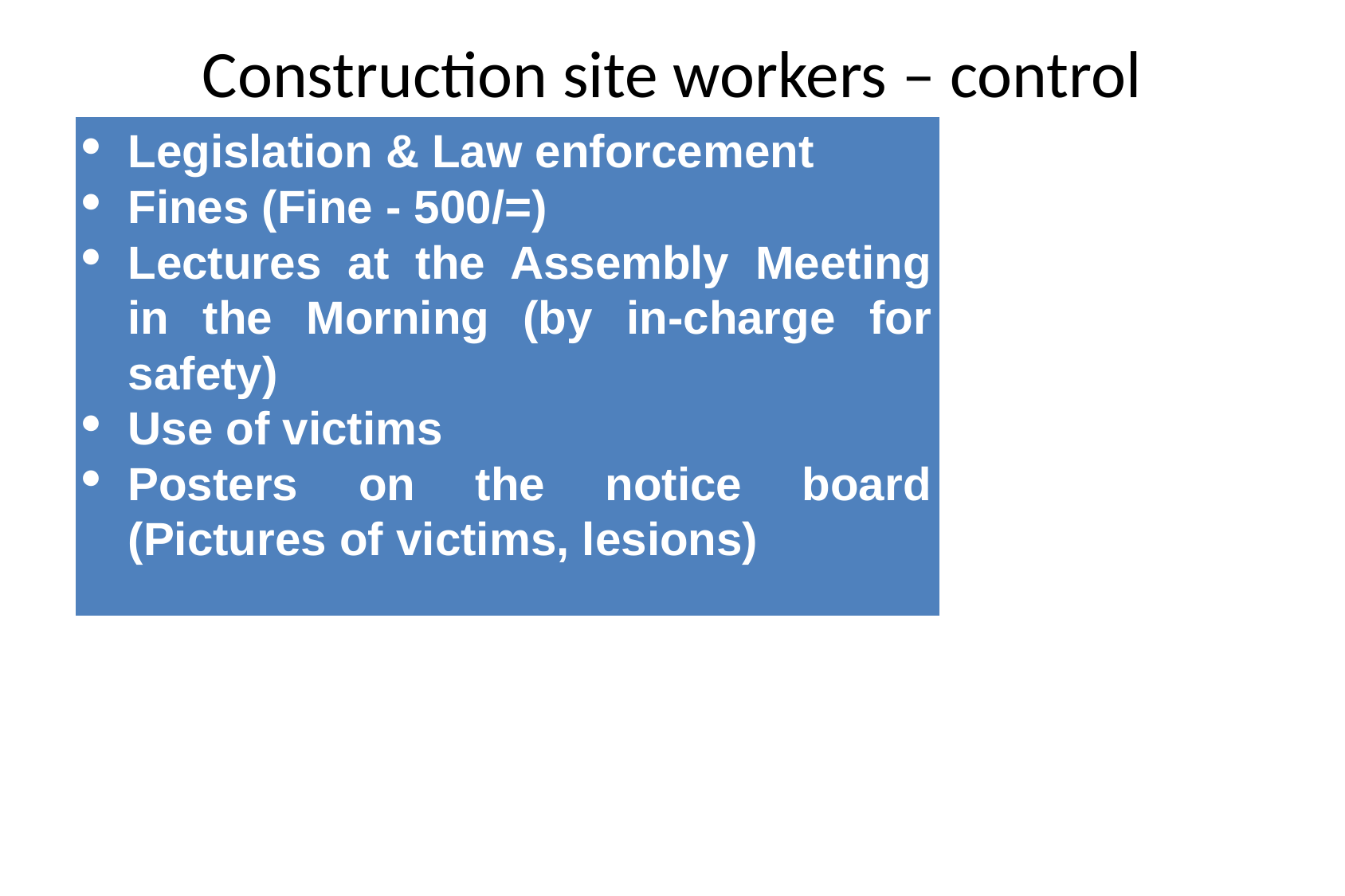

# Construction site workers – control methods
| Legislation & Law enforcement Fines (Fine - 500/=) Lectures at the Assembly Meeting in the Morning (by in-charge for safety) Use of victims Posters on the notice board (Pictures of victims, lesions) |
| --- |

## Slide 31
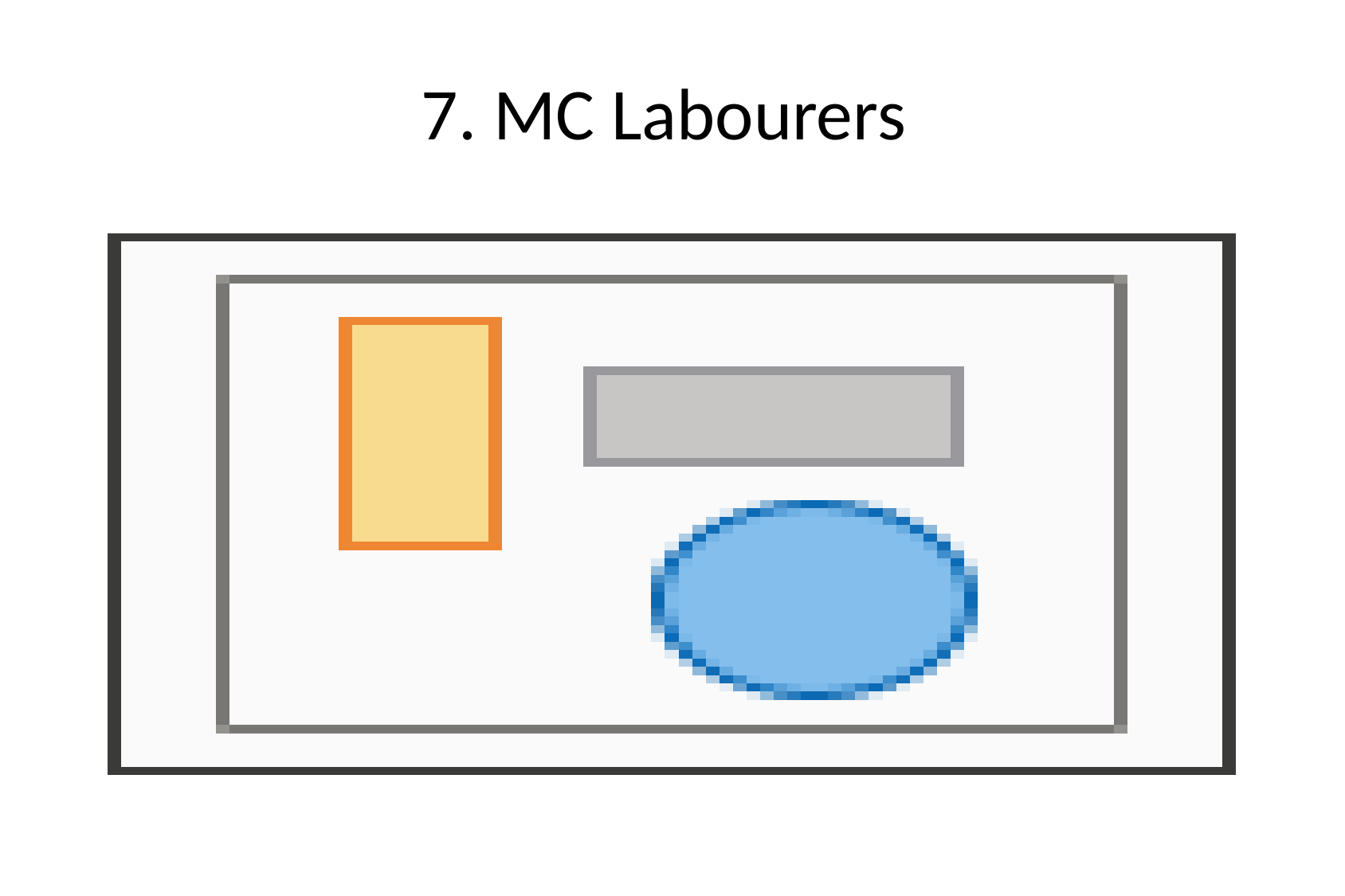

# 7. MC Labourers

## Slide 32
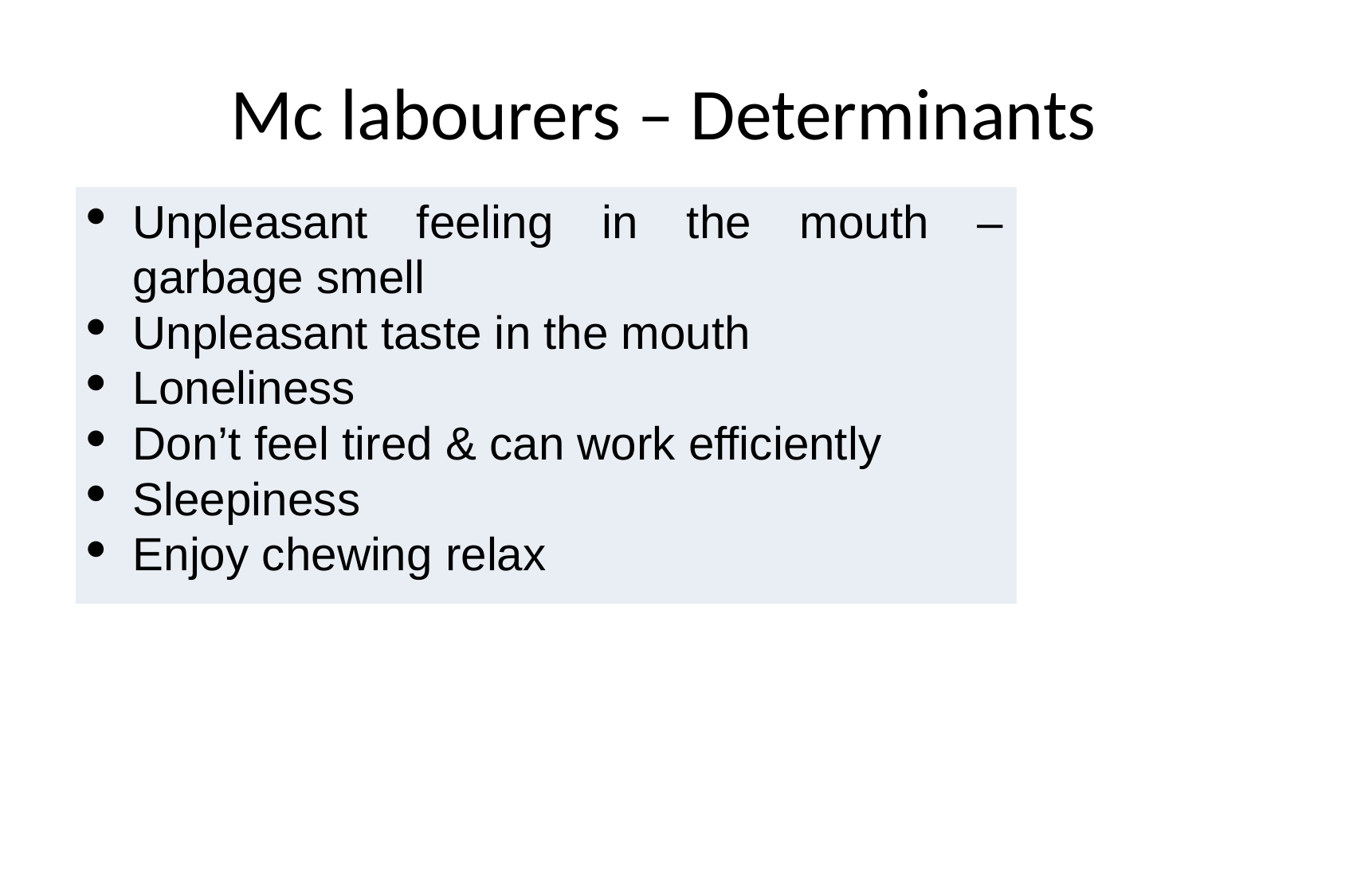

# Mc labourers – Determinants
| Unpleasant feeling in the mouth – garbage smell Unpleasant taste in the mouth Loneliness Don’t feel tired & can work efficiently Sleepiness Enjoy chewing relax |
| --- |

## Slide 33
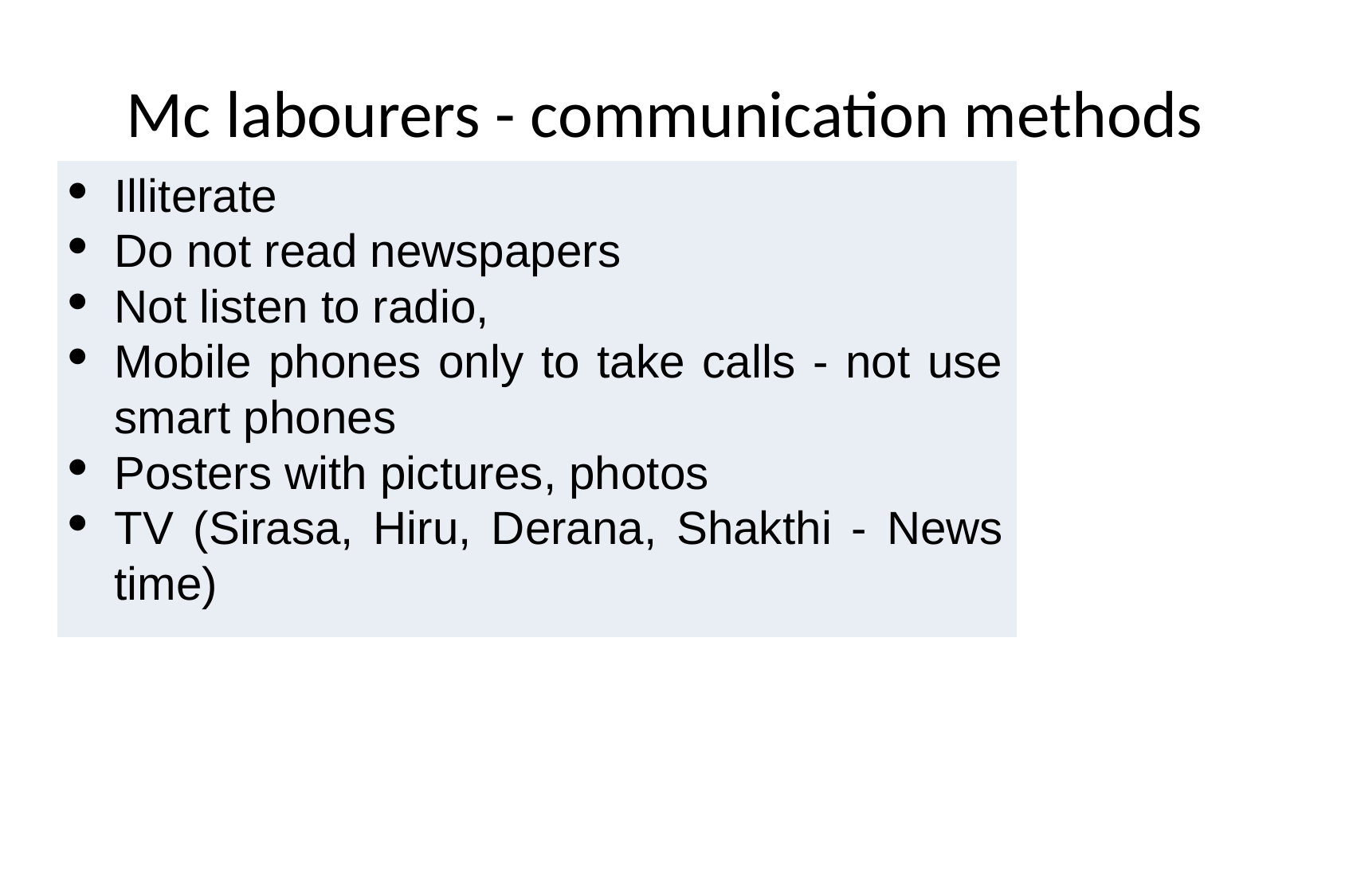

# Mc labourers - communication methods
| Illiterate Do not read newspapers Not listen to radio, Mobile phones only to take calls - not use smart phones Posters with pictures, photos TV (Sirasa, Hiru, Derana, Shakthi - News time) |
| --- |

## Slide 34
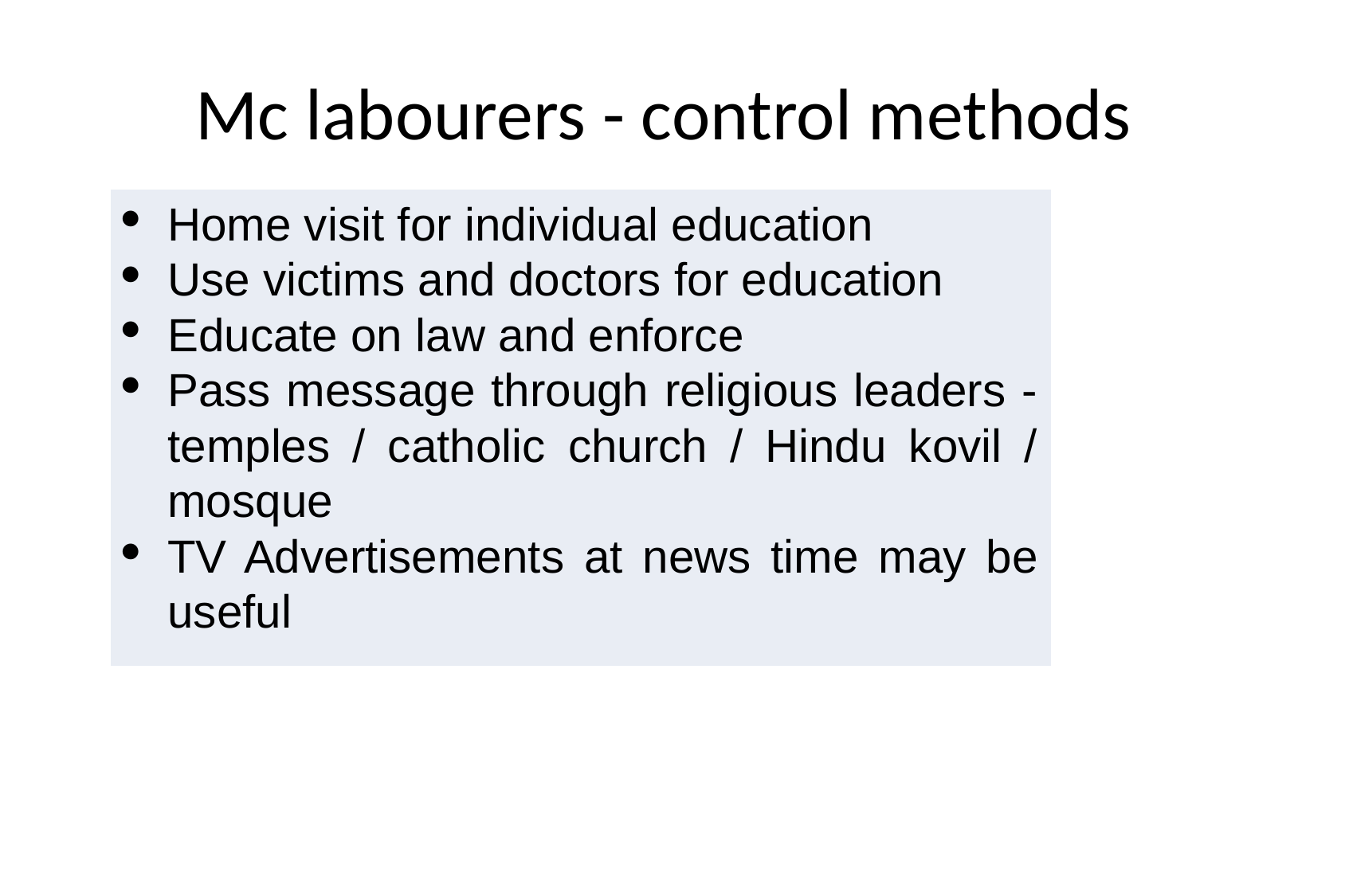

# Mc labourers - control methods
| Home visit for individual education Use victims and doctors for education Educate on law and enforce Pass message through religious leaders - temples / catholic church / Hindu kovil / mosque TV Advertisements at news time may be useful |
| --- |

## Slide 35
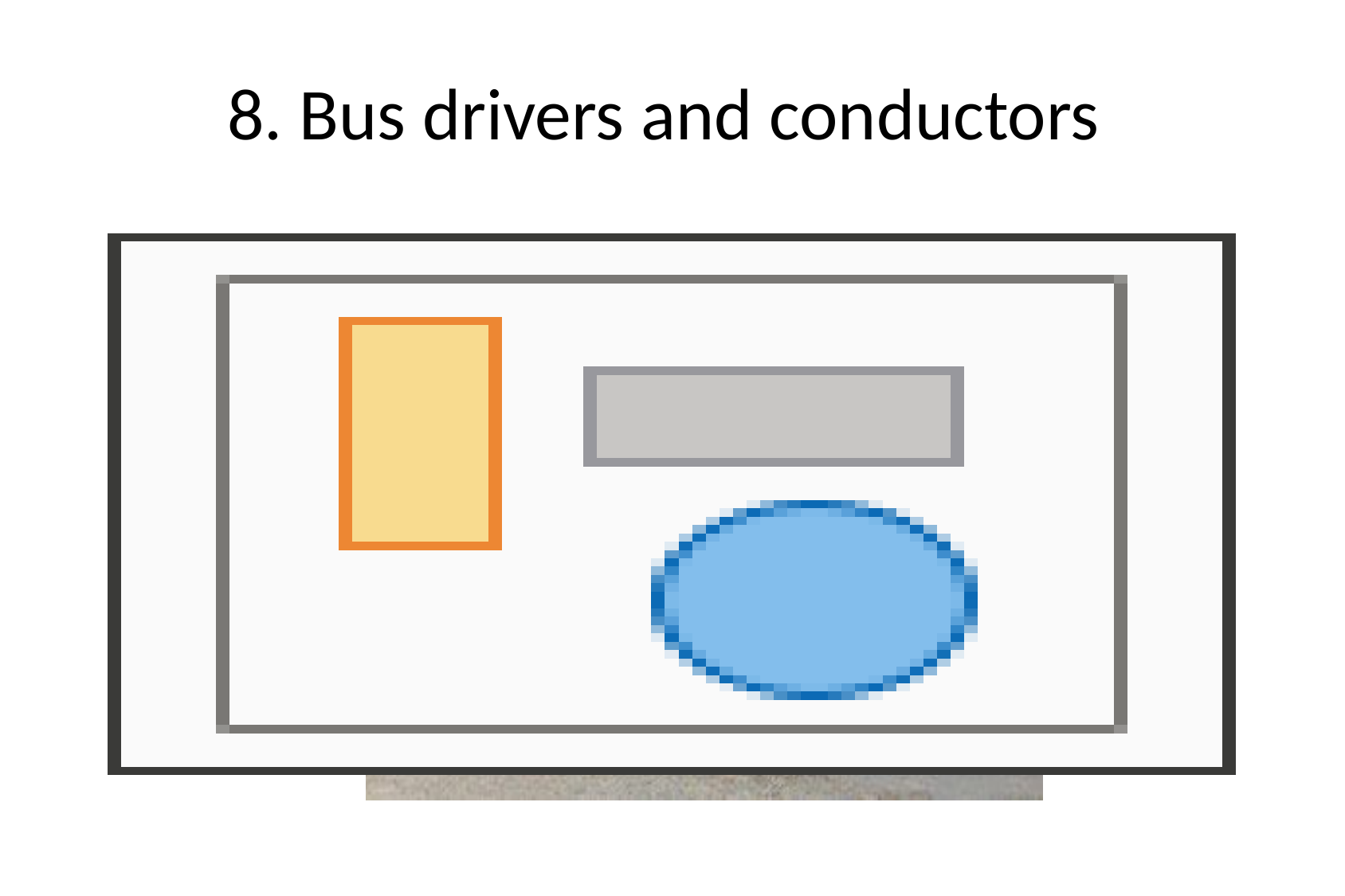

# 8. Bus drivers and conductors

## Slide 36
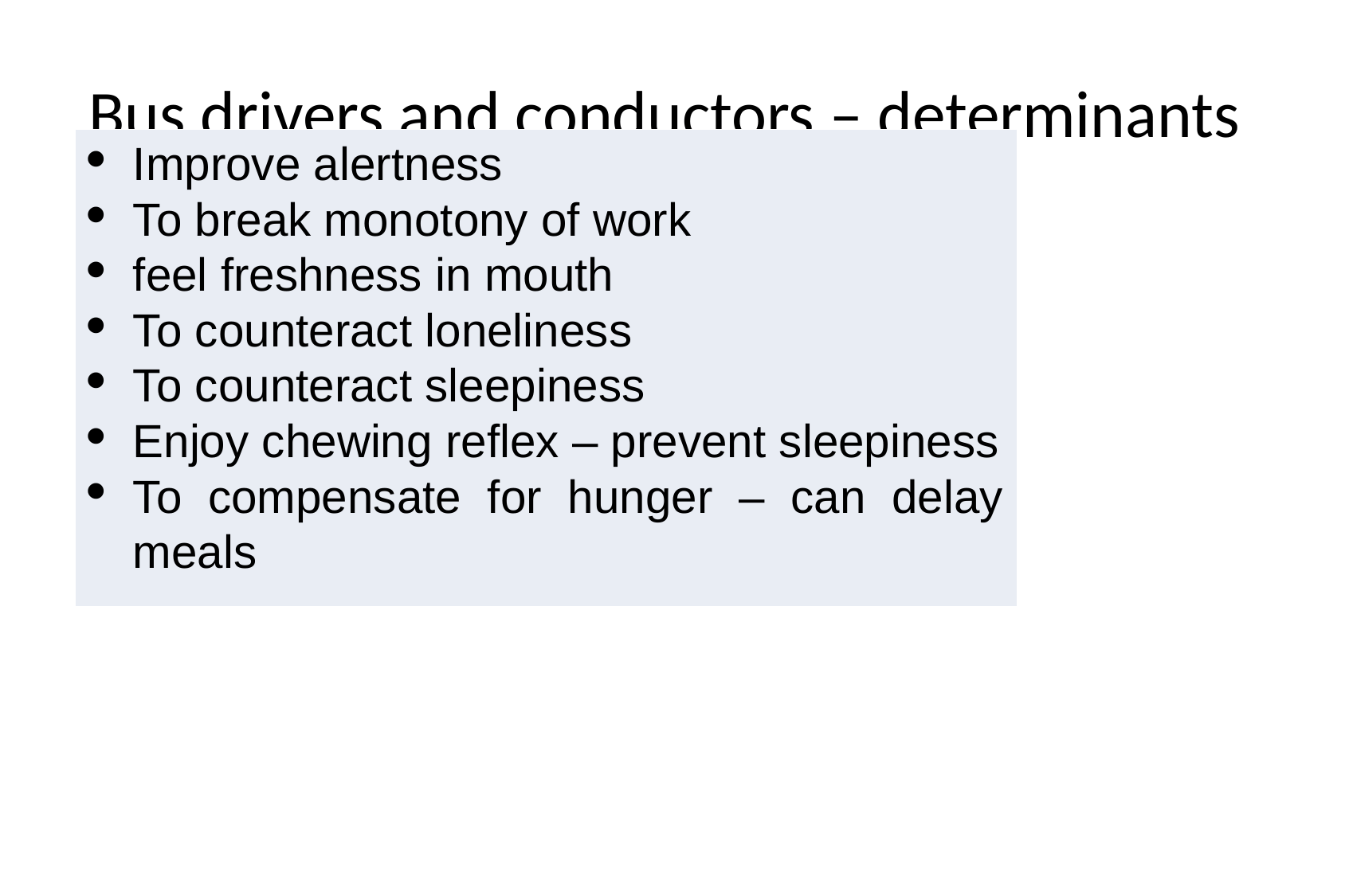

# Bus drivers and conductors – determinants
| Improve alertness To break monotony of work feel freshness in mouth To counteract loneliness To counteract sleepiness Enjoy chewing reflex – prevent sleepiness To compensate for hunger – can delay meals |
| --- |

## Slide 37
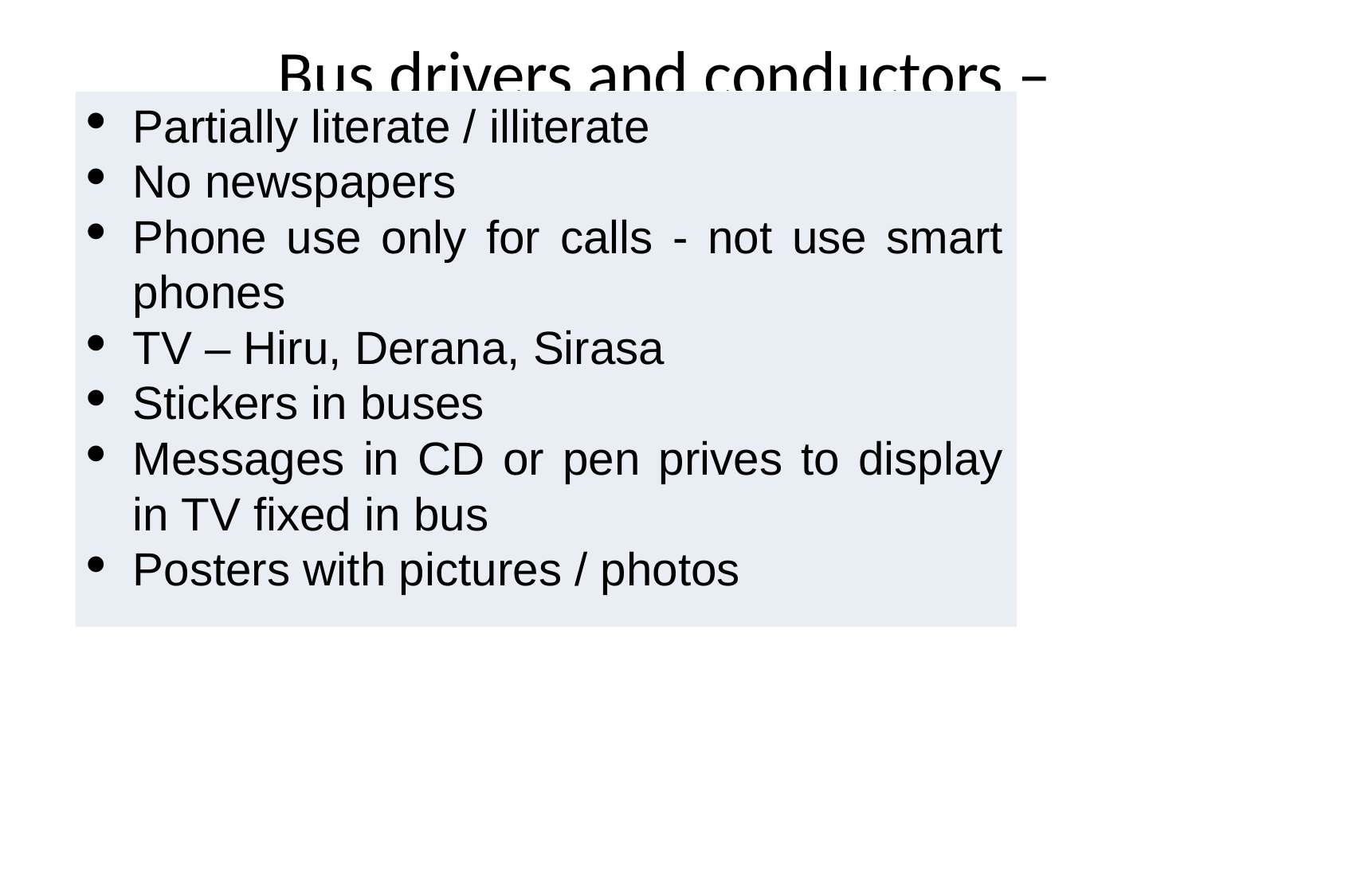

# Bus drivers and conductors – communication methods
| Partially literate / illiterate No newspapers Phone use only for calls - not use smart phones TV – Hiru, Derana, Sirasa Stickers in buses Messages in CD or pen prives to display in TV fixed in bus Posters with pictures / photos |
| --- |

## Slide 38
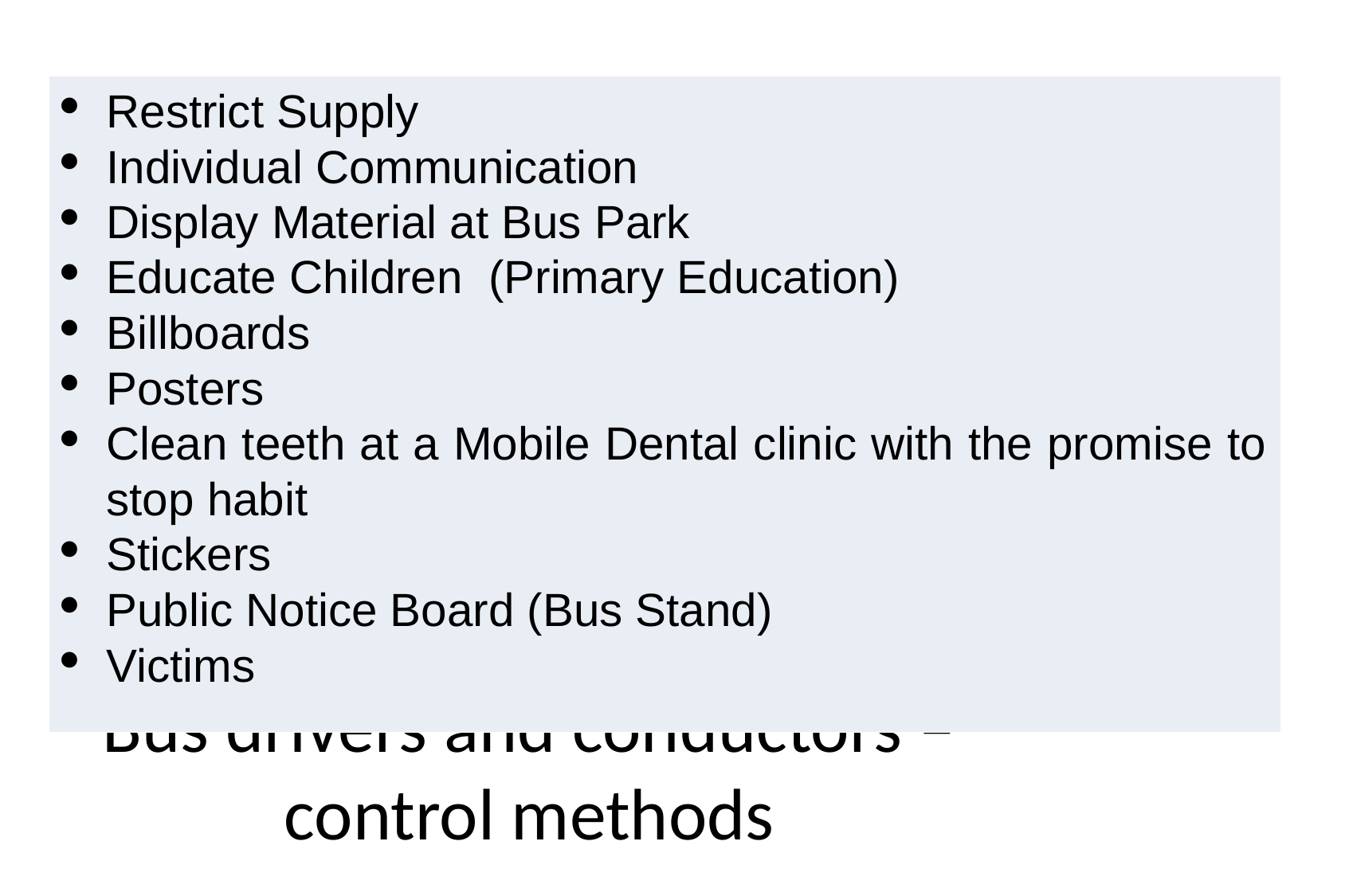

| Restrict Supply Individual Communication Display Material at Bus Park Educate Children (Primary Education) Billboards Posters Clean teeth at a Mobile Dental clinic with the promise to stop habit Stickers Public Notice Board (Bus Stand) Victims |
| --- |
# Bus drivers and conductors – control methods

## Slide 39
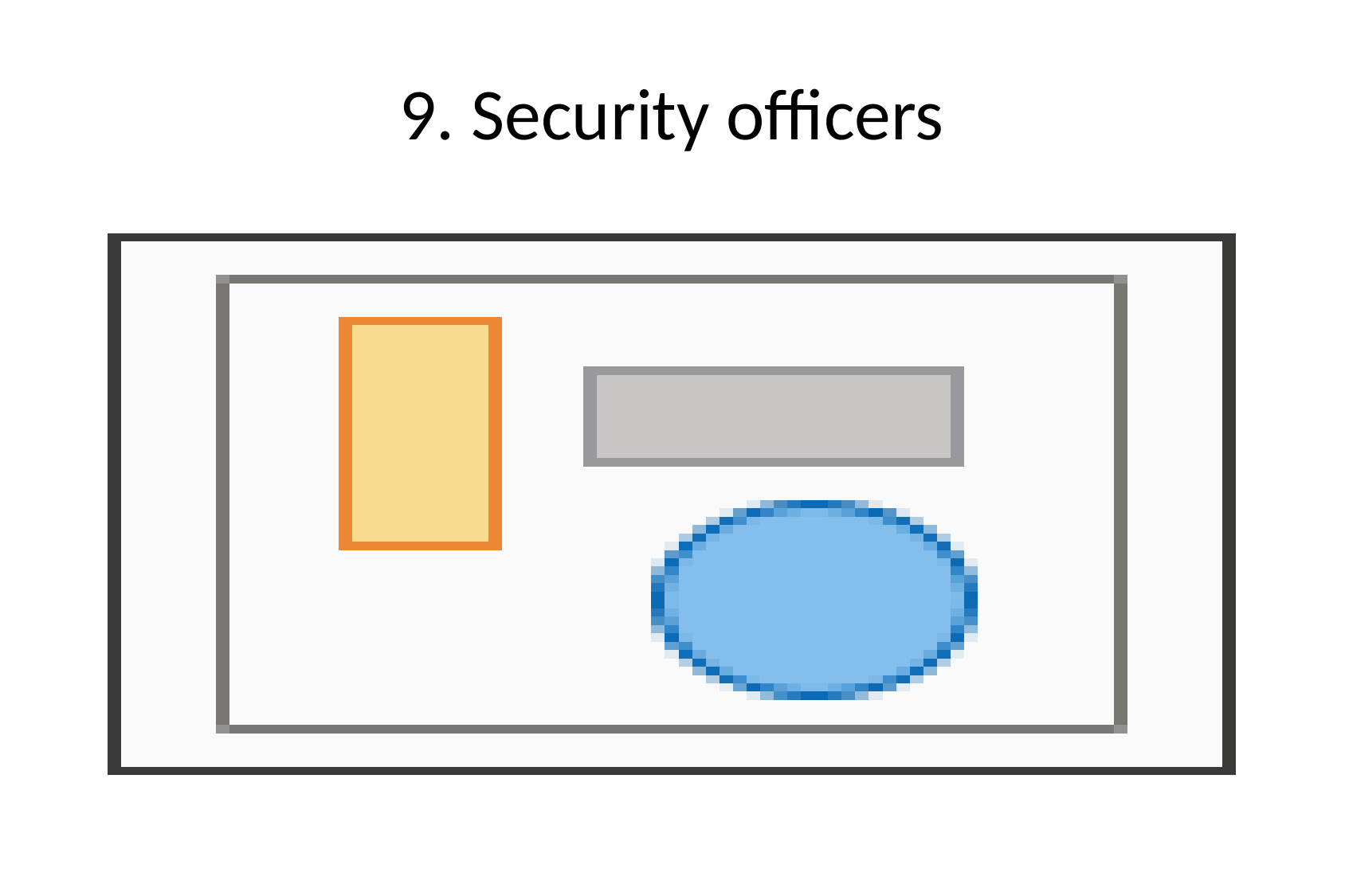

# 9. Security officers

## Slide 40
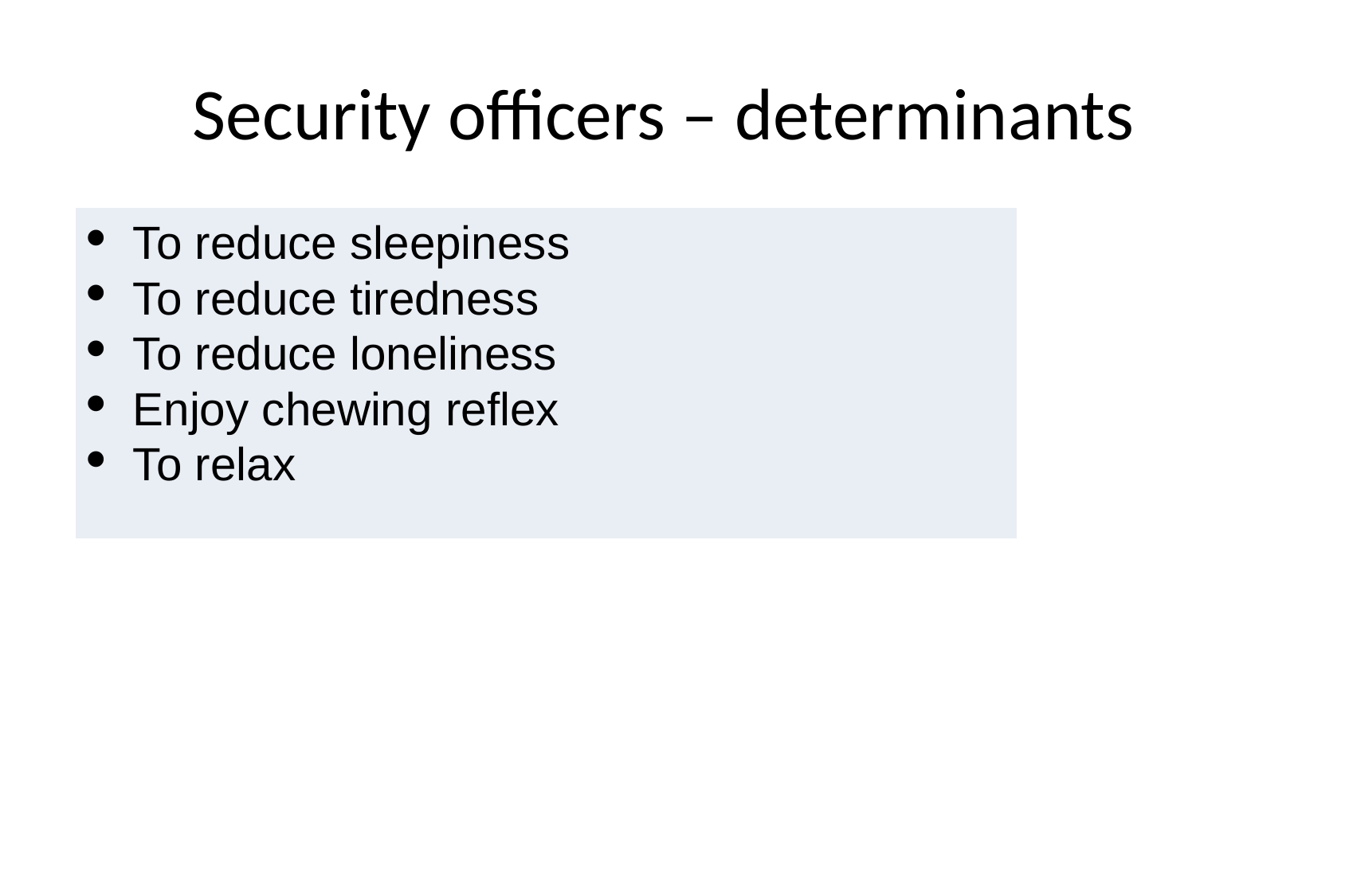

# Security officers – determinants
| To reduce sleepiness To reduce tiredness To reduce loneliness Enjoy chewing reflex To relax |
| --- |

## Slide 41
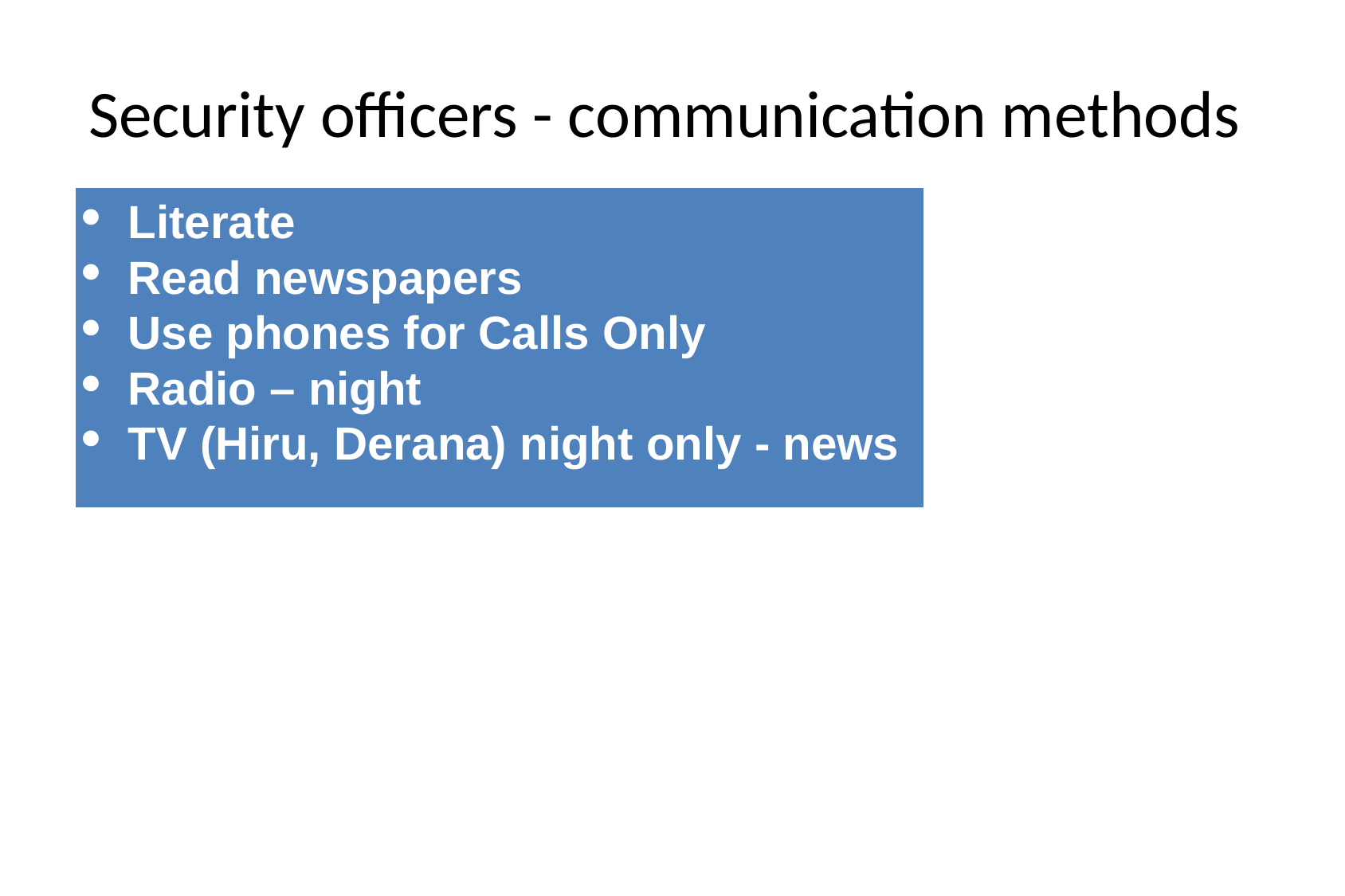

# Security officers - communication methods
| Literate Read newspapers Use phones for Calls Only Radio – night TV (Hiru, Derana) night only - news |
| --- |

## Slide 42
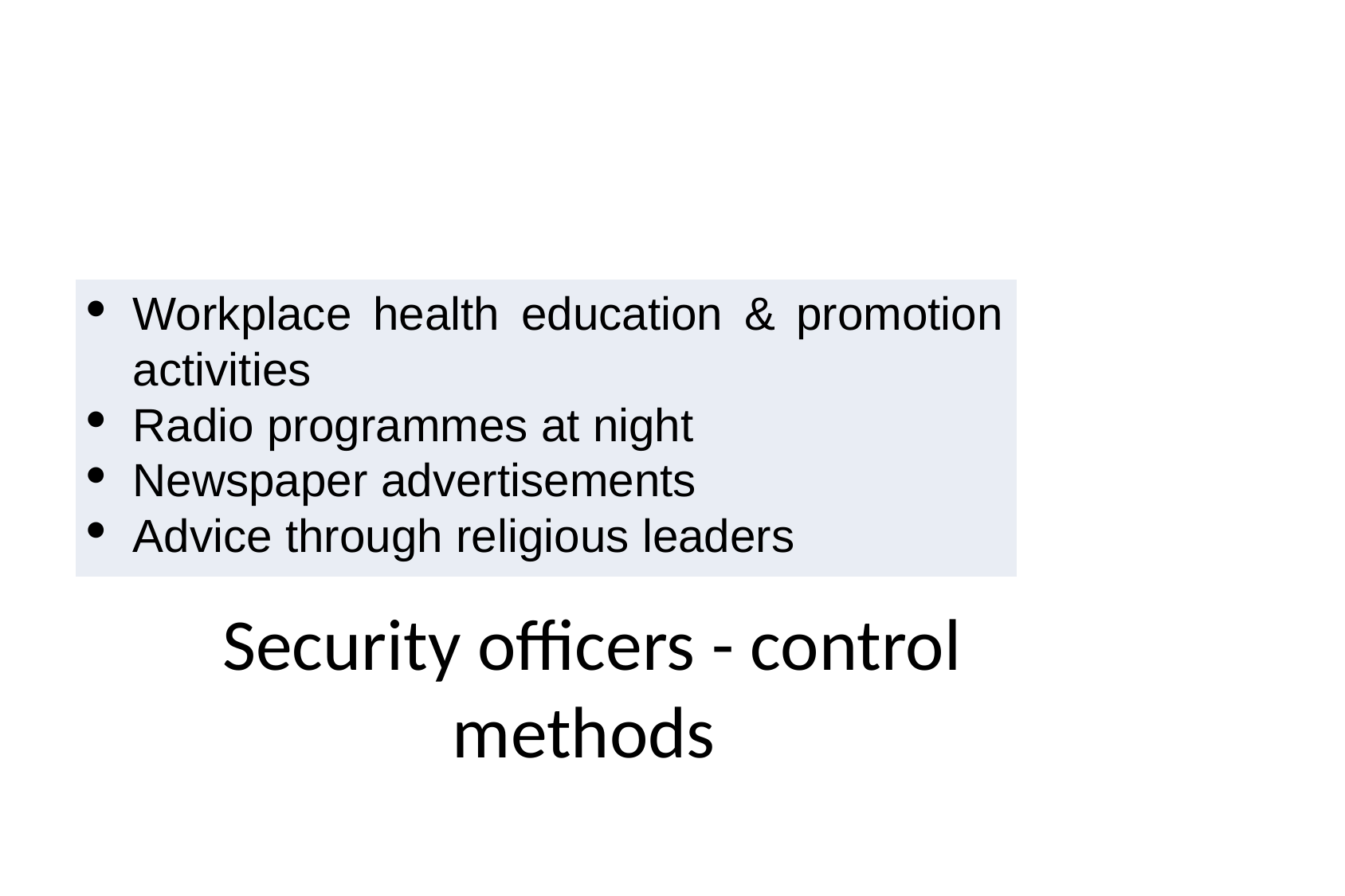

| Workplace health education & promotion activities Radio programmes at night Newspaper advertisements Advice through religious leaders |
| --- |
# Security officers - control methods

## Slide 43
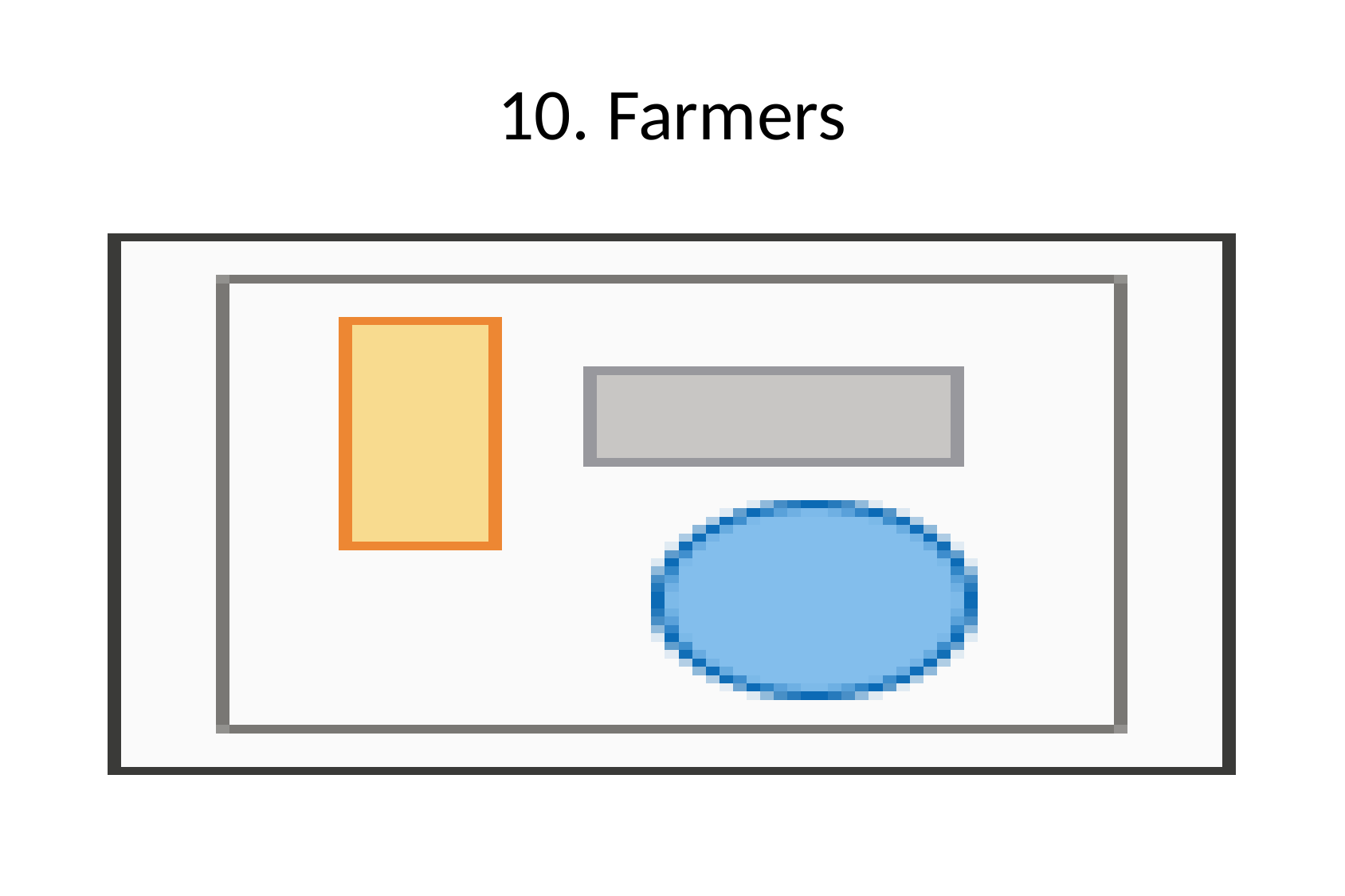

# 10. Farmers

## Slide 44
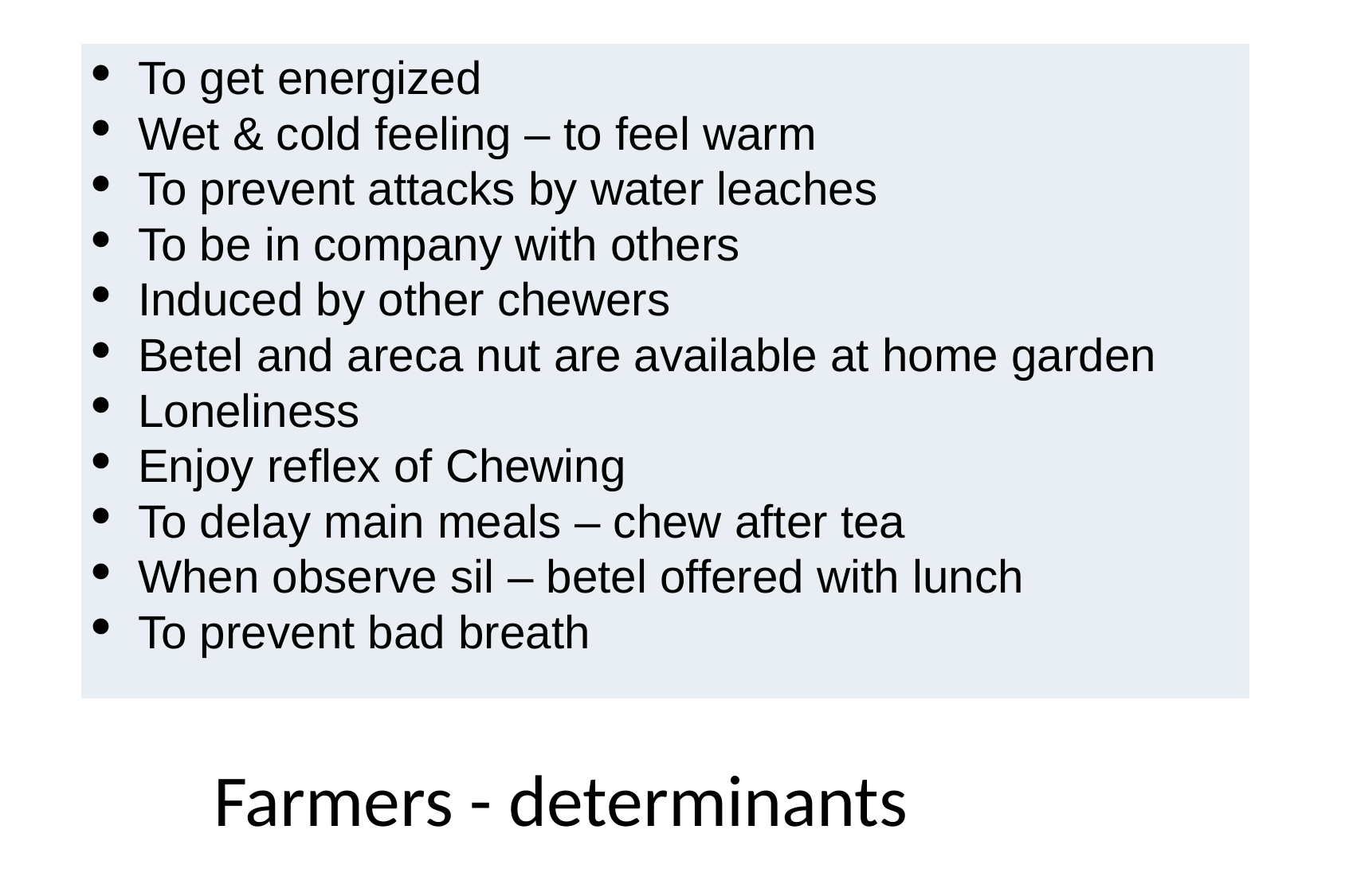

| To get energized Wet & cold feeling – to feel warm To prevent attacks by water leaches To be in company with others Induced by other chewers Betel and areca nut are available at home garden Loneliness Enjoy reflex of Chewing To delay main meals – chew after tea When observe sil – betel offered with lunch To prevent bad breath |
| --- |
# Farmers - determinants

## Slide 45
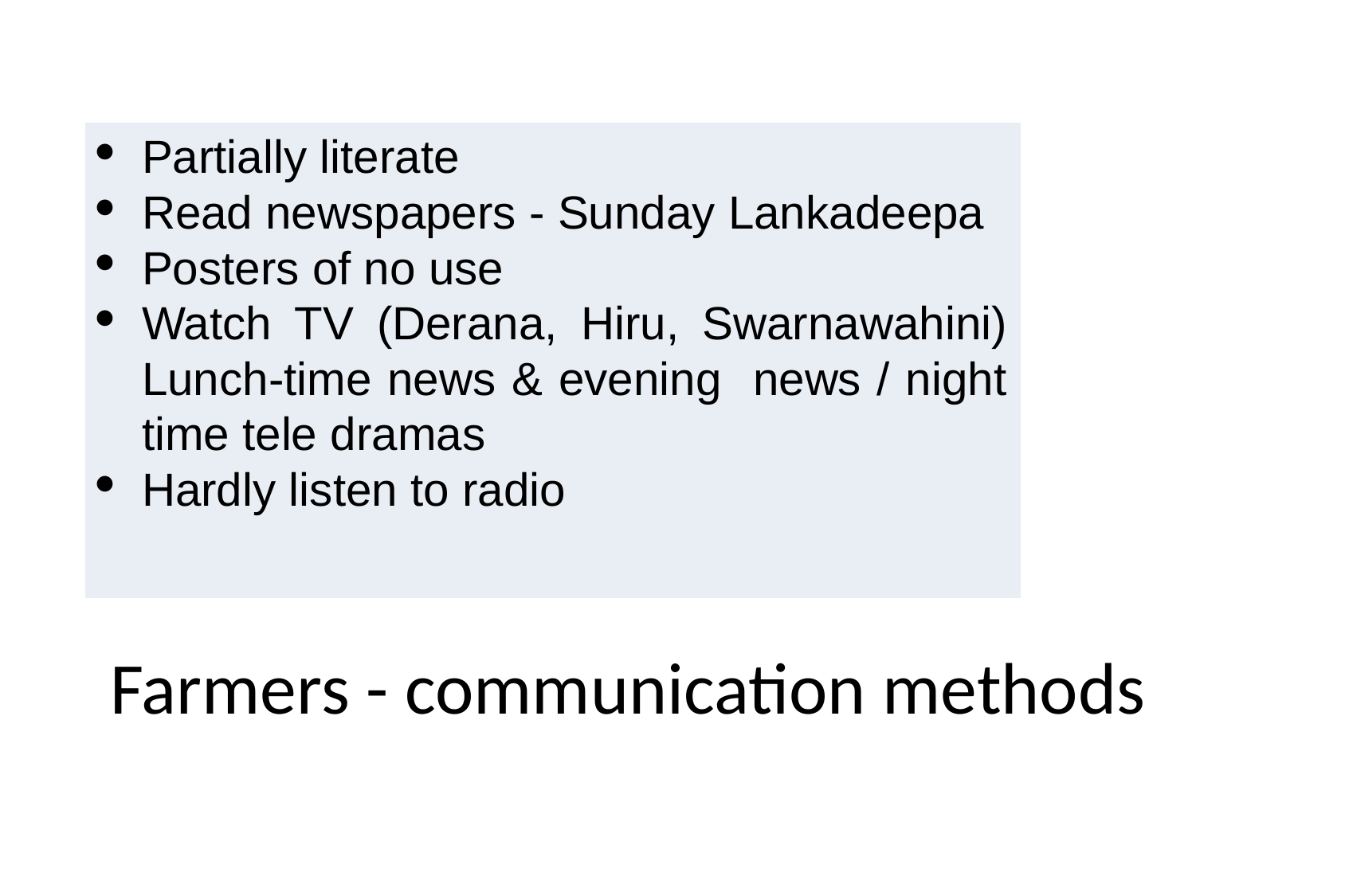

| Partially literate Read newspapers - Sunday Lankadeepa Posters of no use Watch TV (Derana, Hiru, Swarnawahini) Lunch-time news & evening news / night time tele dramas Hardly listen to radio |
| --- |
# Farmers - communication methods

## Slide 46
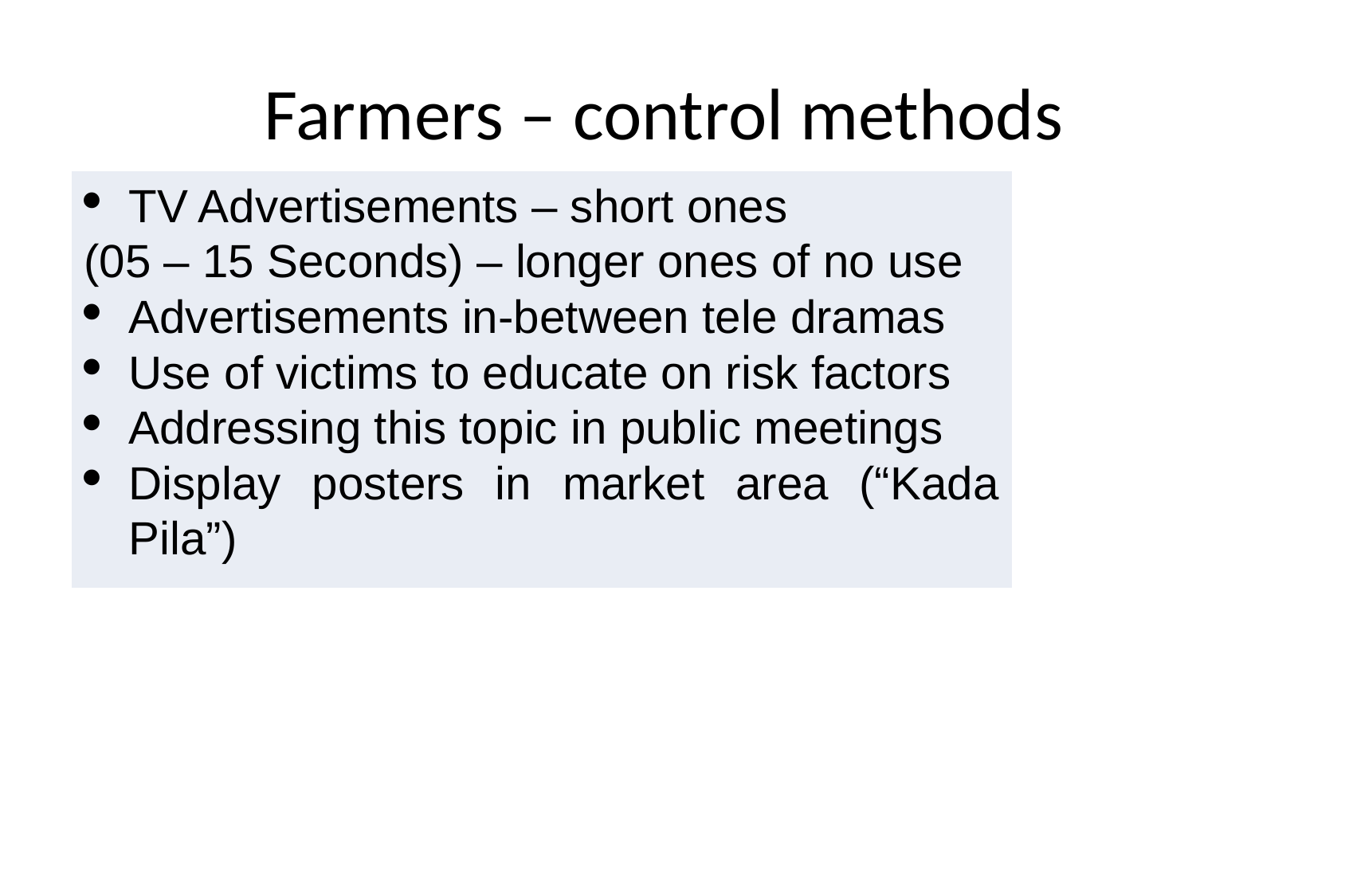

# Farmers – control methods
| TV Advertisements – short ones (05 – 15 Seconds) – longer ones of no use Advertisements in-between tele dramas Use of victims to educate on risk factors Addressing this topic in public meetings Display posters in market area (“Kada Pila”) |
| --- |

## Slide 47
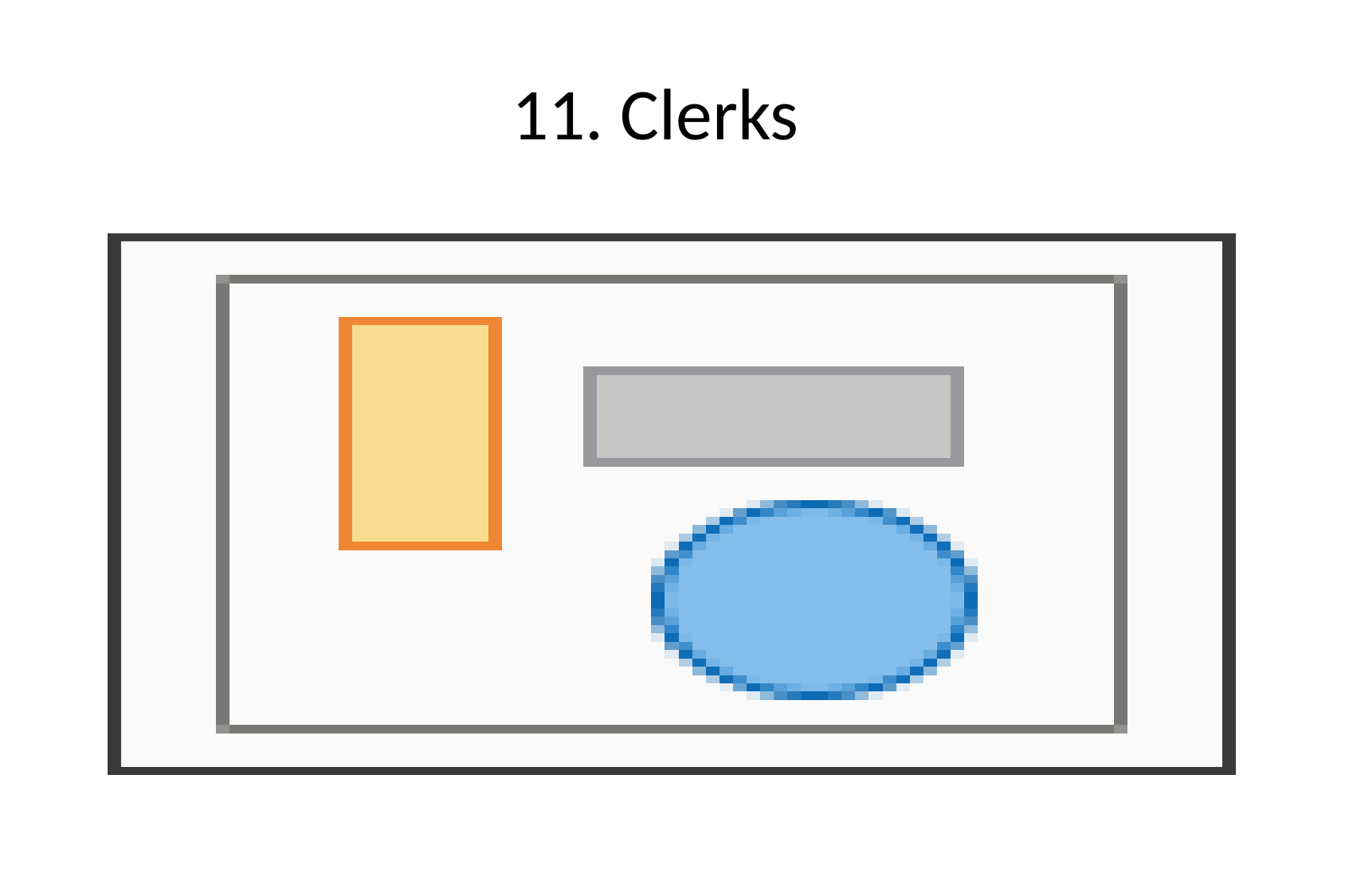

# 11. Clerks

## Slide 48
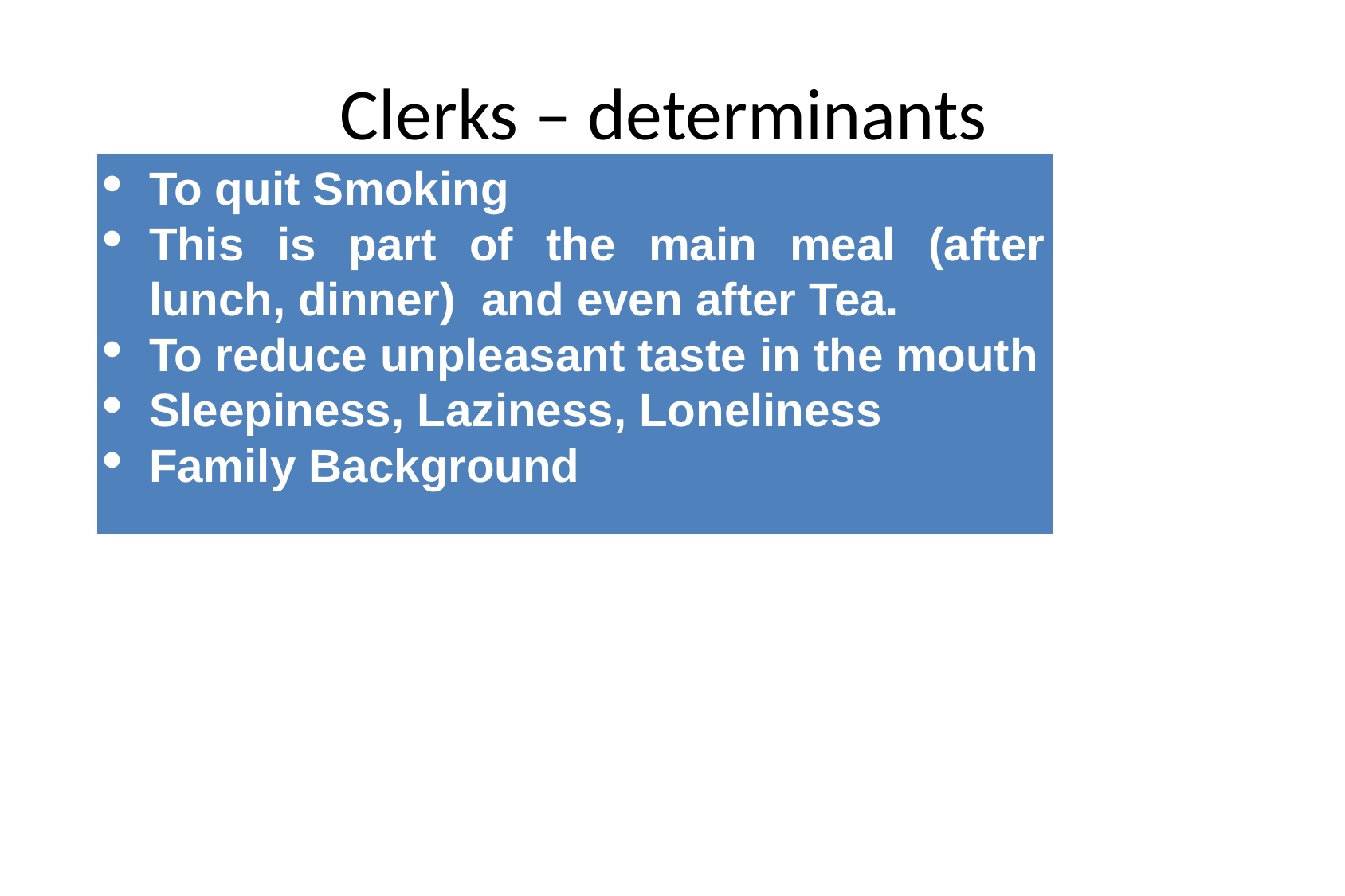

# Clerks – determinants
| To quit Smoking This is part of the main meal (after lunch, dinner) and even after Tea. To reduce unpleasant taste in the mouth Sleepiness, Laziness, Loneliness Family Background |
| --- |

## Slide 49
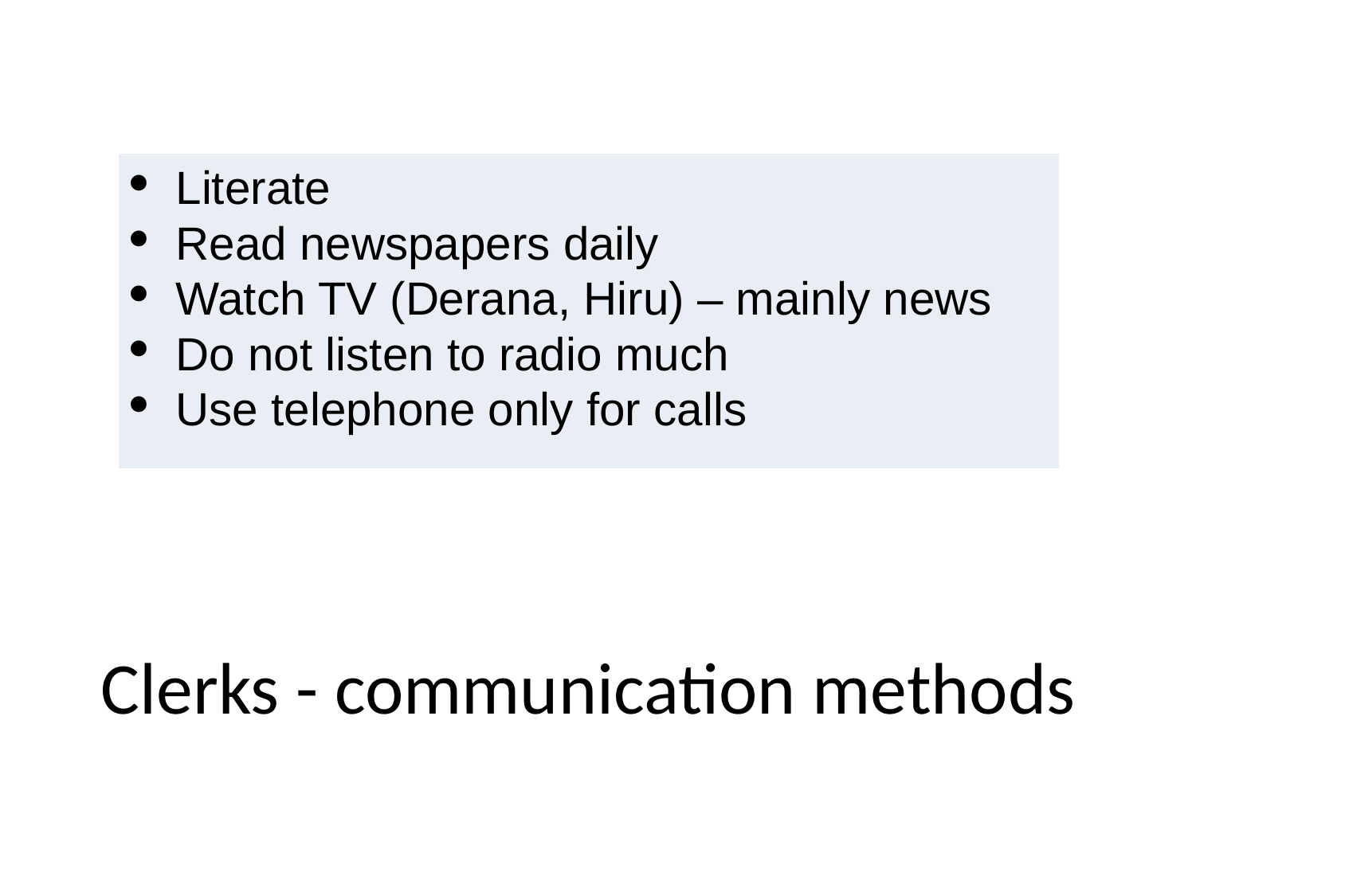

| Literate Read newspapers daily Watch TV (Derana, Hiru) – mainly news Do not listen to radio much Use telephone only for calls |
| --- |
# Clerks - communication methods

## Slide 50
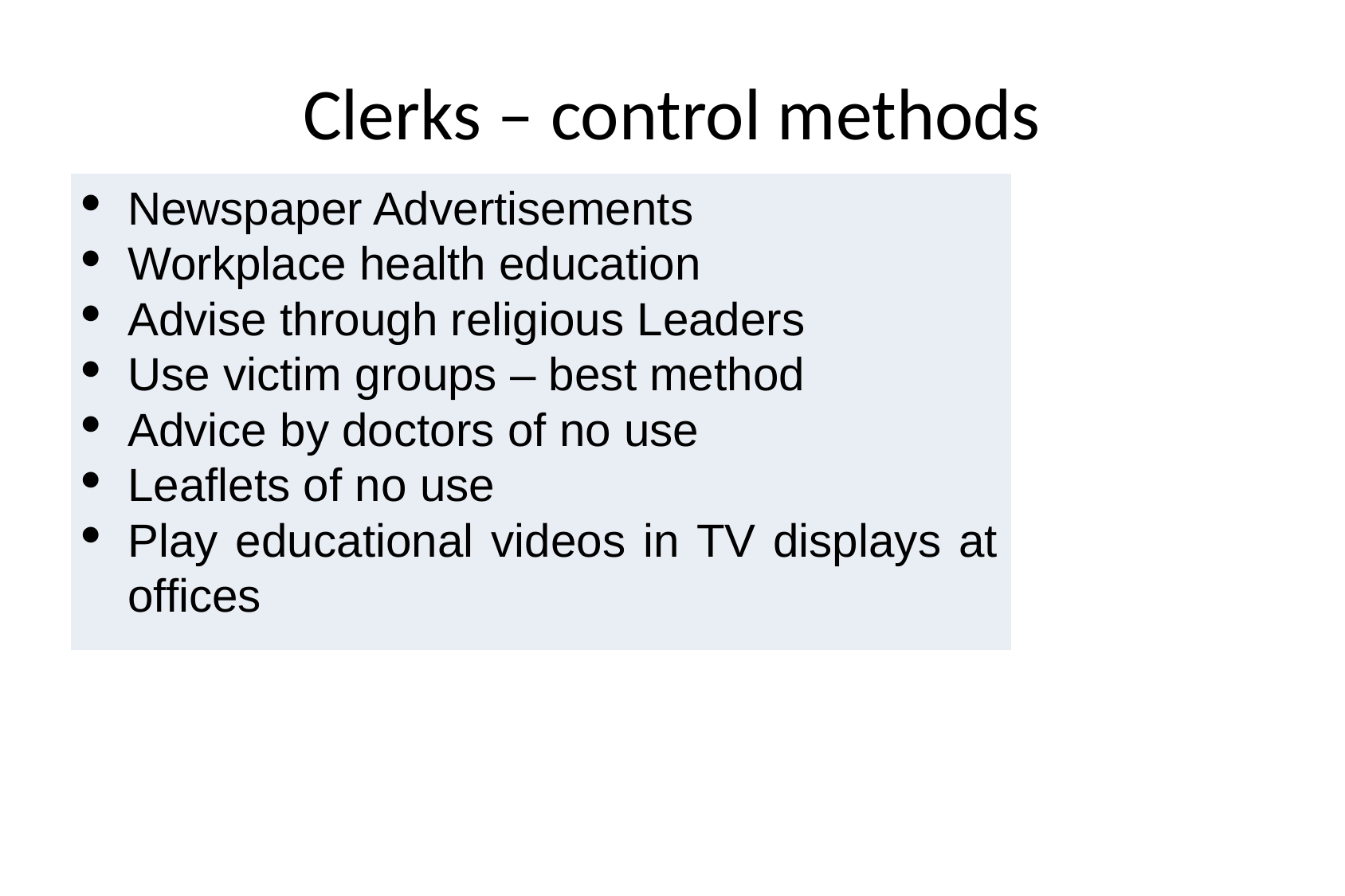

# Clerks – control methods
| Newspaper Advertisements Workplace health education Advise through religious Leaders Use victim groups – best method Advice by doctors of no use Leaflets of no use Play educational videos in TV displays at offices |
| --- |

## Slide 51
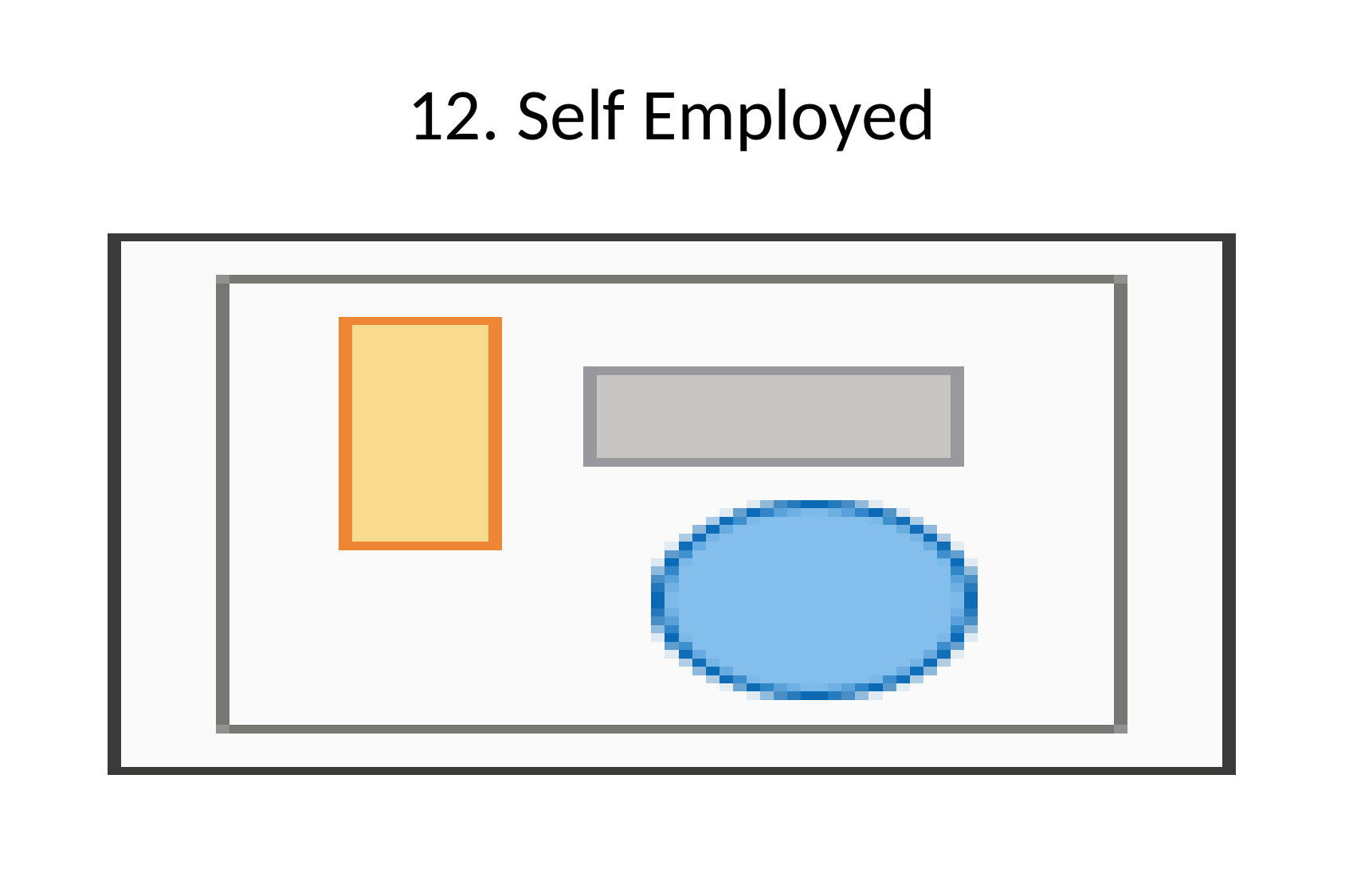

# 12. Self Employed

## Slide 52
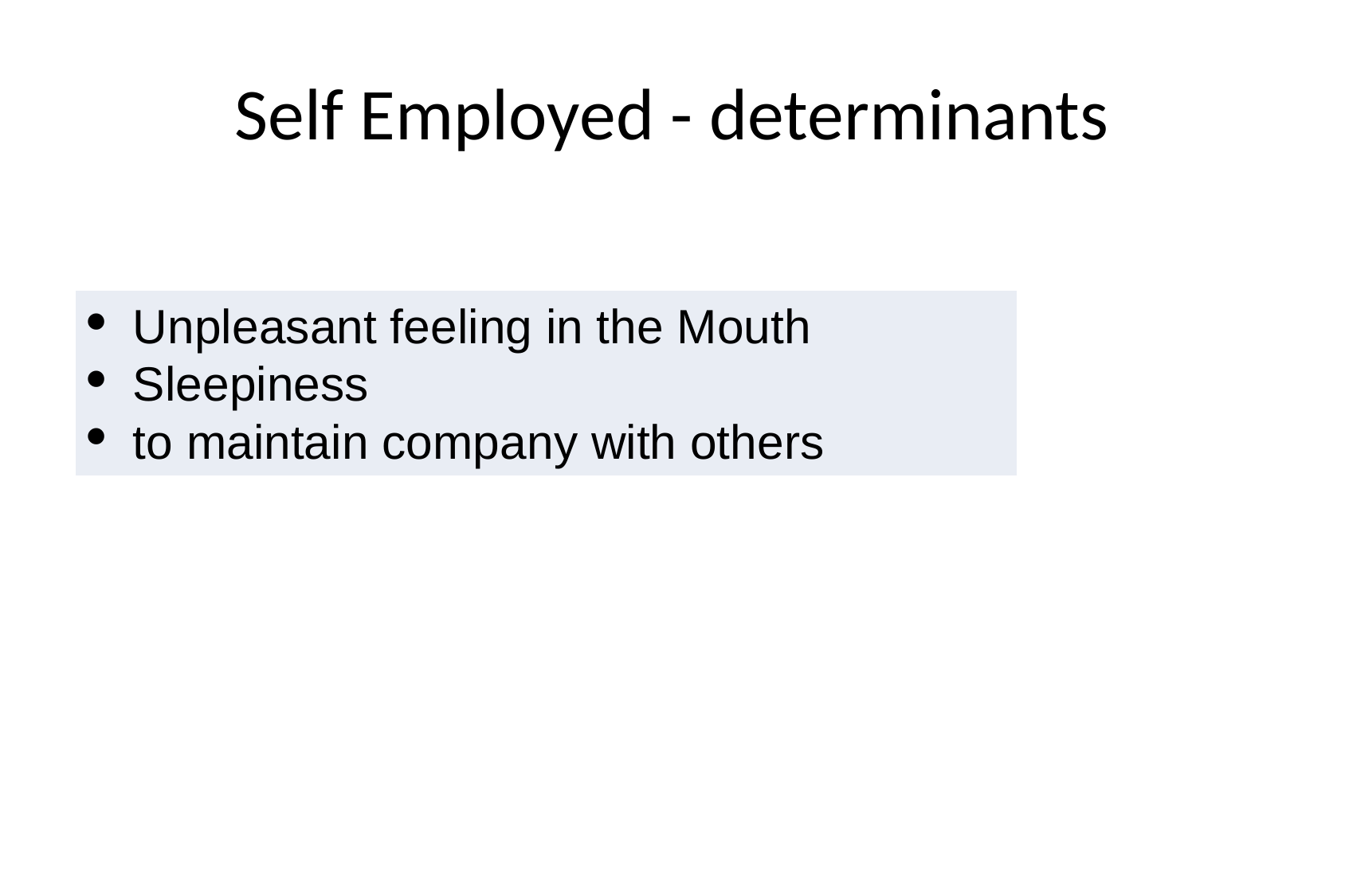

# Self Employed - determinants
| Unpleasant feeling in the Mouth Sleepiness to maintain company with others |
| --- |

## Slide 53
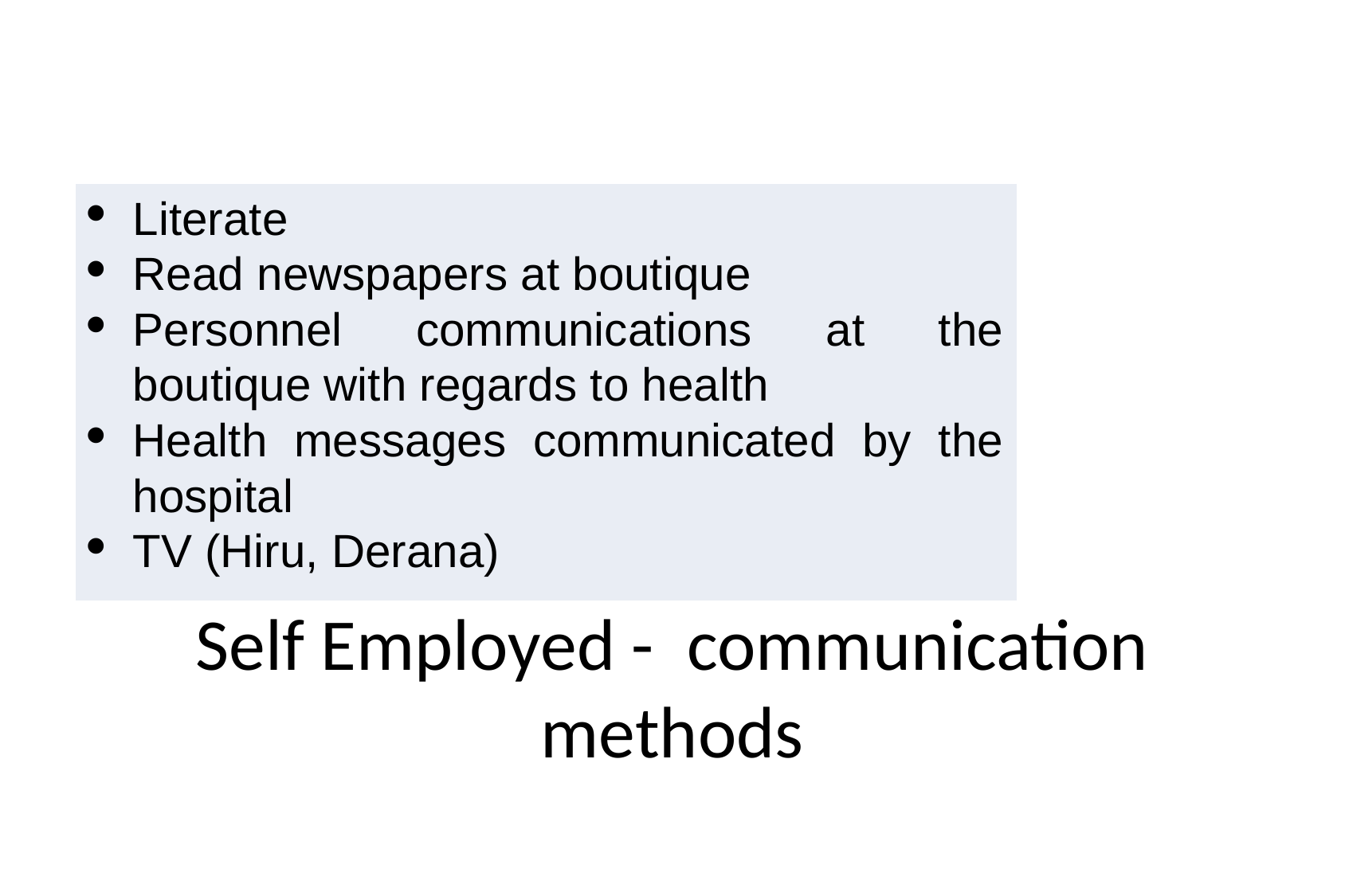

| Literate Read newspapers at boutique Personnel communications at the boutique with regards to health Health messages communicated by the hospital TV (Hiru, Derana) |
| --- |
# Self Employed - communication methods

## Slide 54
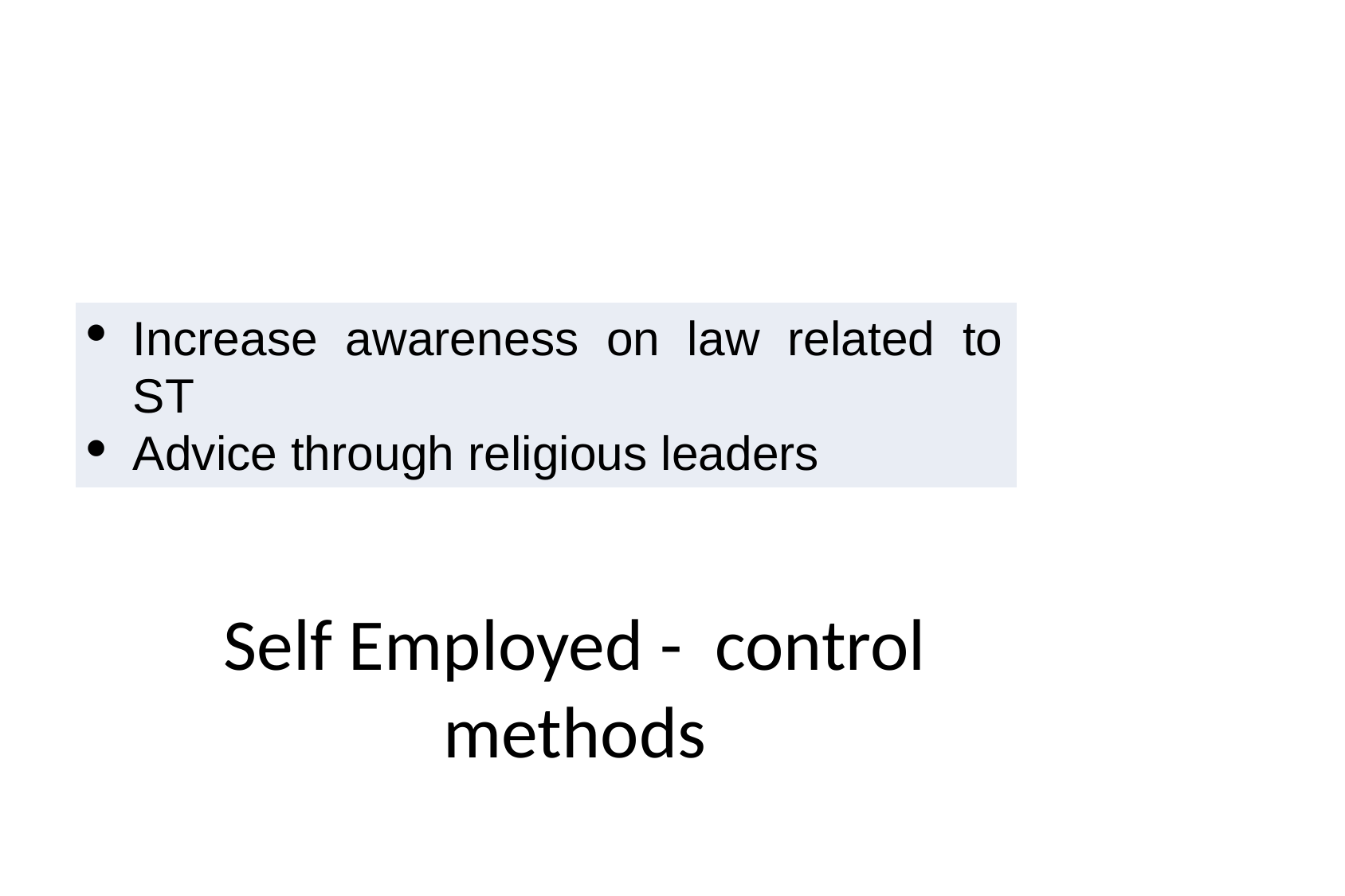

| Increase awareness on law related to ST Advice through religious leaders |
| --- |
# Self Employed - control methods

## Slide 55
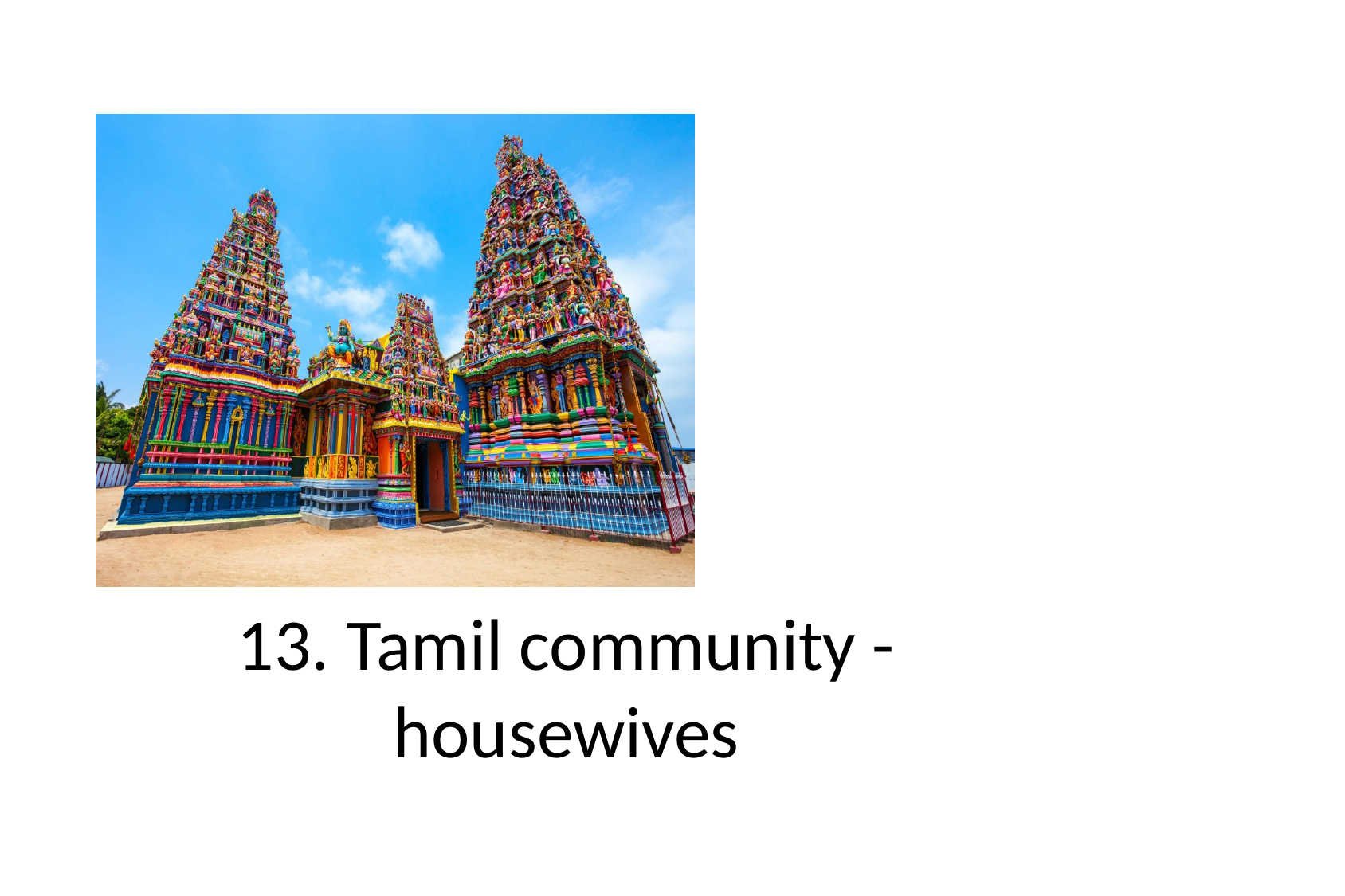

# 13. Tamil community - housewives

## Slide 56
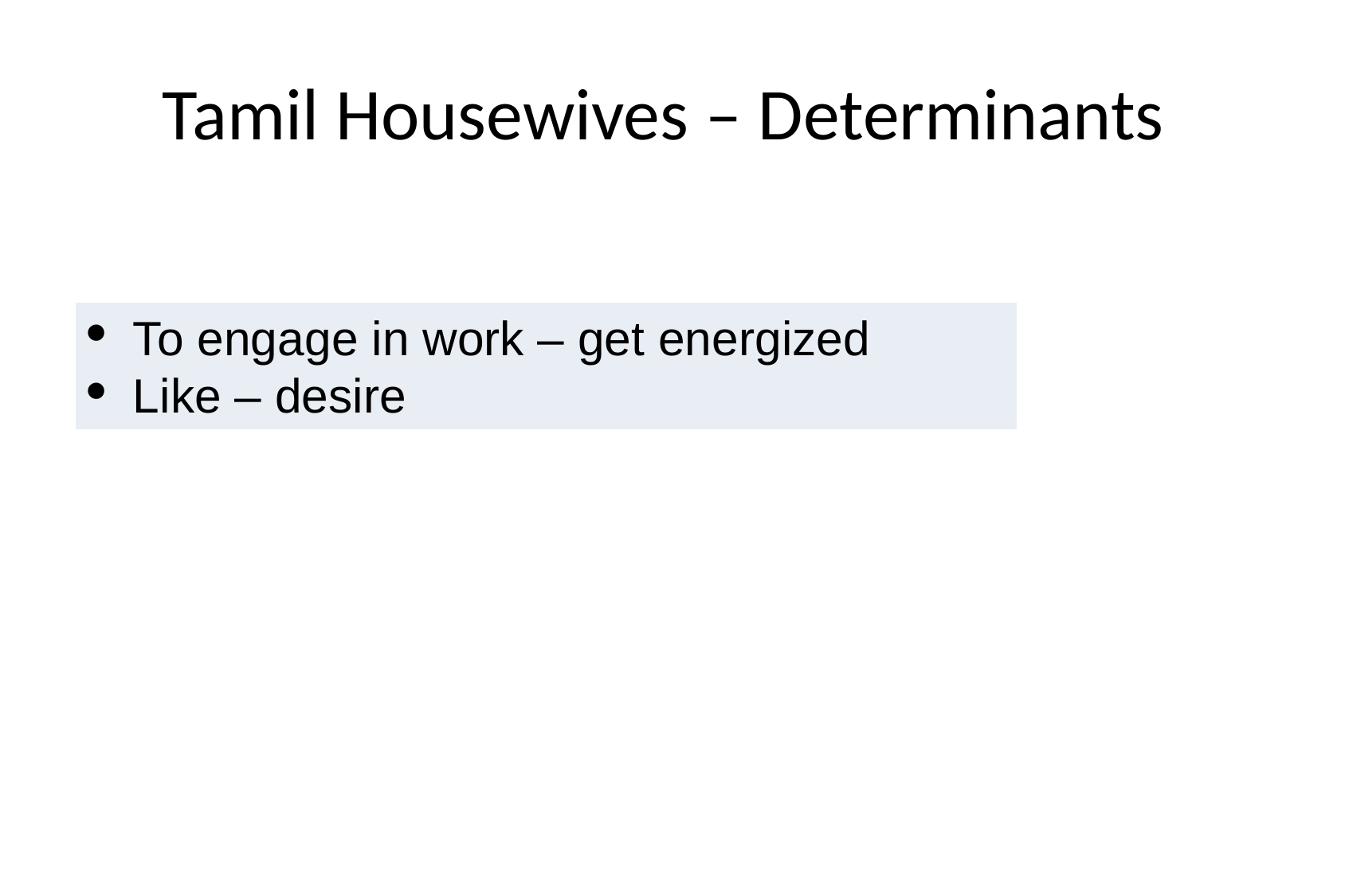

# Tamil Housewives – Determinants
| To engage in work – get energized Like – desire |
| --- |

## Slide 57
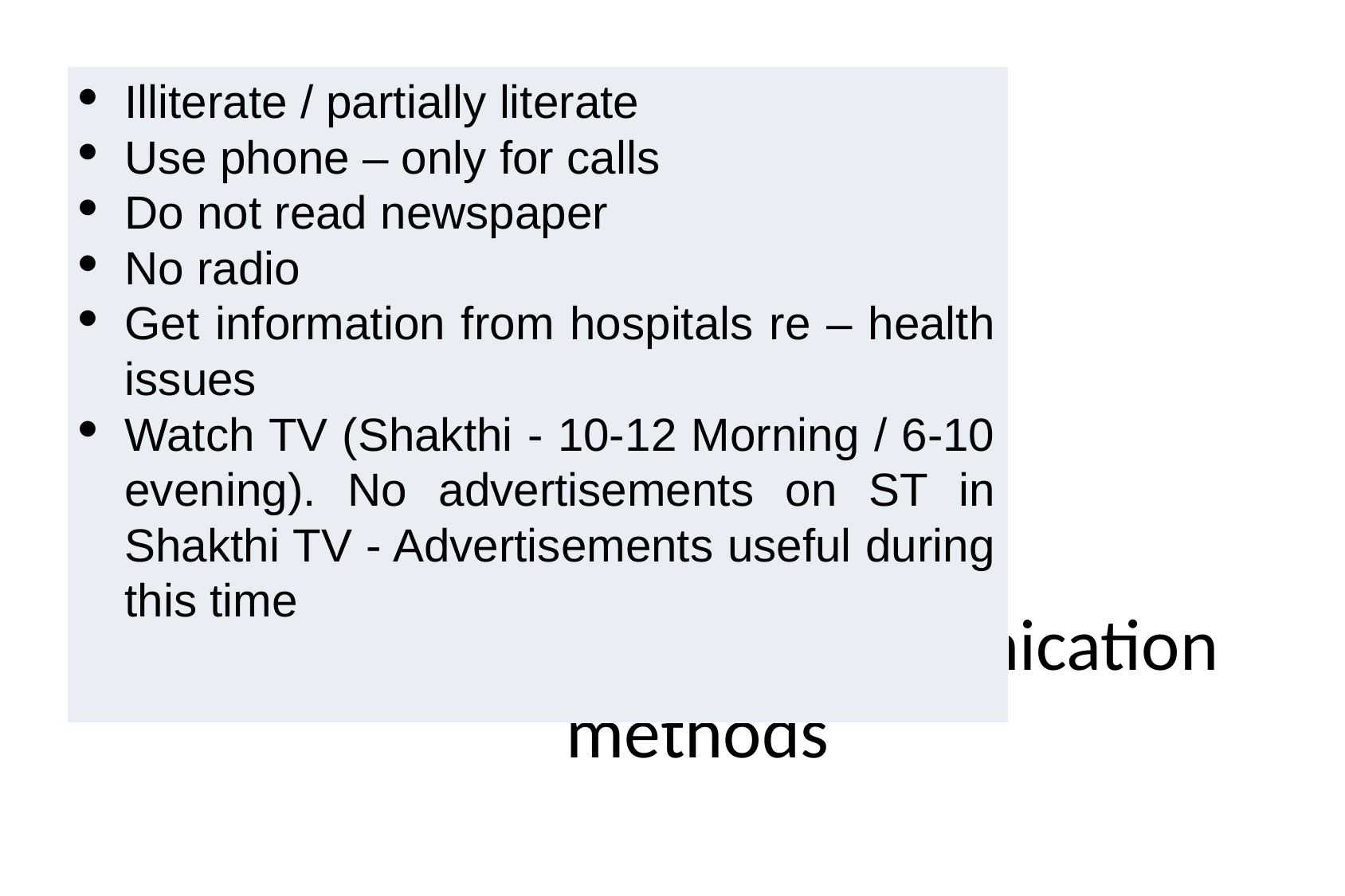

| Illiterate / partially literate Use phone – only for calls Do not read newspaper No radio Get information from hospitals re – health issues Watch TV (Shakthi - 10-12 Morning / 6-10 evening). No advertisements on ST in Shakthi TV - Advertisements useful during this time |
| --- |
# Tamil Housewives - communication methods

## Slide 58
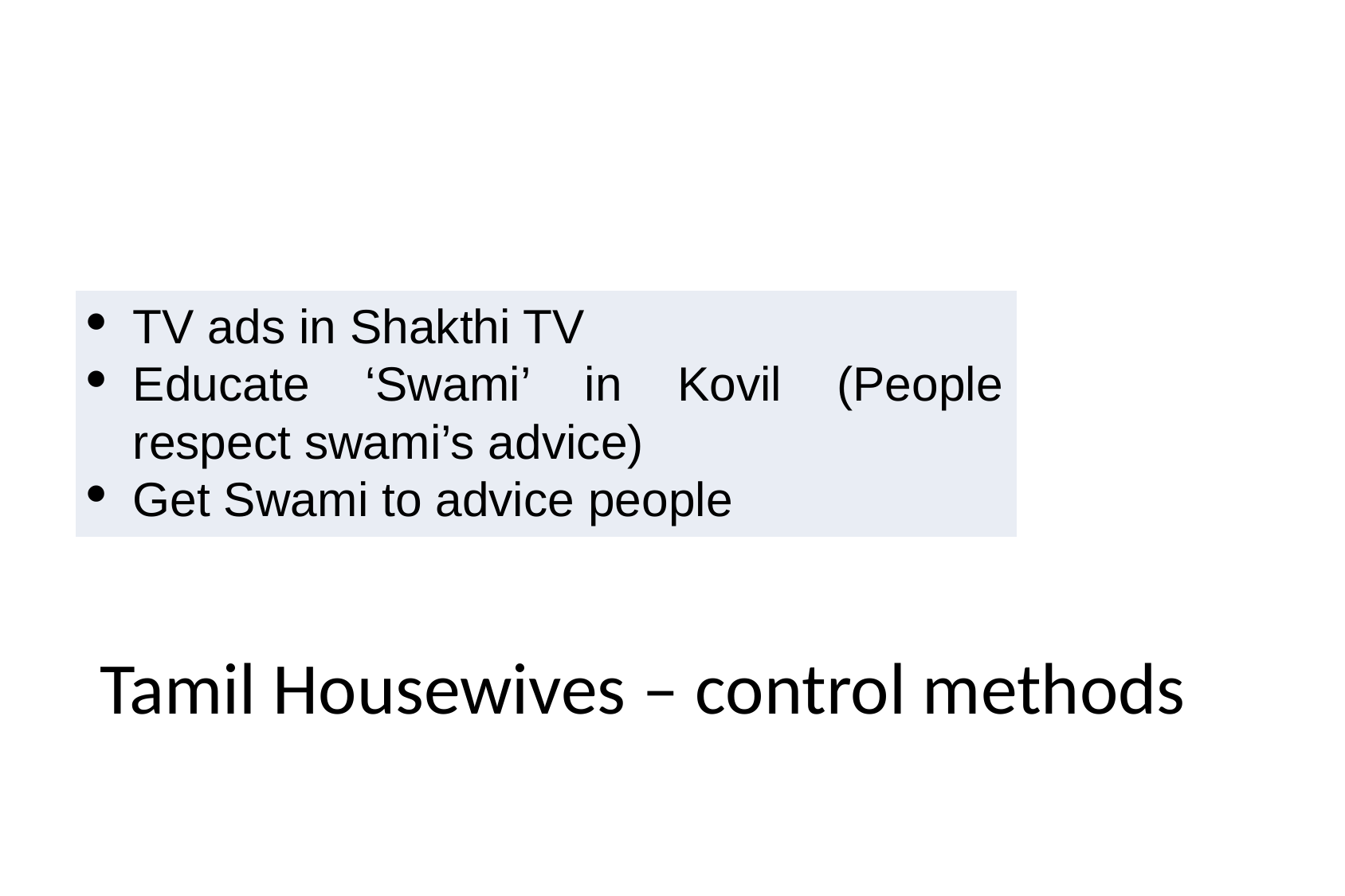

| TV ads in Shakthi TV Educate ‘Swami’ in Kovil (People respect swami’s advice) Get Swami to advice people |
| --- |
# Tamil Housewives – control methods

## Slide 59
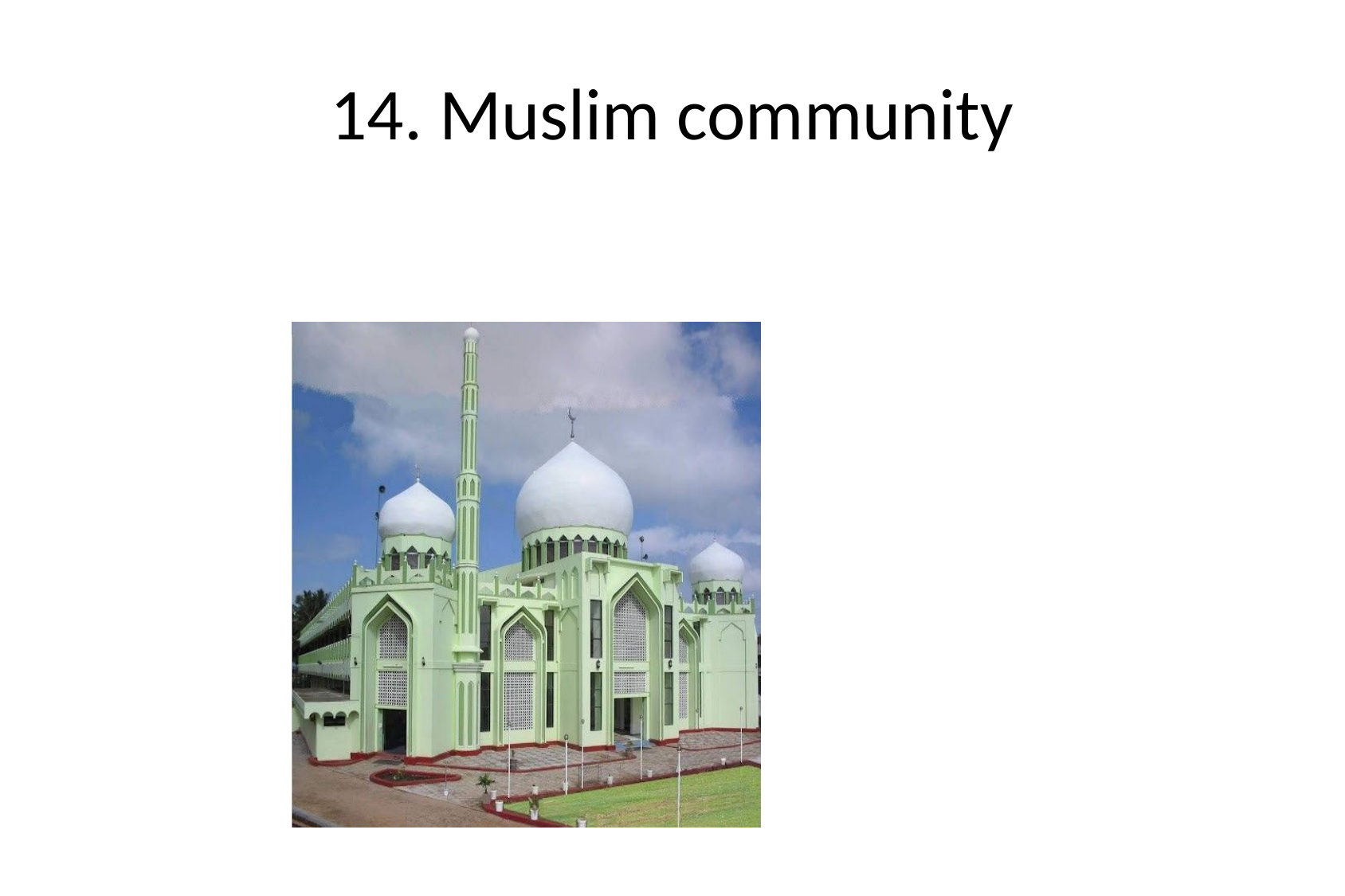

# 14. Muslim community

## Slide 60
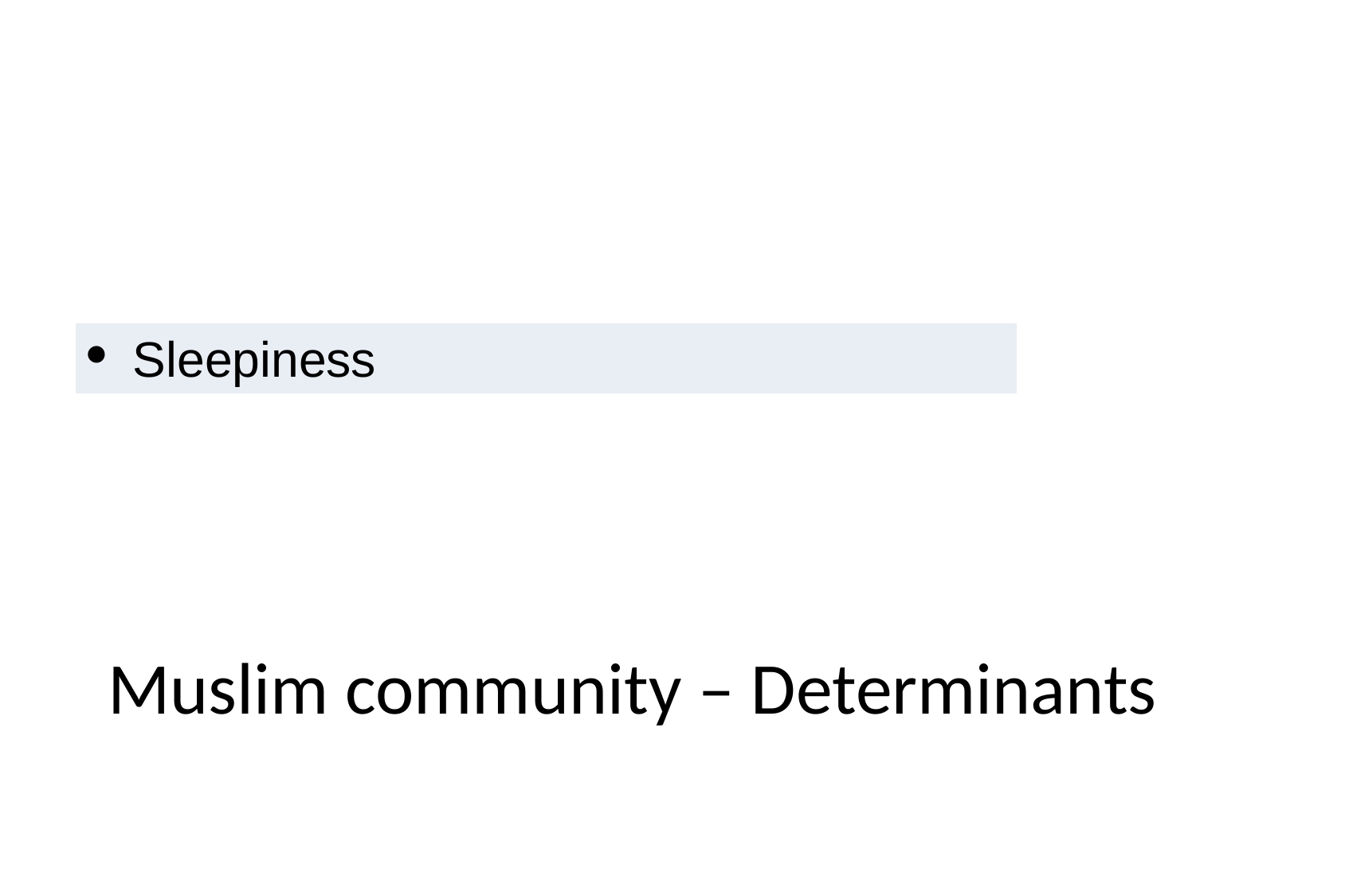

| Sleepiness |
| --- |
# Muslim community – Determinants

## Slide 61
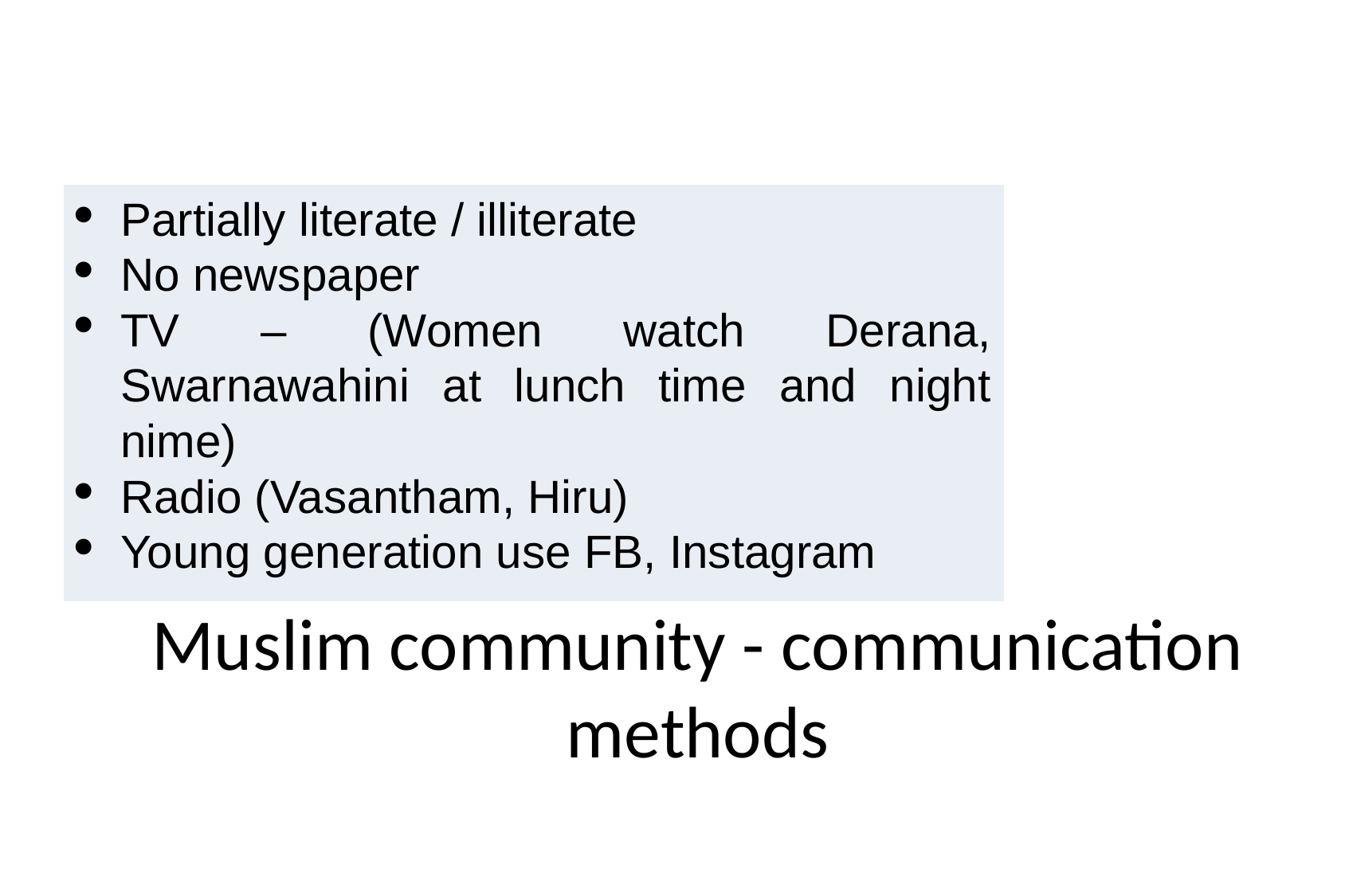

| Partially literate / illiterate No newspaper TV – (Women watch Derana, Swarnawahini at lunch time and night nime) Radio (Vasantham, Hiru) Young generation use FB, Instagram |
| --- |
# Muslim community - communication methods

## Slide 62
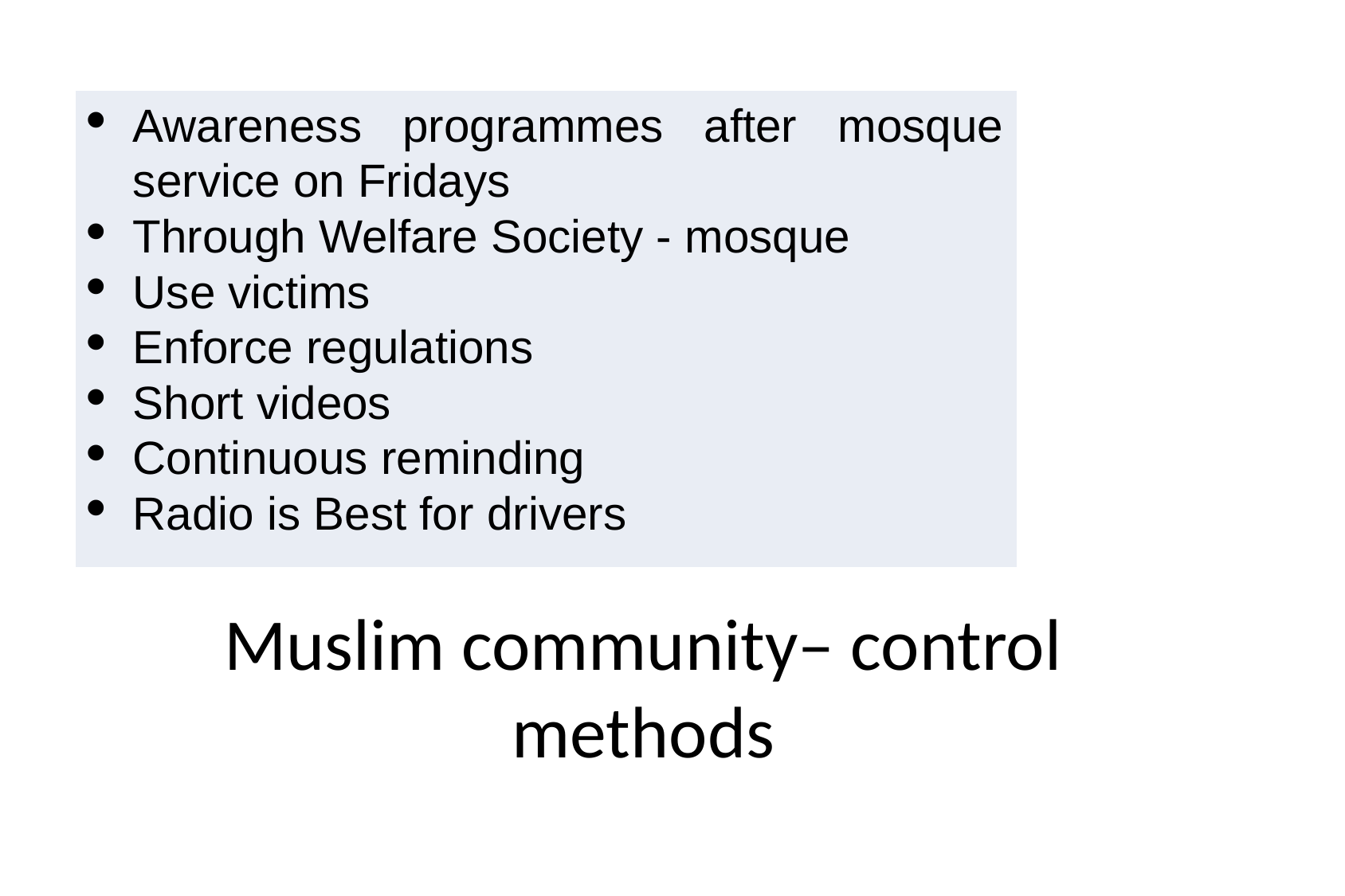

| Awareness programmes after mosque service on Fridays Through Welfare Society - mosque Use victims Enforce regulations Short videos Continuous reminding Radio is Best for drivers |
| --- |
# Muslim community– control methods

## Slide 63
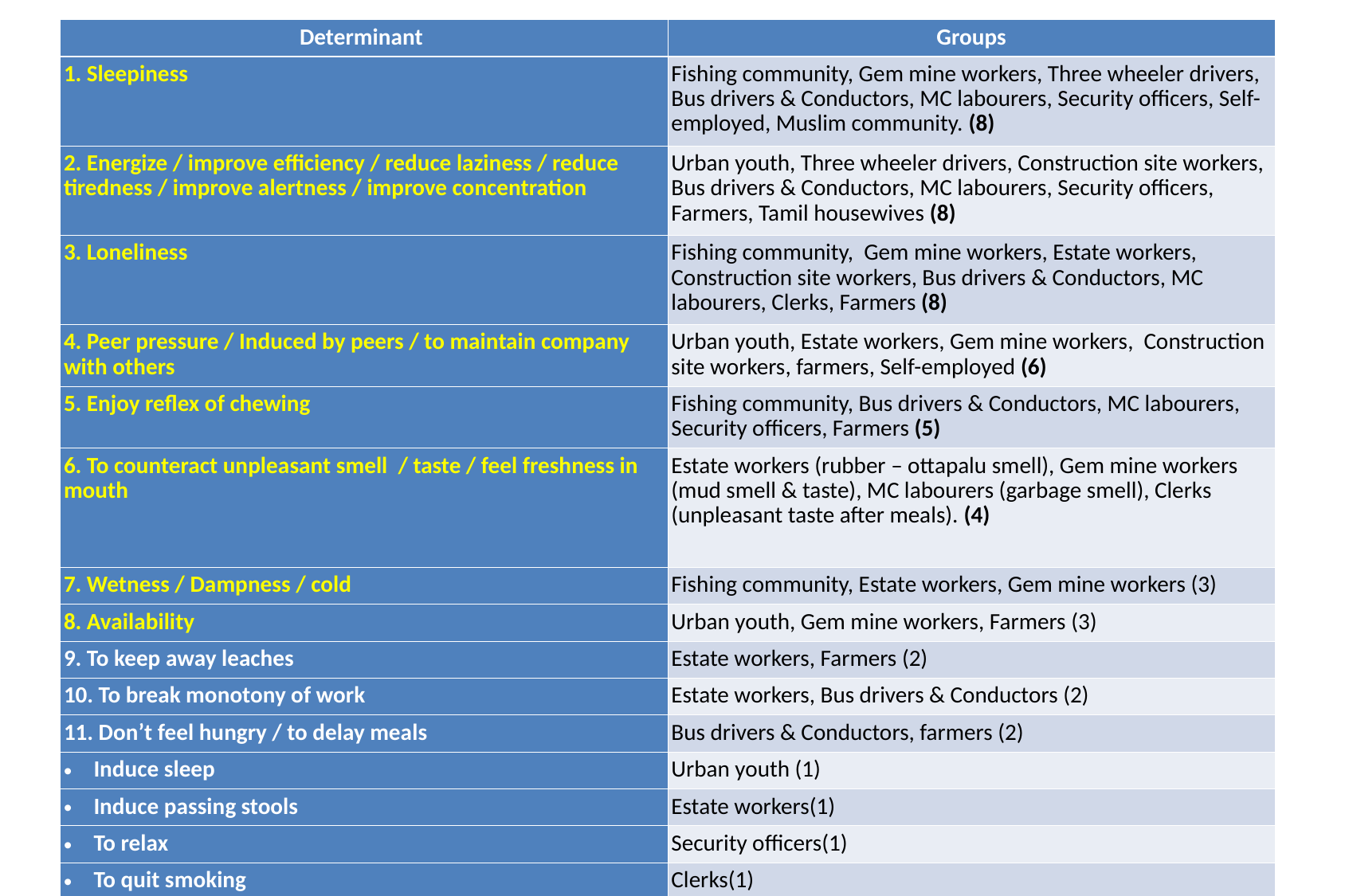

| Determinant | Groups |
| --- | --- |
| 1. Sleepiness | Fishing community, Gem mine workers, Three wheeler drivers, Bus drivers & Conductors, MC labourers, Security officers, Self-employed, Muslim community. (8) |
| 2. Energize / improve efficiency / reduce laziness / reduce tiredness / improve alertness / improve concentration | Urban youth, Three wheeler drivers, Construction site workers, Bus drivers & Conductors, MC labourers, Security officers, Farmers, Tamil housewives (8) |
| 3. Loneliness | Fishing community, Gem mine workers, Estate workers, Construction site workers, Bus drivers & Conductors, MC labourers, Clerks, Farmers (8) |
| 4. Peer pressure / Induced by peers / to maintain company with others | Urban youth, Estate workers, Gem mine workers, Construction site workers, farmers, Self-employed (6) |
| 5. Enjoy reflex of chewing | Fishing community, Bus drivers & Conductors, MC labourers, Security officers, Farmers (5) |
| 6. To counteract unpleasant smell / taste / feel freshness in mouth | Estate workers (rubber – ottapalu smell), Gem mine workers (mud smell & taste), MC labourers (garbage smell), Clerks (unpleasant taste after meals). (4) |
| 7. Wetness / Dampness / cold | Fishing community, Estate workers, Gem mine workers (3) |
| 8. Availability | Urban youth, Gem mine workers, Farmers (3) |
| 9. To keep away leaches | Estate workers, Farmers (2) |
| 10. To break monotony of work | Estate workers, Bus drivers & Conductors (2) |
| 11. Don’t feel hungry / to delay meals | Bus drivers & Conductors, farmers (2) |
| Induce sleep | Urban youth (1) |
| Induce passing stools | Estate workers(1) |
| To relax | Security officers(1) |
| To quit smoking | Clerks(1) |
| No other entertainment / leisure | Gem mine workers(1) |
#

## Slide 64
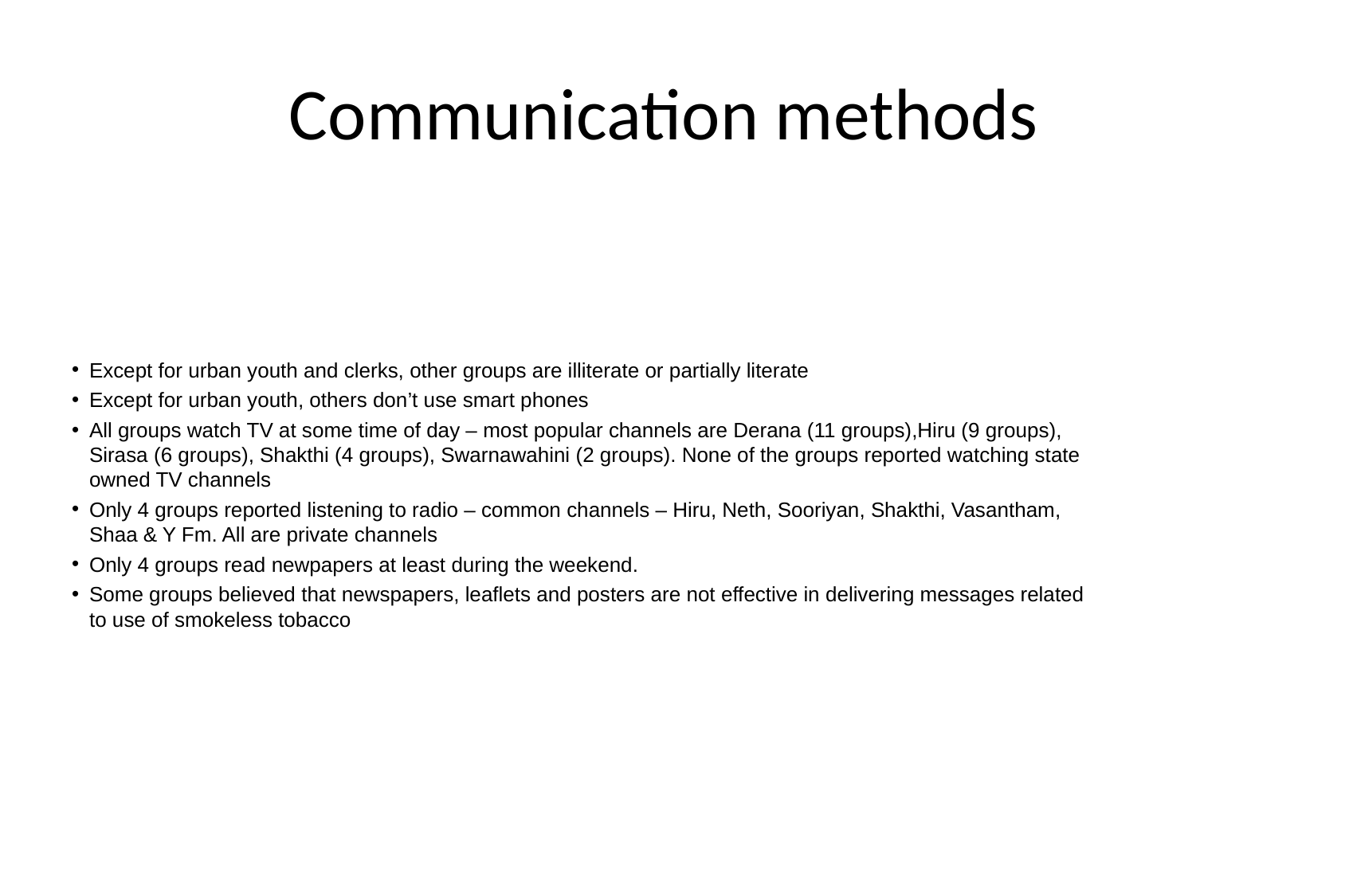

# Communication methods
Except for urban youth and clerks, other groups are illiterate or partially literate
Except for urban youth, others don’t use smart phones
All groups watch TV at some time of day – most popular channels are Derana (11 groups),Hiru (9 groups), Sirasa (6 groups), Shakthi (4 groups), Swarnawahini (2 groups). None of the groups reported watching state owned TV channels
Only 4 groups reported listening to radio – common channels – Hiru, Neth, Sooriyan, Shakthi, Vasantham, Shaa & Y Fm. All are private channels
Only 4 groups read newpapers at least during the weekend.
Some groups believed that newspapers, leaflets and posters are not effective in delivering messages related to use of smokeless tobacco

## Slide 65
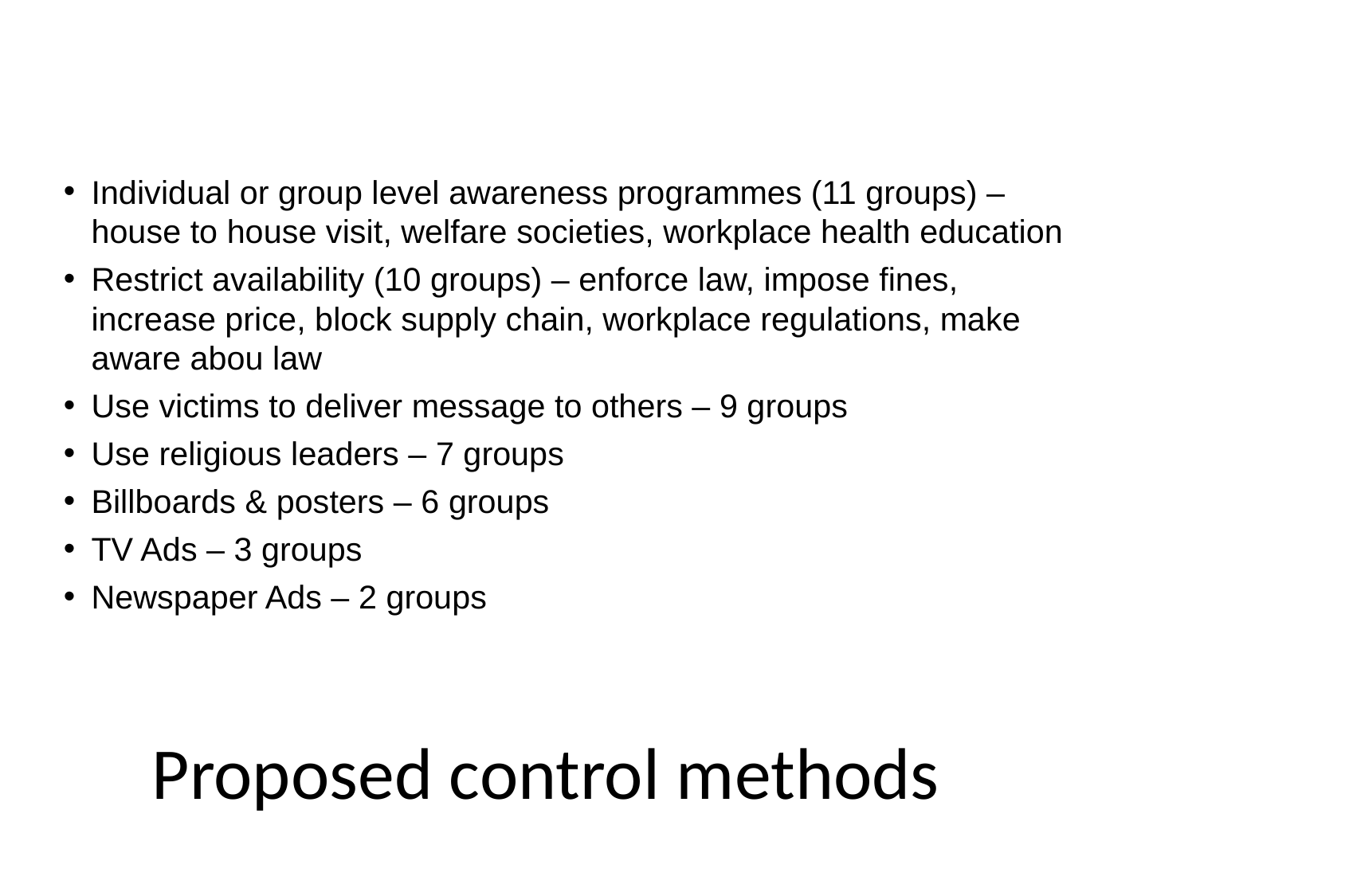

Individual or group level awareness programmes (11 groups) – house to house visit, welfare societies, workplace health education
Restrict availability (10 groups) – enforce law, impose fines, increase price, block supply chain, workplace regulations, make aware abou law
Use victims to deliver message to others – 9 groups
Use religious leaders – 7 groups
Billboards & posters – 6 groups
TV Ads – 3 groups
Newspaper Ads – 2 groups
# Proposed control methods

## Slide 66
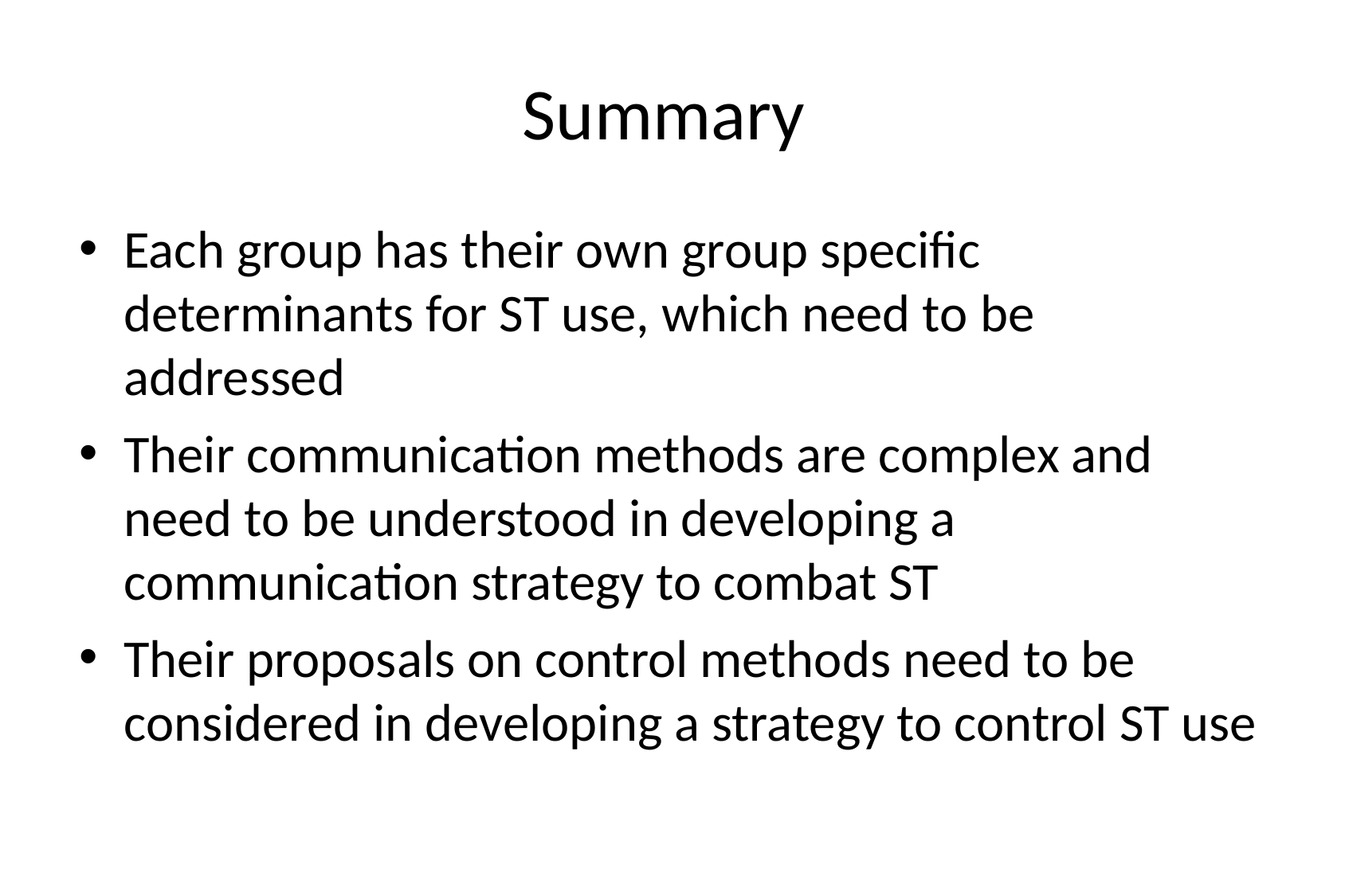

# Summary
Each group has their own group specific determinants for ST use, which need to be addressed
Their communication methods are complex and need to be understood in developing a communication strategy to combat ST
Their proposals on control methods need to be considered in developing a strategy to control ST use
