# Supplementary material for: Qualitative analysis to identify determinants of use among different occupational settings and channels of communication to address smokeless tobacco use in Sri Lanka
Source: PLOS Glob Public Health. 2023 Jan 4;3(1):e0001349. doi: 10.1371/journal.pgph.0001349 (PMC10022322; doi:10.1371/journal.pgph.0001349)
Supplement: Attachment — Submitted filename: Respose to reviewers 14082022.docx [file pgph.0001349.s005.docx]

PGPH-D-22-00450
Qualitative analysis to Identify determinants of use among different occupational settings and channels of communication to address smokeless tobacco use in Sri Lanka
PLOS Global Public Health

Journal Requirements:

1.  Please amend your detailed online Financial Disclosure statement. This is published with the article. It must therefore be completed in full sentences and contain the exact wording you wish to be published.: Yes rectified

a. State the initials, alongside each funding source, of each author to receive each grant.: National Authority on Tobacco and Alcohol was received the funding and authority covered the expenses for field visits: travel cost, accommodation and foods.
b. State what role the funders took in the study. If the funders had no role in your study, please state: “The funders had no role in study design, data collection and analysis, decision to publish, or preparation of the manuscript.”: Rectified and included in the page 4

2. Please update your online Competing Interests statement. If you have no competing interests to declare, please state: “The authors have declared that no competing interests exist.” : Rectified

3. In the online submission form, you indicated that “Field visit notes and script of audio tape recordings are available on request from the corresponding author.”. All PLOS journals now require all data underlying the findings described in their manuscript to be freely available to other researchers, either 1. In a public repository, 2. Within the manuscript itself, or 3. Uploaded as supplementary information.
This policy applies to all data except where public deposition would breach compliance with the protocol approved by your research ethics board. If your data cannot be made publicly available for ethical or legal reasons (e.g., public availability would compromise patient privacy), please explain your reasons by return email and your exemption request will be escalated to the editor for approval. Your exemption request will be handled independently and will not hold up the peer review process, but will need to be resolved should your manuscript be accepted for publication. One of the Editorial team will then be in touch if there are any issues.: All videos in Sinhala language and field notes were handwritten and not able to upload.

4. We have noticed that you have uploaded Supporting Information files, but you have not included a list of legends. Please add a full list of legends for your Supporting Information files after the references list.: Rectified

5. Please review your reference list to ensure that it is complete and correct. If you have cited papers that have been retracted, please include the rationale for doing so in the manuscript text, or remove these references and replace them with relevant current references. Any changes to the reference list should be mentioned in the rebuttal letter that accompanies your revised manuscript. If you need to cite a retracted article, indicate the article’s retracted status in the References list and also include a citation and full reference for the retraction notice.: Rechecked the references

Additional Editor Comments (if provided):


[Note: HTML markup is below. Please do not edit.]

Reviewers' comments:

Reviewer's Responses to Questions

**Comments to the Author**

1. Does this manuscript meet PLOS Global Public Health’s [publication criteria](https://journals.plos.org/globalpublichealth/s/criteria-for-publication)? Is the manuscript technically sound, and do the data support the conclusions? The manuscript must describe methodologically and ethically rigorous research with conclusions that are appropriately drawn based on the data presented.

Reviewer #1: Yes

Reviewer #2: Yes

2. Has the statistical analysis been performed appropriately and rigorously?

Reviewer #1: N/A

Reviewer #2: Yes

3. Have the authors made all data underlying the findings in their manuscript fully available (please refer to the Data Availability Statement at the start of the manuscript PDF file)?

The [PLOS Data policy](https://journals.plos.org/globalpublichealth/s/data-availability) requires authors to make all data underlying the findings described in their manuscript fully available without restriction, with rare exception. The data should be provided as part of the manuscript or its supporting information, or deposited to a public repository. For example, in addition to summary statistics, the data points behind means, medians and variance measures should be available. If there are restrictions on publicly sharing data—e.g. participant privacy or use of data from a third party—those must be specified.

Reviewer #1: Yes

Reviewer #2: Yes

4. Is the manuscript presented in an intelligible fashion and written in standard English?

PLOS Global Public Health does not copyedit accepted manuscripts, so the language in submitted articles must be clear, correct, and unambiguous. Any typographical or grammatical errors should be corrected at revision, so please note any specific errors here.

Reviewer #1: Yes

Reviewer #2: Yes

Please use the space provided to explain your answers to the questions above. You may also include additional comments for the author, including concerns about dual publication, research ethics, or publication ethics. (Please upload your review as an attachment if it exceeds 20,000 characters)

Reviewer #1: The authors have completed a qualitative study examining the self-described reasons for smokeless tobacco (SLT) use in Sri Lanka. The methods used are typical of this study style but must be interpreted with caution give then lack of representativeness of the subjects. While the study is interesting as written, I have several concerns that should be addressed.

• My most important critique is that I don’t have a sense of the comprehensiveness of the community sampling. The 13 communities could constitute 10% or 90% of Sri Lankan society. Second, I also have no idea how big one is versus another. In the current personation, I would have to assume that these communities are the same size. If there are large differences in the size of these communities, then that knowledge would inform the potential reach of any interventions.- We have selected occupational settings for community sampling which SLT use is more common. Each community are in difference population size in Sri Lankan context. I agree with your argument that qualitative research by using few of them will not accurately representation of the entire Sri Lankan subcommunity. However, occupational group voice could not be deviate very much.

• The authors should take care to ensure that the paper can be understood by non-Sri Lankans. For example, the authors use a term ‘plantation farming ’ and I am not sure what that means. It seems to be a type of plantation farming, but this needs to be explained more fully.- Remove the word estate workers and replace with Plantation farming workers

• In the intro, the authors described tobacco use in Sri Lankan, but they don’t clear state the entirety of tobacco use in the country. Specifically, I was unable to assess whether there are forms of tobacco between cigarettes and SLT as seen in many societies, such as waterpipe or cigar. And is marijuana smoking common? If SLT and cigarettes cover most tobacco use, please clearly state the percentages that use each individually versus dual use. The data from refence 5 left this unclear. :In Sri Lanka use of tobacco mainly in two forms: smoking and smokeless. Waterpipes are very rare and noticed in popular hotels in capital city of Sri Lanka. Cigar was popular in 50-60 years ago and now very rare. I am including those figures with percentage of dual use under the reference 5 in the introduction.

• I am not familiar with the term ‘Purposive sampling.’- We would like to retain both words Purposive and snowballing for two meanings: Examiners use their judgement to choose participants and from one contact got the clues to select next.

• The authors describe many different communities, but I found some of the labels unusual. In the results they sometime describe the communities as occupation, but that clearly doesn’t apply to ‘Muslim’ or ‘Urban young adults,’ I can see that they are all communities, so I would avoid the term occupation.- Yes I agree totally rectified as engaged communities

• Including the names of TV channels is not informative for an international audience.- Again a very important fact we discussed this fact among our experts, however if we donot include the names of our TV channels, study is not meaningfull. Other argument was in this global world anyone can access our channels through internet.

• The authors should be careful to use the same names in each instance. In the table we learn about “Urban young adults” but in the text there are also “Urban youth.” Are these the same people?- Yes rectified.

• The last sentence of the first paragraph of the discussion is an empty statement that doesn’t convey something one wouldn’t have guessed in advance: “These findings clearly demonstrate that when communications on preventing smokeless tobacco is planned, it needs to be carried out after careful consideration of many factors to obtain effective outcomes.”- Changed the content to be meaningful

• On page 19, an intervention is described as very costly, but there is consistent comparison of costs for different interventions, and this is an important factor. If the most effective message is costly, it still may be cost effective. In many societies, media is required to carry Public Service Announcements, and this may mitigate some costs. Since this not a cost effectiveness paper, I suggest you minimize discussion of costs.- Totally agreed however we quoted our publication on cost to justified that money is not a waste.

• On page 20, the authors write “approached effectively by religious leaders.” Although suggested by participants, the authors have not gauged the effectiveness of any of these approaches.- Removed the word ‘effectively’

• On page 20, the authors suggest there may not be an effective response to claims of the utility of SLT for “to address loneliness or to prevent a bad odor.” I’m sure there are alternative to SLT for these issues, so I don’t think this should be presented as hopeless.- Rectified in page 20

Reviewer #2: The manuscript is technically sound and the data does support the conclusion made in the manuscript. The manuscript uses qualitative analysis and applied thematic analysis across occupational groups.

However there are few questions for the authors:

1. There is no framework given of how the number of interviews and FDGs were finalized. Was it based on some methodology? Number of in-depth interviews were decided based on saturation levels reached. Focus group discussions were limited to one in each group except in garbage collectors due to feasibility and logistics issues.

2. First paragraph of the results section states "Females were represented among plantation farming workers, garbage collectors of the local civil administration and Muslim communities and income remain constant for each occupation". However, no comparison is made by gender in the thematic analysis?- Some engaged communities of occupational settings were only male. Therefore, no thematic analysis was carried out based on gender.

3. More emphasis can be done on the factors which are overlapping in different occupations group. As it is easy for the regulatory body to implement interventions which are common to many occupational groups. After which specific reforms can be introduced for each group. Following sentence was added to the discussion: Implementation authorities could identify and prioritize interventions that are common to different occupational group

6. PLOS authors have the option to publish the peer review history of their article ([what does this mean?](https://journals.plos.org/globalpublichealth/s/editorial-and-peer-review-process#loc-peer-review-history)). If published, this will include your full peer review and any attached files.


**Do you want your identity to be public for this peer review?** If you choose “no”, your identity will remain anonymous but your review may still be made public.


For information about this choice, including consent withdrawal, please see our [Privacy Policy](https://www.plos.org/privacy-policy).

Reviewer #1: No

Reviewer #2: No

[NOTE: If reviewer comments were submitted as an attachment file, they will be attached to this email and accessible via the submission site. Please log into your account, locate the manuscript record, and check for the action link "View Attachments". If this link does not appear, there are no attachment files.]

While revising your submission, please upload your figure files to the Preflight Analysis and Conversion Engine (PACE) digital diagnostic tool, <https://pacev2.apexcovantage.com/>. PACE helps ensure that figures meet PLOS requirements. To use PACE, you must first register as a user. Registration is free. Then, login and navigate to the UPLOAD tab, where you will find detailed instructions on how to use the tool. If you encounter any issues or have any questions when using PACE, please email PLOS at [figures@plos.org](mailto:figures@plos.org). Please note that Supporting Information files do not need this step.
